# *Cladosporium* from caves of the Brazilian savannah (Cerrado) and the description of six new species

**DOI:** 10.3897/imafungus.17.191673

**Published:** 2026-06-03

**Authors:** Pedro H. Félix-Oliveira, José F. S. A. Prazeres, Layanne O. Ferro, Ana C. A. Antunes, Caroline F. Lima, Dominnyke S. S. Neves, Emily O. Fonseca, Lorena S. Miranda, Renata S. Momoli, Cristina M. Souza-Motta, Konstanze Bensch, Pedro W. Crous, Jadson D. P. Bezerra

**Affiliations:** 1 Programa de Pós-Graduação em Biologia da Relação Parasito-Hospedeiro (PPGBRPH), Instituto de Patologia Tropical e Saúde Pública, Universidade Federal de Goiás, Rua 235, s/n, Setor Universitário, CEP: 74605-050, Goiânia, GO, Brazil Laboratório de Micologia (LabMicol), Departamento de Biociências e Tecnologia, Instituto de Patologia Tropical e Saúde Pública, Universidade Federal de Goiás Goiânia Brazil; 2 Laboratório de Micologia (LabMicol), Departamento de Biociências e Tecnologia, Instituto de Patologia Tropical e Saúde Pública, Universidade Federal de Goiás, Rua 235, s/n, Setor Universitário, CEP: 74605-050, Goiânia, GO, Brazil Programa de Pós-Graduação em Biologia da Relação Parasito-Hospedeiro (PPGBRPH), Instituto de Patologia Tropical e Saúde Pública, Universidade Federal de Goiás Goiânia Brazil; 3 Departamento de Micologia Prof. Chaves Batista, Universidade Federal de Pernambuco, Av. Prof. Moraes Rego, s/n, Centro de Biociências, Cidade Universitária, CEP: 50670-901, Recife, PE, Brazil Instituto de Estudos Socioambientais (IESA), Universidade Federal de Goiás Goiânia Brazil; 4 Programa de Pós-Graduação em Biologia de Fungos (PPGBF), Departamento de Micologia Prof. Chaves Batista, Universidade Federal de Pernambuco, Av. Prof. Moraes Rego, s/n, Centro de Biociências, Cidade Universitária, CEP: 50670-901, Recife, PE, Brazil Forestry & Agricultural Biotechnology Institute (FABI), University of Pretoria Pretoria South Africa; 5 Laboratório de Geomorfologia, Pedologia e Geografia Física (LABOGEF), Instituto de Estudos Socioambientais (IESA), Universidade Federal de Goiás, Av. Esperança, s/n, Samambaia, CEP: 74001-970, Goiânia, GO, Brazil Westerdijk Fungal Biodiversity Institute Utrecht Netherlands; 6 Westerdijk Fungal Biodiversity Institute, Uppsalalaan 8, Utrecht, 3584 CT, Netherlands Programa de Pós-Graduação em Biologia de Fungos (PPGBF), Departamento de Micologia Prof. Chaves Batista, Universidade Federal de Pernambuco Recife Brazil; 7 Department of Biochemistry, Genetics & Microbiology, Forestry & Agricultural Biotechnology Institute (FABI), University of Pretoria, Pretoria, South Africa Departamento de Micologia Prof. Chaves Batista, Universidade Federal de Pernambuco Recife Brazil; 8 Microbiology, Department of Biology, Utrecht University, Padualaan 8, Utrecht, 3584 CH, Netherlands Microbiology, Department of Biology, Utrecht University Utrecht Netherlands

**Keywords:** Cave fungi, fungal taxonomy, new taxa, phylogeny, speleomycology

## Abstract

Caves are important but understudied reservoirs of fungal diversity, particularly for genera such as *Cladosporium* (*Cladosporiales*, *Dothideomycetes*). This study aimed to characterise the richness of *Cladosporium* species from six caves in the Brazilian savannah (Cerrado) using an integrative approach combining morphology and multi-locus phylogenetic analyses (*ACT*, ITS, *RPB2*, *TEF1-α*, and *TUB*). To improve species recognition in *Cladosporium*, selected ex-type strains were studied, for which *RPB2* and *TUB* barcodes were also sequenced. Species were delimited using morphology and phylogenetic inferences, supplemented by the pairwise homoplasy index (PHI) test to evaluate species boundaries. Twenty-three species were identified among 94 *Cladosporium* isolates. The polyphasic approach resulted in the description of six new species: five in the *C. cladosporioides* complex (*C. carsi*, *C. lacerdae*, *C. mambaiense*, *C. nogueirae*, and *C. propiciense*) and one in the *C. sphaerospermum* complex (*C. mesquitapaivae*). Additionally, analyses based on morphology, genealogical concordance phylogenetic species recognition (GCPSR), and the PHI test resulted in the synonymisation of *C. ribis* and *C. speluncae* under *C. bambusicola*, *C. brigadeirense* under *C. puris*, and *C. marinisedimentum* under *C. sphaerospermum*. Among the previously known species identified, 11 represent new records for cave environments, with *C. bambusicola* being the most frequent (29.17%). These findings substantially expand the known diversity of *Cladosporium* in Neotropical caves, highlighting the Brazilian Cerrado as a hotspot of mycodiversity. Furthermore, *ACT*, *RPB2*, *TEF1-α*, and *TUB* are proposed as the primary phylogenetic barcodes for distinguishing closely related *Cladosporium* species, particularly within the *C. cladosporioides* complex. This study further emphasises the necessity of using a polyphasic approach to achieve accurate taxonomic resolution among *Cladosporium* species, trace their distribution in caves, and help to understand their ecology.

## Introduction

The cave environment is considered a hotspot for fungal diversity, owing to abiotic factors such as stable temperature and humidity that favour the development of species of medical and ecological importance (Barbosa et al. 2025; Prazeres et al. 2025). In these extreme, nutrient-poor conditions, such stability facilitates the production of specialised biomolecules and enzymes, resulting in unique secondary metabolites with high bioprospecting potential (Barbosa et al. 2025). In Brazil, mycospeleology remains neglected. Despite approximately 30,000 caves being registered in the Brazilian territory (CANIE 2026), only 30 caves have been studied for the presence of fungi. Nevertheless, these explored caves were found to harbour approximately 300 fungal genera (Prazeres et al. 2025), accounting for approximately 45% of the fungal genera reported in caves worldwide (Vanderwolf et al. 2013; Zhang et al. 2021). The most common genera in Brazilian caves are *Penicillium*, *Aspergillus*, and *Cladosporium* (Prazeres et al. 2025), with the latter represented by 12 species reported from these environments, including new species described from caves in the Cerrado and Caatinga biomes (Carvalho et al. 2022; Pereira et al. 2022; Dutra et al. 2023).

The genus *Cladosporium* (*Cladosporiales*, *Cladosporiaceae*) has been intensively studied in recent years due to its importance in various biological niches (Farwell et al. 2024; Li et al. 2024; Liu et al. 2024). *Cladosporium* spp. are cosmopolitan and have been found in association with several substrates and hosts on the surface and in underground environments, being reported as saprobes (Zalar et al. 2007) and phytopathogens (Bhartiya et al. 2022; Yang et al. 2023) and also associated with insects (Nicoletti et al. 2024), responsible for causing opportunistic human infections (Sandoval-Denis et al. 2016), found in indoor environments (Karalti and Çolakoğlu 2012), and used in biotechnology (Belabess et al. 2024). Recently, Biagioli et al. (2023) demonstrated that *Cladosporium* is one of the most abundant genera in caves globally, influenced by climate change conditions and other external environmental factors. Interestingly, *Cladosporium* species have also been reported as key players in extreme ecosystems (Simonetti et al. 2024).

The genus *Cladosporium* is presently divided into three species complexes (*C. cladosporioides*, *C. herbarum*, and *C. sphaerospermum*), which include more than 900 names (Lee et al. 2023; https://www.mycobank.org). Morphologically, species of *Cladosporium* are often very similar, so using a polyphasic approach for identification is the most appropriate way to study their diversity. Several studies showed that the use of morphological features and phylogeny based on multi-locus analyses [internal transcribed spacer regions (ITS), partial fragments of actin (*ACT*), and translation elongation factor 1-alpha (*TEF1-α*)] is important for studying closely related species and defining their species complexes (Poli et al. 2024). This complexity was also recently highlighted through the analysis of 42 *Cladosporium* genomes (Zheng et al. 2025), demonstrating that the *C. sphaerospermum* complex is a polyphyletic group. However, in most studies of cave fungi in Brazil, including those of *Cladosporium* species, morphology has been the main feature used for identifying these fungi (Taylor et al. 2013, 2014). This study highlights the importance of combining morphological analysis and phylogeny of different DNA markers to investigate the diversity of *Cladosporium* species in Brazilian caves, which are recognised as fungal hotspots in the country.

The Cerrado biome, a Brazilian savannah and biodiversity hotspot, contains more than 12,008 recorded caves (ICMBio/CECAV 2025), making it an environment rich in endemic species and essential for ecological and conservation studies. Studies that survey the biodiversity of the Cerrado are important due to the high level of endemism and, in particular, the significant increase in species extinction rates (Klink and Machado 2005). *Cladosporium* is one of the most common genera in Cerrado caves (Taylor et al. 2013, 2014; Dutra et al. 2023; Prazeres et al. 2025).

A current project is being conducted to estimate fungal diversity in several caves of Brazilian biomes. This paper presents the results of *Cladosporium* species found in six caves of the Cerrado. In this study, a collection of 94 *Cladosporium* isolates, obtained from airborne fungi and the soil of Cerrado caves, was assessed to verify species richness using morphology and a five-gene (*ACT*, ITS rDNA, *RPB2*, *TEF1-α*, and *TUB*) sequence dataset based on the genealogical concordance phylogenetic species recognition (GCPSR) concept and the complementary use of the pairwise homoplasy index (PHI) to evaluate species boundaries. In addition, the inclusion of *TUB* and *RPB2* sequences in phylogenetic analyses is proposed to resolve closely related *Cladosporium* species. This study highlights the presence of *Cladosporium* spp. in caves, thereby expanding knowledge of their distribution in underground environments.

## Materials and methods

### Caves

**Figure 1.**
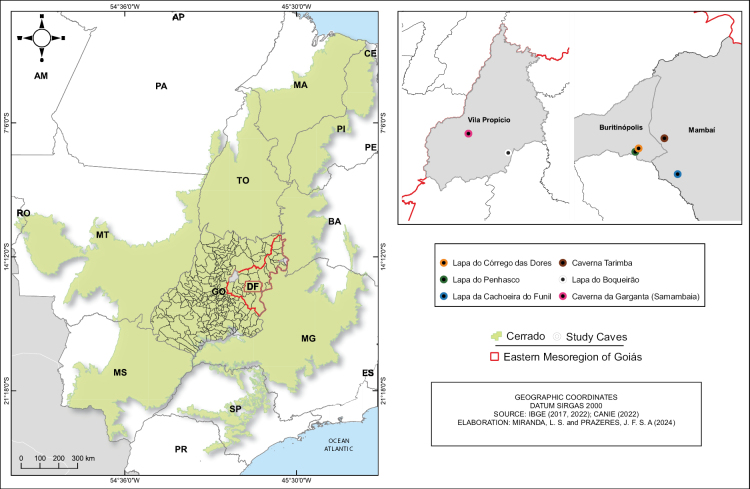
The geographical location of caves of the Brazilian Cerrado visited for the isolation of *Cladosporium* species.

**Figure 2.**
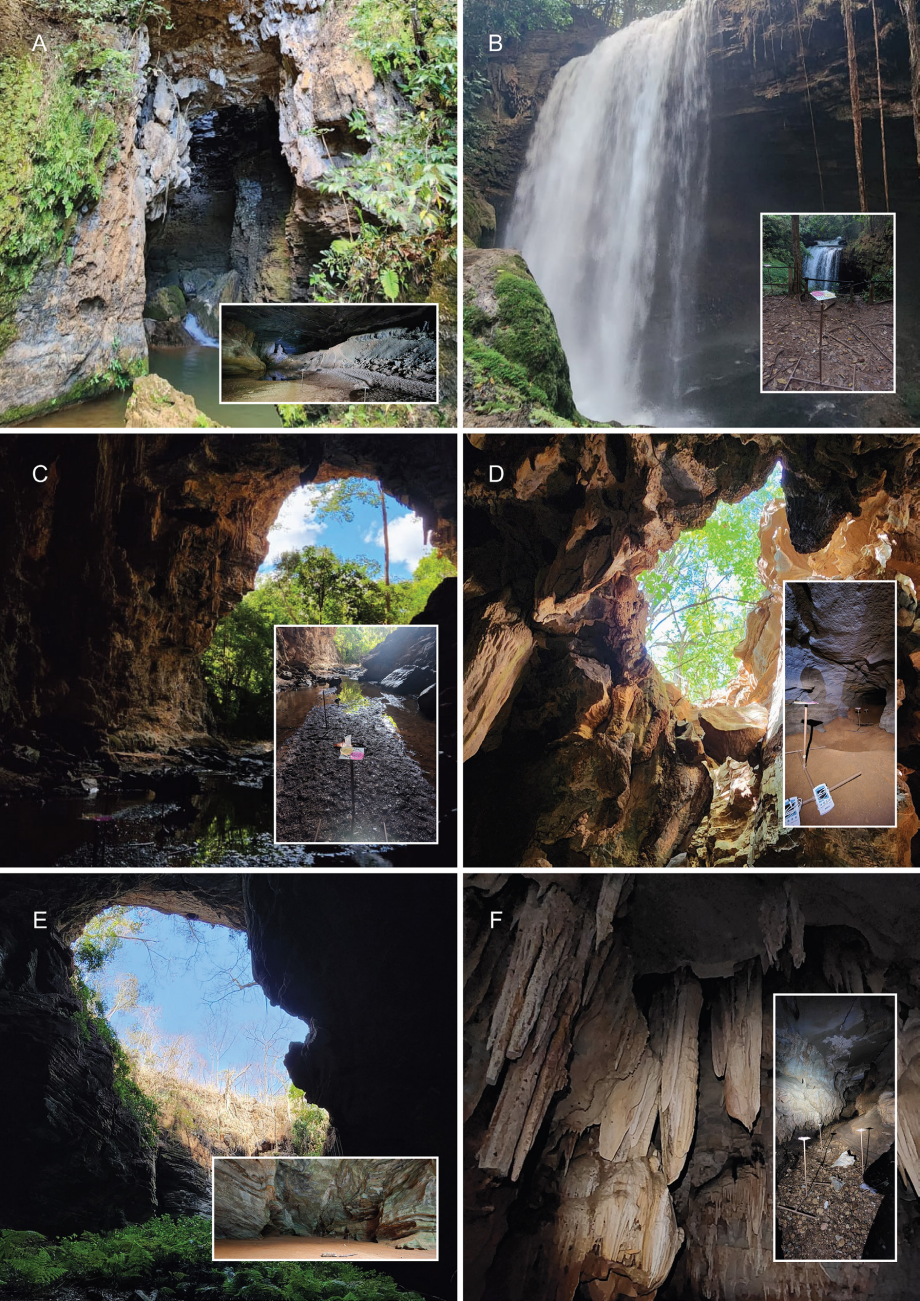
Overview of Brazilian Cerrado caves visited for the isolation of fungal species and a highlight of sampling collection points. **A**. Lapa do Penhasco. **B**. Lapa da Cachoeira do Funil. **C**. Lapa do Córrego das Dores. **D**. Gruna da Tarimba. **E**. Garganta (Samambaia). **F**. Lapa do Boqueirão. Photos were taken by J.D.P. Bezerra, P.H. Félix-Oliveira, and J.F.S.A. Prazeres.

The six caves analysed in this study are located in the eastern mesoregion of the state of Goiás, Brazil (Figs 1, 2, Suppl. material 9: table SS1). Four of them (Lapa do Córrego das Dores, Lapa da Cachoeira do Funil, Lapa do Penhasco, and Gruna Tarimba) are situated within the Área de Proteção Ambiental (APA) Nascentes do Rio Vermelho, a conservation unit with a total area of 1,763.24 km^2^. This APA has more than 150 registered caves and is mainly located in the municipality of Mambaí, with parts of its territory also covering the municipalities of Buritinópolis, Damianópolis, and Posse (CANIE 2026). The APA is on the border with an agricultural frontier expansion region located in the state of Bahia (Esbérard et al. 2005; Momoli et al. 2022). The APA Nascentes do Rio Vermelho has a sub-humid tropical climate, classified as Aw according to the Köppen classification, with an average annual temperature of 24 °C and rainfall ranging between 1,250 and 1,750 mm per year. The rainfall regime is characterised by two distinct seasons: the dry season, which spans from May to September, with a minimum rainfall of less than 4 mm in July; and the rainy season, from November to March, with monthly averages exceeding 200 mm (Momoli et al. 2022). Regarding vegetation, the APA follows the typical pattern of the Cerrado biome, characterised by a high diversity of phytophysiognomies. Among the main vegetation types present are cerradão, veredas, and matas de galeria, which play a fundamental role in maintaining local biodiversity (Esbérard et al. 2005).

The other two caves analysed in this study (Lapa do Boqueirão and Garganta [Samambaia]) are located in the municipality of Vila Propício, a region known for its high concentration of natural caves, with more than 80 registered in its territory. These caves represent approximately 25% of the municipality’s total area, underscoring their significant speleological importance at the state level. The municipality of Vila Propício has a semi-humid tropical climate, with an average annual temperature ranging between 22 °C and 26 °C. The rainfall regime is marked by a well-defined rainy season, with annual precipitation reaching up to 1,500 mm (https://portal.inmet.gov.br). Also located in the Cerrado biome, the municipality harbours vegetation formations similar to those found in the APA Nascentes do Rio Vermelho, such as cerradão, typical cerrado, mata de galeria, and other characteristic phytophysiognomies of the biome. Although the municipality of Vila Propício has an extensive number of caves (CANIE 2026), it does not yet have a formally established conservation unit to protect this speleological complex, which represents a challenge for the conservation of subterranean environments. Both Vila Propício and the APA Nascentes do Rio Vermelho practise speleotourism, which has become an economic alternative, promoting the visibility of natural heritage and encouraging actions aimed at conserving the caves (Freitas et al. 2019). Field sampling was authorised by the Ministério do Meio Ambiente (MMA)/Instituto Chico Mendes de Conservação da Biodiversidade (ICMBio); permit number: 82254-5.

### Fungal isolation from caves

Collection points were established inside and outside each cave, determined based on their size and structural characteristics (Suppl. material 9: table SS1). The sedimentation method was used on the culture medium contained in Petri dishes. This method consisted of exposing 90 mm diameter Petri dishes containing 20 mL of the culture media Dichloran Rose Bengal Chloramphenicol Agar (DRBC) (Kasvi, Pinhais, PR, Brazil) and Sabouraud Dextrose Agar (Kasvi, Pinhais, PR, Brazil) with chloramphenicol (100 mg/L) (ASC). The plates were opened 1 m above the cave floor for 20 minutes (Cunha et al. 2020). To isolate fungi from soil, 1 g of soil was suspended in 9 mL of distilled and sterilised water and shaken manually. Serial dilutions were then made up to 10^–4^. From the suspension diluted to 10^–4^, 1 mL was removed and transferred to the surface of the DRBC and ASC media in Petri dishes (Bills et al. 2004; Cunha et al. 2020), which were incubated in the dark at 25 °C for up to 14 days. After growth, the colonies were purified and transferred to ASC or Potato Dextrose Agar (Kasvi, Pinhais, PR, Brazil) (PDA) plates. After the incubation period, the number of colony-forming units (CFU) from the air and per 1 g of soil was calculated (Cunha et al. 2020). The fungal colonies were purified and transferred to plates containing PDA.

### DNA extraction, amplification, and sequencing

Isolates grown on PDA (Kasvi, Pinhais, PR, Brazil) (7–10 days at 25 °C in the dark) were used for genomic DNA extraction using the Wizard Genomic DNA Purification Kit (Promega Corporation, Madison, WI, USA), following the manufacturer’s protocol. ITS, including the 5.8S rDNA region, the actin gene (*ACT*), the translation elongation factor 1-alpha gene (*TEF1-α*), partial β-tubulin (*TUB*), and the second largest subunit of RNA polymerase II (*RPB2*) were amplified using the primer pairs ITS4 and ITS5 (White et al. 1990); ACT-512F and ACT-783R (Carbone and Kohn 1999); EF-728F and EF-986R (Carbone and Kohn 1999); T1 and Tub4RD (O’Donnell and Cigelnik 1997; Groenewald et al. 2013); Bt10 and Bt2b (Glass and Donaldson 1995; O’Donnell and Cigelnik 1997) or Bt2a and Bt2b (Glass and Donaldson 1995); and fRPB2-5F and fRPB2-7cR (Liu et al. 1999) or RPB2-5F2 and fRPB2-7cR (Liu et al. 1999; Sung et al. 2007), respectively. The PCR conditions were defined as described by Bensch et al. (2012) and Bezerra et al. (2017). The PCR products obtained were purified using the Exonuclease/Alkaline Phosphatase mix (Cellco Biotec, Brazil), according to the manufacturer’s instructions, and sent for sequencing with the same primers using the BigDye® Terminator v. 3.1 Cycle Sequencing Kit (Applied Biosystems Life Technologies, Carlsbad, CA, USA) at the Plataforma Multiusuários de Genômica e Transcriptômica do Centro de Biociências (MULTISEQ) of the Universidade Federal de Pernambuco (Recife, Brazil) and at the Centro Multiusuário de Pesquisa de Bioinsumos e Tecnologias em Saúde (CMBiotecs) of the Universidade Federal de Goiás (Goiás, Brazil). Sequences obtained in this study were deposited in GenBank (Table 1).

**Table 1.** List of species and sequences used in the phylogenetic analyses. “^T^” = ex-type strains. Species and sequences obtained in this study are shown in bold.

Species	Strains/Isolates	Hosts/Substrates	Collection sites	GenBank accession numbers					References
				*ACT*	ITS	*RPB2*	*TEF1-α*	*TUB*	
*Cladosporium cladosporioides* species complex									
*C. acalyphae*	CBS 125982^T^	*Acalypha australis*	South Korea	HM148481	HM147994	—	HM148235	—	Bensch et al. (2010)
*C. alboflavescens*	CBS 140690^T^	Bronchoalveolar lavage fluid of animal	USA	LN834604	LN834420	—	LN834516	—	Sandoval-Denis et al. (2016)
*C. angulosum*	CBS 140692^T^	Bronchoalveolar lavage fluid of human	USA	LN834609	LN834425	—	LN834521	—	Sandoval-Denis et al. (2016)
	CPC 18496	*Ananas comosus*	Panama	KT600610	KT600414	—	KT600512	—	Bensch et al. (2015)
	CPC 11526	*Acacia mangium*	Thailand	HM148616	HM148127	—	HM148371	—	Bensch et al. (2018)
	CPC 14566	*Corymbia foelscheana*	Australia	HM148636	HM148147	—	HM148391	—	Bensch et al. (2010)
	CPC 18494	*Ananas comosus*	Panama	KT600609	KT600413	—	KT600511	—	Bensch et al. (2015)
	FCCUFG 202	Air sample in a Cerrado cave	Brazil	PZ157678	—	—	—	—	This study
*C. angustisporum*	CBS 125983^T^	*Alloxylon wickhamii*	Australia	HM148482	HM147995	—	HM148236	PZ136721	Bensch et al. (2010) and This study
	UTHSC DI-13-240	Human, toe nail	USA	LN834540	LN834356	—	LN834452	—	Sandoval-Denis et al. (2015)
	CPC 22371	Indoor air sample, office	USA	MF473770	MF472920	—	MF473347	—	Bensch et al. (2018)
	DTO 127-E6	Air sample, bakery	USA	KP702057	KP701935	—	KP701812	—	Bensch et al. (2018)
	CPC 22345	Outside air sample	USA	MF473769	MF472919	PZ136677	MF473346	PZ136722	Bensch et al. (2018) and This study
*C. angustiterminale*	CBS 140480^T^	*Banksia grandis*	Australia	KT600575	KT600379	—	KT600476	—	Bensch et al. (2015)
*C. anthropophilum*	CBS 117483	Unknown	USA	HM148494	HM148007	—	HM148248	—	Sandoval-Denis et al. (2016)
	CBS 140685^T^	Bronchoalveolar lavage fluid of human	USA	LN834621	LN834437	—	LN834533	—	Sandoval-Denis et al. (2016)
*C. asperulatum*	CBS 126339	*Eucalyptus* leaf litter	India	HM148484	HM147997	—	HM148238	—	Bensch et al. (2010)
	CBS 126340^T^	*Protea susannae*	Portugal	HM148485	HM147998	—	HM148239	—	Bensch et al. (2010)
*C. aulonemiae*	COAD 2269^T^	Decayed leaf of *Aulonemia amplissima*	Brazil	MT373119	MZ318427	PZ136678	MT680198	PZ136723	Costa et al. (2022) and This study
	COAD 2270	Decayed leaf of *Aulonemia amplissima*	Brazil	MT373120	MZ318428	PZ136679	MT680199	PZ136724	Costa et al. (2022) and This study
	JB200	Air sample in a Cerrado cave	Brazil	PZ157679	PZ121157	—	—	—	This study
*C. australiense*	CBS 125984^T^	*Eucalyptus moluccana*	Australia	HM148486	HM147999	—	HM148240	—	Bensch et al. (2010)
*C. austroafricanum*	CBS 140481^T^	Leaf litter	New Zealand	KT600577	KT600381	—	KT600478	—	Bensch et al. (2015)
*C. bambusicola*	COAD 2256^T^	Decayed leaf of *Aulonemia amplissima*	Brazil	MT373125	MZ318433	PZ136680	MT680204	PZ136725	Costa et al. (2022) and This study
	COAD 2562	On uredinia of *Hemileia vastatrix* on leaves of *Coffea arabica*	Brazil	OP598123	OP535371	—	OP676082	—	Pereira et al. (2024)
	COAD 2565	On uredinia of *Hemileia vastatrix* on leaves of *Coffea arabica*	Brazil	OP598124	OP535372	—	OP676083	—	Pereira et al. (2024)
	COAD 2573	On uredinia of *Hemileia vastatrix* on leaves of *Coffea arabica*	Brazil	OP598121	OP535369	—	OP676080	—	Pereira et al. (2024)
	COAD 3516	On uredinia of *Hemileia vastatrix* on leaves of *Coffea arabica*	Brazil	OR669144	OR666147	—	OR669145	—	Pereira et al. (2024)
	GUCC 21244.1, ex-type of *C. ribis*	On leaves of *Ribes burejense*	China	OP863098	OP852666	—	OP859046	—	Yang et al. (2023)
	COAD 2550, *C. ribis*	On uredinia of *Hemileia vastatrix* on leaves of *Coffea arabica*	Brazil	OP598117	OP535365	PZ136681	OP676076	—	Pereira et al. (2024) and This study
	COAD 3116, ex-type of *C. speluncae*	Soil sample in Monte Cristo cave	Brazil	ON141934	ON062329	—	ON982818	—	Dutra et al. (2023)
	X13	Air sample in a bat cave	Brazil	MZ555741	MZ518823	—	MZ555728	—	Pereira et al. (2022)
	JB1511	Air sample in a Cerrado cave	Brazil	PZ157680	—	—	—	—	This study
	JB1545	Air sample in a Cerrado cave	Brazil	PZ157681	—	—	—	—	This study
	FCCUFG 129	Air sample in a Cerrado cave	Brazil	PZ157682	—	PZ136682	PZ157632	PZ136726	This study
	JB1534	Air sample in a Cerrado cave	Brazil	PZ157683	—	—	—	—	This study
	JB1504	Air sample in a Cerrado cave	Brazil	PZ157684	—	—	—	—	This study
	JB1552	Air sample in a Cerrado cave	Brazil	PZ157685	—	—	—	—	This study
	JB1529	Air sample in a Cerrado cave	Brazil	PZ157686	—	—	—	—	This study
	FCCUFG 120	Air sample in a Cerrado cave	Brazil	PZ157687	—	—	PZ157633	—	This study
	FCCUFG 139	Air sample in a Cerrado cave	Brazil	PZ157688	—	—	PZ157634	PZ136727	This study
	JB800	Soil sample in a Cerrado cave	Brazil	PZ157689	PZ121158	PZ136683	PZ157635	PZ136728	This study
	FCCUFG 138	Air sample in a Cerrado cave	Brazil	PZ157690	—	—	PZ157636	—	This study
	FCCUFG 114	Air sample in a Cerrado cave	Brazil	PZ157691	—	—	PZ157637	—	This study
	JB792	Air sample in a Cerrado cave	Brazil	PZ157692	PZ121159	PZ136684	PZ157638	PZ136729	This study
	FCCUFG 124	Air sample in a Cerrado cave	Brazil	PZ157693	—	—	PZ157639	—	This study
	JB2497	Soil sample in a Cerrado cave	Brazil	PZ157694	—	—	—	—	This study
	JB1458	Air sample in a Cerrado cave	Brazil	PZ157695	—	—	—	—	This study
	JB1502	Air sample in a Cerrado cave	Brazil	PZ157696	—	—	—	—	This study
	JB1507	Air sample in a Cerrado cave	Brazil	PZ157697	—	—	—	—	This study
	JB1547	Air sample in a Cerrado cave	Brazil	PZ157698	—	—	—	—	This study
	JB1524	Air sample in a Cerrado cave	Brazil	PZ157699	—	—	—	—	This study
	JB1544	Air sample in a Cerrado cave	Brazil	PZ157700	—	—	—	—	This study
	JB1964	Soil sample in a Cerrado cave	Brazil	PZ157701	—	—	—	—	This study
	JB2515	Soil sample in a Cerrado cave	Brazil	PZ157702	—	—	—	—	This study
	JB1526	Air sample in a Cerrado cave	Brazil	PZ157703	—	—	—	—	This study
	JB1457	Air sample in a Cerrado cave	Brazil	PZ157704	—	—	—	—	This study
	JB1516	Air sample in a Cerrado cave	Brazil	PZ157705	—	—	—	—	This study
	JB1492	Air sample in a Cerrado cave	Brazil	PZ157706	—	—	—	—	This study
	JB1495	Air sample in a Cerrado cave	Brazil	PZ157707	—	—	—	PZ136730	This study
	JB1496	Air sample in a Cerrado cave	Brazil	PZ157708	—	—	—	—	This study
*C. benschiae*	COAD 2263^T^	Decayed leaf of *Aulonemia amplissima*	Brazil	MT373128	MZ318436	—	MT680207	—	Costa et al. (2022)
	COAD 2265	Decayed leaf of *Aulonemia amplissima*	Brazil	MT373129	MZ318437	—	MT680208	—	Costa et al. (2022)
*C. camelliae*	YCW410^T^	*Camellia sinensis*	China	OP588395	OP558377	—	OP586704	—	Lv et al. (2023)
	YCW55	*Camellia sinensis*	China	OP588408	OP558390	—	OP586717	—	Lv et al. (2023)
*C. caprifimosum*	FMR 16532^T^	From goat dung	Spain	LR813205	LR813198	—	LR813210	—	Iturrieta-González et al. (2021)
*C. carsi* sp. nov.	URM 9208^T^ = FCCUFG 73	Air sample in a Cerrado cave	Brazil	PZ157720	PZ121170	PZ136686	PZ157647	PZ136733	This study
	FCCUFG 146	Air sample in a Cerrado cave	Brazil	PZ157721	PZ121171	PZ136687	PZ157648	PZ136734	This study
*C. cavernicola*	URM 8389^T^	Air sample in a bat cave	Brazil	MZ555746	MZ518829	—	MZ555733	—	Pereira et al. (2022)
*C. chalastosporoides*	CBS 125985^T^	Fruiting bodies of *Teratosphaeria proteae-arboreae* on leaves of *Protea nitida*	South Africa	HM148488	HM148001	—	HM148242	—	Bensch et al. (2010)
*C. chlamydosporiformans*	COAD 2561	On uredinia of *Hemileia vastatrix* on leaves of *Coffea arabica*	Brazil	OP598122	OP535370	—	OP676081	—	Pereira et al. (2024)
	COAD 2570	On uredinia of *Hemileia vastatrix* on leaves of *Coffea arabica*	Brazil	OP598116	OP535364	—	OP676075	PZ136731	Pereira et al. (2024) and This study
	COAD 2571^T^	On uredinia of *Hemileia vastatrix* on leaves of *Coffea arabica*	Kenya	OP598126	OP535374	PZ136685	OP676085	PZ136732	Pereira et al. (2024) and This study
	COAD 2568	On uredinia of *Hemileia vastatrix* on leaves of *Coffea arabica*	Ethiopia	OP598130	OP535378	—	OP676089	—	Pereira et al. (2024)
	FCCUFG 108	Air sample in a Cerrado cave	Brazil	PZ157709	PZ121160	—	—	—	This study
	FCCUFG 77	Air sample in a Cerrado cave	Brazil	PZ157710	PZ121161	—	PZ157640	—	This study
	FCCUFG 133	Air sample in a Cerrado cave	Brazil	PZ157711	PZ121162	—	—	—	This study
	FCCUFG 75	Air sample in a Cerrado cave	Brazil	PZ157712	PZ121163	—	PZ157641	—	This study
	FCCUFG 78	Air sample in a Cerrado cave	Brazil	PZ157713	PZ121164	—	PZ157642	—	This study
	FCCUFG 130	Air sample in a Cerrado cave	Brazil	PZ157714	PZ121165	—	PZ157643	—	This study
	FCCUFG 74	Air sample in a Cerrado cave	Brazil	PZ157715	PZ121166	—	PZ157644	—	This study
	FCCUFG 115	Air sample in a Cerrado cave	Brazil	PZ157716	PZ121167	—	—	—	This study
	FCCUFG 68	Air sample in a Cerrado cave	Brazil	PZ157717	PZ121168	—	PZ157645	—	This study
	FCCUFG 70	Air sample in a Cerrado cave	Brazil	PZ157718	PZ121169	—	PZ157646	—	This study
*C. chubutense*	CBS 124457^T^	*Pinus ponderosa*	Argentina	FJ936165	FJ936158	—	FJ936161	—	Bensch et al. (2010)
*C. chusqueae*	COAD 2258^T^	Decayed leaf of *Chusquea urelytra*	Brazil	MT373122	MZ318430	—	MT680201	—	Costa et al. (2022)
	COAD 2261	Decayed leaf of *Chusquea urelytra*	Brazil	MT373124	MZ318431	—	MT680203	—	Costa et al. (2022)
*C. cladosporioides*	CBS 113738	Grape bud	USA	HM148491	HM148004	—	HM148245	—	Bensch et al. (2010)
	CBS 112388^T^	*Pisum sativum*	Germany	HM148490	HM148003	KX288432	HM148244	—	Bensch et al. (2010)
*C. colocasiae*	CBS 386.64^T^	*Colocasia esculenta*	Taiwan	HM148555	HM148067	—	HM148310	—	Bensch et al. (2010)
	CBS 119542	*Colocasia esculenta*	Japan	HM148554	HM148066	—	HM148309	—	Bensch et al. (2010)
*C. compactisporum*	AUMC 11366^T^	Air sample	Egypt	OL514010	MN826822	—	—	—	Moharram et al. (2022)
*C. congjiangense*	GUCC 21208.3^T^	On leaves of *Passiflora edulis*	China	OP863094	OP852675	—	OP859042	—	Yang et al. (2023)
	GUCC 21208.5	On leaves of *Passiflora edulis*	China	OP863095	OP852676	—	OP859043	—	Yang et al. (2023)
*C. coprophilum*	FMR 16101^T^	From unidentified herbivore dung	Spain	LR813204	LR813199	—	LR813211	—	Iturrieta-González et al. (2021)
*C. crousii*	CBS 140686^T^	Bronchoalveolar lavage fluid of human	USA	LN834615	LN834431	—	LN834527	—	Sandoval-Denis et al. (2016)
*C. cucumerinum*	CBS 171.52^T^	*Cucumis sativus*	Netherlands	HM148561	HM148072	—	HM148316	MZ073923	Bensch et al. (2010)
	CBS 173.54	*Cucumis sativus*	Netherlands	HM148563	HM148074	—	HM148318	—	Bensch et al. (2010)
*C. delicatulum*	CBS 126342	Indoor air	Denmark	HM148568	HM148079	—	HM148323	—	Bensch et al. (2010)
	CBS 126344^T^	*Tilia cordata*	Germany	HM148570	HM148081	—	HM148325	—	Bensch et al. (2010)
*C. devikae*	BRIP 72278a^T^	From flower blight of *Macadamia integrifolia*	Australia	MZ344212	MZ303808	—	MZ344193	—	Prasannath et al. (2021)
*C. diamantinense*	COAD 3108^T^	Air sample in Monte Cristo cave	Brazil	ON141933	ON062328	—	ON982817	—	Dutra et al. (2023)
*C. eucommiae*	GUCC 401.1^T^	Fallen leaves of *Eucommia ulmoides*	China	OL519775	OL587465	—	OL504966	—	Wang et al. (2022)
	GUCC 401.9	Fallen leaves of *Eucommia ulmoides*	China	ON383337	ON334729	—	—	—	Wang et al. (2022)
*C. europaeum*	CBS 116744	*Acer pseudoplatanus*	Germany	HM148540	HM148053	—	HM148294	—	Bensch et al. (2018)
*C. exasperatum*	CBS 125986^T^	*Eucalyptus tintinnans*	Australia	HM148579	HM148090	—	HM148334	—	Bensch et al. (2010)
*C. exile*	CBS 125987^T^	Chasmothecia of *Phyllactinia guttata* on leaves of *Corylus avellana*	USA	HM148580	HM148091	—	HM148335	—	Bensch et al. (2010)
*C. flabelliforme*	CBS 126345^T^	*Melaleuca cajuputi*	Australia	HM148581	HM148092	—	HM148336	—	Bensch et al. (2010)
	FCCUFG 201	Air sample in a Cerrado cave	Brazil	PZ157719	—	—	—	—	This study
*C. flavovirens*	CBS 140462^T^	Man, toenail	USA	LN834624	LN834440	—	LN834536	—	Sandoval-Denis et al. (2016)
*C. funiculosum*	CBS 122128	*Ficus carica*	Japan	HM148582	HM148093	—	HM148337	—	Bensch et al. (2010)
	CBS 122129^T^	*Vigna umbellata*	Japan	HM148583	HM148094	—	HM148338	—	Bensch et al. (2010)
*C. fuscoviride*	FMR 16385^T^	From garden soil	Spain	LR813206	LR813200	—	LR813212	—	Iturrieta-González et al. (2021)
*C. gamsianum*	CBS 125989^T^	*Strelitzia* sp.	South Africa	HM148584	HM148095	—	HM148339	—	Bensch et al. (2010)
*C. globisporum*	CBS 812.96^T^	Meat stamp	Sweden	HM148585	HM148096	—	HM148340	—	Bensch et al. (2010)
*C. grevilleae*	CBS 114271^T^	*Grevillea* sp.	Australia	JF770473	JF770450	—	JF770472	—	Crous et al. (2011)
*C. guizhouense*	GUCC 401.7^T^	Fallen leaves of *Eucommia ulmoides*	China	OL519780	OL579741	—	OL504965	—	Wang et al. (2022)
	GUCC 401.8	Fallen leaves of *Eucommia ulmoides*	China	ON383338	ON334728	—	ON383470	—	Wang et al. (2022)
*C. hemileiicola*	COAD 2567^T^	On uredinia of *Hemileia vastatrix* on leaves of *Coffea arabica*	Brazil	OP598128	OP535376	—	OP676087	—	Pereira et al. (2024)
	COAD 3350	On uredinia of *Hemileia vastatrix* on leaves of *Coffea arabica*	Ethiopia	OP598133	OP535381	—	OP676092	—	Pereira et al. (2024)
*C. hillianum*	CBS 125988^T^	Leaf mold of *Typha orientalis*	New Zealand	HM148586	HM148097	—	HM148341	—	Bensch et al. (2010)
*C. inversicolor*	CBS 143.65	Leaf of *Tilia* sp.	Netherlands	HM148589	HM148100	—	HM148344	—	Bensch et al. (2010)
	CBS 401.80^T^	Leaf of *Triticum aestivum*	Netherlands	HM148590	HM148101	—	HM148345	—	Bensch et al. (2010)
*C. ipereniae*	CBS 140483^T^	*Puya* sp.	Chile	KT600589	KT600394	—	KT600491	—	Bensch et al. (2015)
	CPC 16855	*Arctosta phylospallida*	USA	KT600590	KT600395	—	KT600492	—	Bensch et al. (2015)
*C. iranicum*	CBS 126346^T^	Leaf of *Citrus sinensis*	Iran	HM148599	HM148110	—	HM148354	—	Bensch et al. (2010)
*C. kaiyangense*	GUCC 21265.2^T^	On decaying fruit of *Eriobotrya japonica*	China	OP859045	OP852665	—	OP863097	—	Yang et al. (2023)
*C. lacerdae* sp. nov.	URM 9210^T^ = FCCUFG 72	Air sample in a Cerrado cave	Brazil	PZ157722	PZ121172	PZ136688	PZ157649	PZ136735	This study
	FCCUFG 122	Air sample in a Cerrado cave	Brazil	PZ157723	PZ121173	PZ136689	PZ157650	PZ136736	This study
*C. lagenariiforme*	SFC20230103-M22	Seaweed	Republic of Korea	OQ185166	OQ186118	—	OQ185127	—	Lee et al. (2023)
	SFC20230103-M23^T^	Seaweed	Republic of Korea	OQ185167	OQ186119	—	OQ185128	—	Lee et al. (2023)
*C. lentulum*	FMR 16288^T^	From unidentified leaf litter	Spain	LR813209	LR813203	—	LR813215	—	Iturrieta-González et al. (2021)
*C. licheniphilum*	CBS 125990^T^	*Phaeophyscia orbicularis* and *Physcia* sp.	Germany	HM148600	HM148111	—	HM148355	—	Bensch et al. (2010)
*C. longicatenatum*	CBS 140485^T^	Unknown plant	Australia	KT600598	KT600403	—	KT600500	—	Bensch et al. (2015)
*C. lycoperdinum*	CBS 574.78C	*Aureobasidium caulivorum*	Russia	HM148604	HM148115	—	HM148359	—	Bensch et al. (2010)
	CBS 126347	Galls of *Apiosporina morbosa* on *Prunus* sp.	Canada	HM148601	HM148112	—	HM148356	—	Bensch et al. (2010)
*C. mambaiense* sp. nov.	URM 9104^T^ = FCCUFG 71	Air sample in a Cerrado cave	Brazil	PZ157724	PZ121174	PZ136690	PZ157651	PZ136737	This study
	FCCUFG 147	Air sample in a Cerrado cave	Brazil	PZ157725	PZ121175	PZ136691	PZ157652	PZ136738	This study
*C. macadamiae*	BRIP 72269a^T^	From flower blight of *Macadamia integrifolia*	Australia	MZ344214	MZ303810	—	MZ344195	—	Prasannath et al. (2021)
	BRIP 72287a	From flower blight of *Macadamia integrifolia*	Australia	MZ344215	MZ303811	—	MZ344196	—	Prasannath et al. (2021)
	COAD 3373	*Austropuccinia psidii pustules* on leaf of *Syzygium jambos*	Brazil	OQ605785	—	PZ136692	OQ605776	PZ136739	Silva et al. (2023) and This study
	FCCUFG 116	Air sample in a Cerrado cave	Brazil	PZ157726	PZ121176	PZ136693	PZ157653	PZ136740	This study
	FCCUFG 135	Air sample in a Cerrado cave	Brazil	PZ157727	PZ121177	PZ136694	PZ157654	PZ136741	This study
	FCCUFG 67	Soil sample in a Cerrado cave	Brazil	PZ157728	PZ121178	—	PZ157655	PZ136742	This study
*C. maltirimosum*	SFC20230103-M51^T^	Sediment	Republic of Korea	OQ185195	OQ186147	—	OQ185155	—	Lee et al. (2023)
	SFC20230103-M52	Sediment	Republic of Korea	OQ185196	OQ186148	—	OQ185156	—	Lee et al. (2023)
*C. marinum*	SFC20230103-M30	Seaweed	Republic of Korea	OQ185174	OQ186126	—	OQ185135	—	Lee et al. (2023)
	SFC20230103-M33^T^	Seaweed	Republic of Korea	OQ185177	OQ186129	—	OQ185137	—	Lee et al. (2023)
*C. montecillanum*	CBS 140486^T^	*Pine needles*	Mexico	KT600602	KT600406	—	KT600504	—	Bensch et al. (2015)
	CPC 15605	*Taraxacum* sp.	Mexico	KT600603	KT600407	—	KT600505	—	Bensch et al. (2015)
*C. myrtacearum*	CBS 126350^T^	*Corymbia foelscheana*	Australia	HM148606	HM148117	—	HM148361	—	Bensch et al. (2010)
*C. nayongense*	GUCC 21260.3^T^	Leaves of *Prunus pseudocerasus*	China	OP859054	OP852669	—	OP863106	—	Yang et al. (2023)
*C. neapolitanum*	MgPo1^T^	From receptacle of *Micromeria graeca* flower	Italy	MK416051	MK387890	—	MK416094	—	Zimowska et al. (2021)
*C. needhamense*	CBS 143359^T^	Indoor air sample	USA	MF473991	MF473142	—	MF473570	—	Bensch et al. (2018)
*C. neerlandicum*	CBS 143360^T^	Swab sample	Netherlands	KP702010	KP701887	—	KP701764	—	Bensch et al. (2018)
*C. neopsychrotolerans*	CGMCC 3.18031^T^	From the rhizosphere soil of *Saussurea involucrata*	China	KX938366	KX938383	—	KX938400	—	Ma et al. (2017)
*C. nogueirae* sp. nov.	FCCUFG 125	Air sample in a Cerrado cave	Brazil	PZ157729	PZ121179	PZ136695	PZ157656	PZ136743	This study
	FCCUFG 137	Air sample in a Cerrado cave	Brazil	PZ157730	PZ121180	PZ136696	PZ157657	PZ136744	This study
	URM 9209^T^ = FCCUFG 61	Air sample in a Cerrado cave	Brazil	PZ157731	PZ121181	PZ136697	PZ157658	PZ136745	This study
*C. oxysporum*	CBS 125991^T^	Soil, near the terracotta army	China	HM148607	HM148118	PZ136698	HM148362	PZ136746	Bensch et al. (2010) and This study
	CBS 126351	Indoor air	Venezuela	HM148608	HM148119	PZ136699	HM148363	PZ136747	Bensch et al. (2010) and This study
*C. paracladosporioides*	CBS 171.54^T^	—	—	HM148609	HM148120	—	HM148364	—	Bensch et al. (2010)
*C. parapenidielloides*	CBS 140487^T^	*Eucalyptus* sp.	Australia	KT600606	KT600410	—	KT600508	—	Bensch et al. (2015)
*C. passiflorae*	COAD 2135^T^	Leaves of *Passiflora edulis*	Brazil	MH729795	MH682175	—	MH724943	—	Rosado et al. (2019)
	COAD 2136	Leaves of *Passiflora edulis*	Brazil	MH729796	MH682176	—	MH724944	—	Rosado et al. (2019)
*C. passifloricola*	COAD 2140^T^	Leaves of *Passiflora edulis*	Brazil	MH729800	—	—	MH724948	—	Rosado et al. (2019)
*C. perangustum*	CBS 125996^T^	*Cussonia* sp.	South Africa	HM148610	HM148121	—	HM148365	—	Bensch et al. (2010)
	CBS 126365	Chasmothecia of *Phyllactinia guttata* on leaves of *Corylus avellana*	USA	HM148612	HM148123	—	HM148367	—	Bensch et al. (2010)
	FCCUFG 200	Air sample in a Cerrado cave	Brazil	PZ157732	—	—	—	—	This study
*C. pernambucoense*	URM 8390^T^	Air sample in a bat cave	Brazil	MZ555745	MZ518828	—	MZ555732	—	Pereira et al. (2022)
	URM 8391	Air sample in a bat cave	Brazil	MZ555747	MZ518830	PZ136700	MZ555734	—	Pereira et al. (2022) and This study
	JB791	Soil sample in a Cerrado cave	Brazil	PZ157733	—	—	—	—	This study
*C. phaenocomae*	CBS 128769^T^	Leaf bracts of *Phaenocoma prolifera*	South Africa	JF499881	JF499837	—	JF499875	—	Crous et al. (2011)
*C. phyllactiniicola*	CBS 126353	Chasmothecia of *Phyllactinia guttata* on leaves of *Corylus avellana*	USA	HM148640	HM148151	—	HM148395	—	Bensch et al. (2010)
	CBS 126355^T^	Chasmothecia of *Phyllactinia guttata* on leaves of *Corylus avellana*	USA	HM148642	HM148153	—	HM148397	—	Bensch et al. (2010)
*C. phyllophilum*	CBS 125992^T^	*Taphrina* sp. on *Prunus cerasus*	Germany	HM148643	HM148154	—	HM148398	—	Bensch et al. (2010)
	CPC 13873	On *Teratosphaeria proteae-arboreae* on *Protea arborea*	South Africa	HM148644	HM148155	—	HM148399	—	Bensch et al. (2010)
*C. pini-ponderosae*	CBS 124456^T^	*Pinus ponderosa*	Argentina	FJ936167	FJ936160	—	FJ936164	—	Bensch et al. (2010)
*C. polonicum*	Th/lg/2334^T^	From gall of *Asphondylia serpylli* on *Thymus vulgaris*	Poland	MK416055	MK387894	—	MK416098	—	Zimowska et al. (2021)
*C. propiciense* sp. nov.	URM 9353^T^ = FCCUFG 76	Air sample in a Cerrado cave	Brazil	PZ157734	PZ121182	PZ136701	PZ157659	PZ136748	This study
	FCCUFG 123	Air sample in a Cerrado cave	Brazil	PZ157735	PZ121183	PZ136702	PZ157660	PZ136749	This study
*C. proteacearum*	BRIP 72301a^T^	From flower blight of *Macadamia integrifolia*	Australia	MZ344213	MZ303809	—	MZ344194	—	Prasannath et al. (2021)
	SFC20230103-M53	Seaweed	Republic of Korea	OQ185002	OQ165252	—	OQ185084	—	Lee et al. (2023)
	SFC20230103-M54	Sediment	Republic of Korea	OQ185003	OQ165253	—	OQ185085	—	Lee et al. (2023)
	SFC20230103-M55	Sediment	Republic of Korea	OQ185004	OQ165254	—	OQ185086	—	Lee et al. (2023)
*C. pruni-salicinae*	GUCC 21206.1^T^	On leaves of *Prunus salicina*	China	OP863092	OP852683	—	OP859041	—	Yang et al. (2023)
	GUCC 21266.1	On leaves of *Prunus salicina*	China	OP863093	OP852684	—	—	—	Yang et al. (2023)
	FCCUFG 198	Air sample in a Cerrado cave	Brazil	PZ157736	—	—	—	—	This study
*C. pseudochalastosporoides*	CBS 140490^T^	Pine needles	Mexico	KT600611	KT600415	—	KT600513	—	Bensch et al. (2015)
*C. pseudocladosporioides*	CBS 667.80	*Malus sylvestris*	Italy	HM148654	HM148165	—	HM148409	—	Bensch et al. (2010)
	CBS 125993^T^	Outside air	Netherlands	HM148647	HM148158	—	HM148402	—	Bensch et al. (2010)
	FCCUFG 203	Air sample in a Cerrado cave	Brazil	PZ157737	—	—	—	—	This study
	FCCUFG 199	Air sample in a Cerrado cave	Brazil	PZ157738	—	—	—	—	This study
*C. pseudotenuissimum*	COAD 2266^T^	Decayed leaf of *Chusquea urelytra*	Brazil	MT373132	MZ318439	PZ136703	MT680211	PZ136750	Costa et al. (2022) and This study
	COAD 2259	Decayed leaf of *Chusquea anelytroides*	Brazil	MT373130	MZ318438	PZ136704	MT680209	PZ136751	Costa et al. (2022) and This study
	COAD 2268	Decayed leaf of *Chusquea urelytra*	Brazil	MT373134	MZ318441	—	MT680213	—	Costa et al. (2022)
*C. punicae*	GUCC 21271.5^T^	On leaves of *Punica granatum*	China	OP863108	OP852672	—	OP859056	—	Yang et al. (2023)
*C. puris*	COAD 2487^T^	Submerged litter in streams	Brazil	MK249980	MK253337	PZ136705	MK293777	PZ136752	Freitas et al. (2021) and This study
	COAD 2257, ex-type of *C. brigadeirense*	Decayed leaf of *Chusquea urelytra*	Brazil	MT373127	MZ318435	PZ136706	MT680206	PZ136753	Costa et al. (2022) and This study
	COAD 2566	On uredinia of *Hemileia vastatrix* on leaves of *Coffea arabica*	Brazil	OP598125	OP535373	PZ136707	OP676084	—	Pereira et al. (2024) and This study
	COAD 2572	On uredinia of *Hemileia vastatrix* on leaves of *Coffea arabica*	Brazil	OP598127	OP535375	PZ136708	OP676086	—	Pereira et al. (2024) and This study
	FCCUFG 145	Air sample in a Cerrado cave	Brazil	PZ157739	—	PZ136709	PZ157661	PZ136754	This study
*C. rectoides C. rugulovarians C. scabrellum C. setoides*	CBS 125994^T^	*Vitis flexuosa*	South Korea	HM148683	HM148193	—	HM148438	—	Bensch et al. (2010)
	CBS 140495^T^	Leaf sheaths of unidentified *Poaceae*	Brazil	KT600656	KT600459	PZ136710	KT600558	PZ136755	Bensch et al. (2015) and This study
	CBS 126358^T^	*Ruscus hypoglossum*	Slovenia	HM148685	HM148195	—	HM148440	—	Bensch et al. (2010)
	COAD 2576	On uredinia of *Hemileia vastatrix* on leaves of *Coffea arabica*	Ethiopia	OP598132	OP535380	PZ136711	OP676091	PZ136756	Pereira et al. (2024) and This study
	COAD 3470^T^	On uredinia of *Hemileia vastatrix* on leaves of *Coffea arabica*	Ethiopia	OP598131	OP535379	PZ136712	OP676090	PZ136757	Pereira et al. (2024) and This study
*C. silenes*	CBS 109082^T^	Stems of *Silene maritima*	England	EF679506	EF679354	—	EF679429	—	Crous et al. (2011)
*C. sinuatum*	CGMCC 3.18096^T^	From alpine soil	China	KX938368	KX938385	—	KX938402	—	Ma et al. (2017)
	CGMCC 3.18097	From alpine soil	China	KX938369	KX938386	—	KX938403	—	Ma et al. (2017)
*C. subuliforme*	CBS 126500^T^	*Chamaedorea metallica*	Thailand	HM148686	HM148196	PZ136713	HM148441	—	Bensch et al. (2010) and This study
	UTHSC DI-13-214	Bronchoalveolar lavage fluid of human	USA	LN834578	LN834394	—	LN834490	—	Sandoval-Denis et al. (2015)
	CPC 15833	*Citrus* sp.	Mexico	KT600650	KT600453	—	KT600552	—	Bensch et al. (2015)
	CPC 18243	Cotton (*Gossypium* sp.), leaves	Brazil	KT600653	KT600456	—	KT600555	—	Bensch et al. (2015)
	CPC 15838	*Agave tequilana var. azul*	Mexico	KT600651	KT600454	—	KT600553	—	Bensch et al. (2015)
	JB1530	Air sample in a Cerrado cave	Brazil	PZ157740	—	—	—	—	This study
	JB1569	Air sample in a Cerrado cave	Brazil	PZ157741	—	—	—	—	This study
	JB1540	Air sample in a Cerrado cave	Brazil	PZ157742	—	—	—	—	This study
	FCCUFG 127	Air sample in a Cerrado cave	Brazil	PZ157743	—	—	PZ157662	—	This study
	FCCUFG 119	Air sample in a Cerrado cave	Brazil	PZ157744	—	—	PZ157663	—	This study
	FCCUFG 110	Air sample in a Cerrado cave	Brazil	PZ157745	—	—	PZ157664	PZ136758	This study
	FCCUFG 121	Air sample in a Cerrado cave	Brazil	PZ157746	—	—	PZ157665	PZ136759	This study
	JB794	Air sample in a Cerrado cave	Brazil	PZ157747	—	—	—	—	This study
	JB813	Air sample in a Cerrado cave	Brazil	PZ157748	—	—	—	—	This study
	JB802	Air sample in a Cerrado cave	Brazil	PZ157749	—	—	—	—	This study
	JB1497	Air sample in a Cerrado cave	Brazil	PZ157750	—	—	—	—	This study
*C. tenuissimum*	CBS 125995^T^	*Lagerstroemia* sp.	USA	HM148687	HM148197	—	HM148442	—	Bensch et al. (2010)
	UTHSC DI-13-258	Human, thoracentesis fluid	USA	LN834588	LN834404	—	LN834500	—	Sandoval-Denis et al. (2015)
*C. tianshanense*	CGMCC 3.18033^T^	From the rhizosphere soil of *Saussurea involucrata*	China	KX938364	KX938381	—	KX938398	—	Ma et al. (2017)
	CGMCC 3.18034	From the rhizosphere soil of *Saussurea involucrata*	China	KX938365	KX938382	—	KX938399	—	Ma et al. (2017)
*C. uredinicola*	CPC 5390	On *Cronartium fusiforme f. sp. quercum* on *Quercus nigra* leaves	—	HM148712	AY251071	—	HM148467	—	Bensch et al. (2010)
*C. uwebraunianum*	CBS 143365^T^	Indoor air sample	Netherlands	MF474156	MF473306	—	MF473729	—	Bensch et al. (2018)
	DTO 305-H9	House dust	New Zealand	MF474157	MF473307	—	MF473730	—	Bensch et al. (2018)
*C. varians*	CBS 126362^T^	*Catalpa bungei*	Russia	HM148715	HM148224	—	HM148470	—	Bensch et al. (2010)
*C. verrucocladosporioides*	CBS 126363^T^	*Rhus chinensis*	South Korea	HM148717	HM148226	—	HM148472	—	Bensch et al. (2010)
*C. vicinum*	CBS 143366^T^	Indoor air sample	USA	MF474161	MF473311	—	MF473734	—	Bensch et al. (2018)
	CBS 306.84	Urediniospores of *Puccinia allii*	England	HM148544	HM148057	—	HM148299	—	Bensch et al. (2018)
*C. vignae*	CBS 121.25	*Vigna unguiculata*	USA	HM148718	HM148227	—	HM148473	—	Bensch et al. (2010)
*C. wenganense*	GUCC 21220.1^T^	On leaves of *Prunus persica*	China	OP863101	OP852682	—	OP859049	—	Yang et al. (2023)
	FCCUFG 117	Air sample in a Cerrado cave	Brazil	PZ157751	PZ121184	—	PZ157666	—	This study
	JB1541	Air sample in a Cerrado cave	Brazil	PZ157752	—	—	—	—	This study
*C. westerdijkiae*	CBS 113746^T^	Bing cherry fruits	USA	HM148548	HM148061	—	HM148303	—	Bensch et al. (2018)
	CPC 10150	*Fatoua villosa*	South Korea	HM148549	HM148062	—	HM148304	—	Bensch et al. (2018)
*C. xanthochromaticum*	CBS 140691^T^	Bronchoalveolar lavage fluid of human	USA	LN834599	LN834415	—	LN834511	—	Sandoval-Denis et al. (2016)
	CPC 11806	*Strelitzia* sp.	South Africa	HM148618	HM148129	—	HM148373	—	Sandoval-Denis et al. (2016)
	COAD 2559	On uredinia of *Hemileia vastatrix* on leaves of *Coffea arabica*	Brazil	OP598120	OP535368	—	OP676079	—	Pereira et al. (2024)
	CBS 126364	*Erythrophleum chlorostachys*	Australia	HM148611	HM148122	—	HM148366	—	Sandoval-Denis et al. (2016)
	CPC 12792	*Musa* sp.	Polynesia	HM148625	HM148136	—	HM148380	—	Bensch et al. (2018)
	CPC 11133	*Eucalyptus* sp.	India	HM148615	HM148126	—	HM148370	—	Bensch et al. (2018)
	CPC 11609	*Musa* sp.	India	EF679508	EF679356	—	EF679431	—	Bensch et al. (2018)
	CPC 11856	*Acacia mangium*	Thailand	HM148623	HM148134	—	HM148378	—	Bensch et al. (2018)
	JB799	Air sample in a Cerrado cave	Brazil	PZ157753	PZ121185	—	PZ157667	—	This study
	JB1456	Air sample in a Cerrado cave	Brazil	PZ157754	—	—	—	—	This study
	JB1499	Air sample in a Cerrado cave	Brazil	PZ157755	—	—	—	—	This study
*C. xylophilum*	CBS 125997^T^	*Picea abies*	Russia	HM148721	HM148230	—	HM148476	—	Bensch et al. (2010)
*C. yunnanense*	KUN HKAS 121704^T^	On leaves of *Paris polyphylla*	China	OL466937	OK338502	—	OL825680	—	Xu et al. (2021)
*Cladosporium sphaerospermum* species complex									
*C. aciculare*	CBS 140488^T^	*Syzygium corynanthum*	Australia	KT600607	KT600411	PZ136714	KT600509	PZ136760	Bensch et al. (2015) and This study
	FCCUFG 64	Air sample in a Cerrado cave	Brazil	PZ157756	PZ121186	PZ136715	PZ157668	—	This study
*C. aphidis*	CBS 132182^T^	On dead carcasses of aphids	Germany	JN906997	JN906978	—	JN906984	—	Bensch et al.2012
*C. austrohemisphaericum*	CBS 140482^T^	*Lagunaria patersonia*, black mould on fruit surface	New Zealand	KT600578	KT600382	—	KT600479	—	Bensch et al. (2015)
	DTO 305-E8	House dust	New Zealand	MF473785	MF472935	—	MF473362	—	Bensch et al. (2018)
	COAD 3351	On uredinia of *Hemileia vastatrix* on leaves of *Coffea arabica*	Ethiopia	OP598137	OP535385	—	OP676096	—	Pereira et al. (2024)
	COAD 3487	On uredinia of *Hemileia vastatrix* on leaves of *Coffea arabica*	Ethiopia	OP598129	OP535377	—	OP676088	—	Pereira et al. (2024)
	CPC 16250	*Cussonia thyrsiflora*	South Africa	—	KT600383	—	KT600480	—	Bensch et al. (2018)
*C. coloradense*	CBS 143357^T^	Indoor air sample	USA	MF473795	MF472945	—	MF473372	—	Bensch et al. (2018)
*C. cycadicola*	CBS 137970^T^	Leaves of *Cycas media*	Australia	KJ869227	KJ869122	—	KJ869236	—	Bensch et al. (2018)
*C. domesticum*	CBS 143358^T^	Indoor air sample	USA	MF473805	MF472955	—	MF473382	—	Bensch et al. (2018)
*C. dominicanum*	CBS 119415^T^	Hypersaline water, salt lake	Dominican Republic	EF101368	DQ780353	—	JN906986	—	Bensch et al. (2018)
*C. fusiforme*	CBS 119414^T^	Hypersaline water, saltern	Slovenia	EF101372	DQ780388	—	JN906988	—	Bensch et al. (2018)
	EXF-397	Hypersaline water, saltern	Slovenia	EF101373	DQ780389	—	KJ596595	—	Bensch et al. (2018)
*C. halotolerans*	CBS 119416^T^	Hypersaline water, salterns	Namibia	EF101397	DQ780364	—	JN906989	—	Bensch et al. (2018)
	UTHSC DI-13-250	Human, scalp	USA	LN834558	LN834374	—	LN834470	—	Sandoval-Denis et al. (2015)
	CPC 22335	Indoor air	USA	MF473841	MF472992	—	MF473420	—	Bensch et al. (2018)
*C. langeronii*	CBS 189.54^T^	Man, mycosis	Brazil	EF101357	DQ780379	—	JN906990	—	Bensch et al. (2018)
*C. lebrasiae*	CBS 138283^T^	Milk bread	France	KJ596631	KJ596568	—	KJ596583	—	Bensch et al. (2018)
*C. longissimum*	CBS 300.96^T^	Soil along coral reef coast	New Guinea	EF101385	DQ780352	PZ136716	EU570259	PZ136761	Bensch et al. (2015) and This study
*C. mesquitapaivae* sp. nov.	URM 9103^T^ = FCCUFG 65	Air sample in a Cerrado cave	Brazil	PZ157757	PZ121187	—	PZ157669	PZ136762	This study
	FCCUFG 136	Air sample in a Cerrado cave	Brazil	PZ157758	PZ121188	—	PZ157670	PZ136763	This study
*C. mucilaginosum*	COAD 2580	On uredinia of *Hemileia vastatrix* on leaves of *Coffea arabica*	Ethiopia	OP598136	OP535384	—	OP676095	—	Pereira et al. (2024)
	COAD 2581^T^	On uredinia of *Hemileia vastatrix* on leaves of *Coffea arabica*	Ethiopia	OP598138	OP535386	—	OP676097	—	Pereira et al. (2024)
*C. neolangeronii*	CBS 109868	Mortar of Muro Farnesiano	Italy	EF101362	DQ780377	—	MF473571	—	Bensch et al. (2018)
	CBS 797.97^T^	Indoor environment	Netherland	MF473992	MF473143	—	—	—	Bensch et al. (2018)
*C. parahalotolerans*	CBS 139585^T^	Swab sample	Netherland	KP702077	KP701955	—	KP701832	—	Bensch et al. (2018)
	CPC 22336	Indoor air sample	USA	MF474000	MF473152	—	MF473578	—	Bensch et al. (2018)
*C. parasphaerospermum*	AUMC 10865^T^	Air sample	Egypt	OL514008	MN826828	—	—	—	Moharram et al. (2022)
*C. penidielloides*	CBS 140489^T^	*Acacia verticillata*	Australia	KT600608	KT600412	—	KT600510	—	Bensch et al. (2015)
*C. psychrotolerans*	CBS 119412^T^	Hypersaline water	Slovenia	EF101365	DQ780386	—	JN906992	—	Bensch et al. (2018)
	DTO 305-G3	House dust	Australia	MF474072	MF473223	—	MF473645	—	Bensch et al. (2018)
*C. pulvericola*	CBS 109788	Indoor air	Canada	MF474074	MF473225	—	MF473647	—	Bensch et al. (2018)
	CBS 143362^T^	House dust	New Zealand	MF474075	MF473226	—	MF473648	—	Bensch et al. (2018)
*C. ruguloflabelliforme*	CBS 140494^T^	*Diatrapaceae* sp. on *Aloe* sp.	South Africa	KT600655	KT600458	—	KT600557	—	Bensch et al. (2015)
*C. salinae*	CBS 119413^T^	Hypersaline water, saltern	Slovenia	EF101390	DQ780374	—	JN906993	—	Bensch et al. (2018)
*C. sloanii*	CBS 143364^T^	Swab sample	Netherlands	MF474103	MF473253	—	MF473676	—	Bensch et al. (2018)
*C. sphaerospermum*	CBS 193.54^T^	Man, nails	Netherlands	EU570269	DQ780343	PZ136717	EU570261	—	Bensch et al. (2018) and This study
	CBS 117728	Wood	USA	EU570275	AF393709	—	EU570268	—	Bensch et al. (2018)
	CBS 109.14	Leaf of *Carya illinoensis*	USA	EF101384	DQ780350	—	EU570260	—	Bensch et al. (2018)
	UTHSC DI-13-237	Bronchoalveolar lavage fluid of human	USA	LN834574	LN834390	—	LN834486	—	Sandoval-Denis et al. (2015)
	COAD 2578	On uredinia of *Hemileia vastatrix* on leaves of *Coffea arabica*	Ethiopia	OP598135	OP535383	—	OP676094	—	Pereira et al. (2024)
	MABIK FU00001143, *C. marinisedimentum*	Sediment	Republic of Korea	OQ185171	OQ186123	—	OQ185132	—	Lee et al. (2023)
	SFC20230103-M28, ex-type of *C. marinisedimentum*	Sediment	Republic of Korea	OQ185172	OQ186124	—	OQ185133	—	Lee et al. (2023)
	SFC20230103-M29, *C. marinisedimentum*	Sediment	Republic of Korea	OQ185173	OQ186125	—	OQ185134	—	Lee et al. (2023)
	JB798	Air sample in a Cerrado cave	Brazil	PZ157759	PZ121189	—	PZ157671	—	This study
	FCCUFG 204	Air sample in a Cerrado cave	Brazil	PZ157760	PZ121190	—	PZ157672	—	This study
	FCCUFG 132	Air sample in a Cerrado cave	Brazil	PZ157761	PZ121191	PZ136718	—	—	This study
	FCCUFG 140	Air sample in a Cerrado cave	Brazil	PZ157762	PZ121192	—	—	—	This study
	JB810	Air sample in a Cerrado cave	Brazil	PZ157763	PZ121193	—	—	—	This study
	FCCUFG 111	Soil sample in a Cerrado cave	Brazil	PZ157764	PZ121194	PZ136719	PZ157673	—	This study
	FCCUFG 62	Soil sample in a Cerrado cave	Brazil	PZ157765	PZ121195	—	PZ157674	—	This study
*C. succulentum*	CBS 140466^T^	Dolphin, bronchus	USA	LN834618	LN834434	—	LN834530	—	Sandoval-Denis et al. (2016)
*C. velox*	CBS 119417^T^	*Bambusa* sp.	India	EF101388	DQ780361	—	JN906995	—	Bensch et al. (2018)
	CPC 22359	Indoor air sample	USA	MF474158	MF473308	—	MF473731	—	Bensch et al. (2018)
	JB797	Soil sample in a Cerrado cave	Brazil	PZ157766	—	—	—	—	This study
	JB790	Air sample in a Cerrado cave	Brazil	PZ157767	—	—	—	—	This study
	JB814	Soil sample in a Cerrado cave	Brazil	PZ157768	—	—	—	—	This study
	FCCUFG 63	Air sample in a Cerrado cave	Brazil	PZ157769	PZ121196	—	PZ157675	—	This study
	FCCUFG 128	Air sample in a Cerrado cave	Brazil	PZ157770	PZ121197	—	PZ157676	PZ136764	This study
	JB1953	Soil sample in a Cerrado cave	Brazil	PZ157771	PZ121198	PZ136720	PZ157677	PZ136765	This study
Outgroup									
*C. herbarum*	CBS 121621^T^	*Hordeum vulgare*	Netherlands	EF679516	EF679363	—	EF679440	—	Bensch et al. (2018)
	CBS 300.49	*Biscutella laevigata*	Switzerland	EF679511	EF679358	—	EF679434	—	Bensch et al. (2018)
*Cercospora beticola*	CBS 116456^T^	*Beta vulgaris*	Italy	AY840458	NR_121315	KT216555	AY840494	MH496355	Groenewald et al. (2005)

### Phylogenetic analyses

Sequences were edited using MEGA v. 11 (Tamura et al. 2021), and initial identifications were made using the BLASTn tool for comparison with the NCBI GenBank database. For a preliminary phylogenetic analysis, two large alignments were constructed using all *ACT* sequences from the isolates and available ex-type or reference sequences following recent publications on *Cladosporium*. The first set comprised sequences of the *C. cladosporioides* species complex (SC) (e.g. Pereira et al. 2022, 2024). The second set comprised sequences of *C. sphaerospermum*SC (e.g. Lee et al. 2023; Pereira et al. 2024). Following this initial analysis, representative isolates were selected to amplify ITS and *TEF1-α*, and single alignments were constructed for each. Subsequently, the alignments of each gene were combined into a matrix of three markers (*ACT*, ITS rDNA, and *TEF1-α*) and two markers (*ACT* and *TEF1-α*). In an attempt to improve phylogenetic resolution, *TUB* and *RPB2* gene sequences were generated for the new species and their sister species, and a more inclusive analysis of five genes (*ACT*, ITS, *RPB2*, *TEF1-α*, and *TUB*) was performed.

The alignments were performed using the online MAFFT software tool (Katoh and Standley 2013), and adjustments were made using MEGA v. 7 (Kumar et al. 2016). For the three-gene tree (*ACT*, ITS, and *TEF1-α*), *Cladosporium herbarum* (CBS 121621 and CBS 300.49) was used as the outgroup for both the *C. cladosporioides*SC and *C. sphaerospermum*SC. For the five-gene tree (*ACT*, ITS, *RPB2*, *TEF1-α*, and *TUB*), *Cercospora beticola* (CBS 116456) was used as the outgroup. Phylogenetic analyses used maximum likelihood (ML) and Bayesian inference (BI). ML analysis was conducted using IQ-TREE software v. 1.6.12 (Nguyen et al. 2015) and RAxML-HPC BlackBox v. 8.2.12 (Stamatakis 2014). The IQ-TREE ML analysis involved 5,000 replications, and the ultrafast bootstrap (UFboot2) method was used to calculate branch support (Hoang et al. 2018). The ModelFinder software, included in IQ-TREE v. 1.6.12 (Kalyaanamoorthy et al. 2017), was used to determine the partitioning strategy and models based on the Akaike information criterion for the dataset, using three (*ACT*, ITS, and *TEF1-α*) and five (*ACT*, ITS, *RPB2*, *TEF1-α*, and *TUB*) predefined data blocks. The RAxML-HPC BlackBox ML analysis was performed using the default system options in the CIPRES Science Gateway (Miller et al. 2010) with 1,000 bootstrap replicates. The BI analysis was conducted using MrBayes v. 3.2.7a (Ronquist et al. 2012) within the CIPRES Science Gateway, employing the same models and partitions as in the ML analysis (IQ-TREE). The BI analysis was conducted with 5 × 10^7^ generations and a burn-in value of 25%, with chains sampled every 1,000 generations.

The resulting individual and concatenated phylogenetic trees were visualised using FigTree v. 1.4.4 (Rambaut 2012) and analysed according to the GCPSR concept described by Taylor et al. (2000), where highly supported clades from single-locus and concatenated trees were compared to verify phylogenetic concordance between each gene and the combined analysis. The species treated here were divided into groups of possible synonyms and taxonomic novelties. Values ≥ 0.95 BI posterior probability (BPP) and bootstrap support (BS) from IQ-TREE UFboot2-BS and RAxML-BS analyses (both BS ≥ 70%) were plotted near the nodes. The final combined alignments were deposited in Figshare (doi: 10.6084/m9.figshare.31401723).

### Pairwise homoplasy index (PHI)

After analyses of the phylogenetic trees according to the GCPSR concept (Taylor et al. 2000), the pairwise homoplasy index (PHI) test was carried out using the SplitsTree App (Huson and Bryant 2024) to assess recombination between phylogenetic species related to taxonomic novelties and possible synonyms that were split by group. To verify this index, the combined dataset of the three loci (*ACT*, ITS, and *TEF1-α*) was used. For values above 0.05 (*p* > 0.05), no significant evidence of recombination was considered, and for values below 0.05 (*p* < 0.05), significant recombination was considered, indicating intraspecific variation. The phylogenetic networks were constructed using the LogDet transformation (Steel 1994) for the distance matrix and the Neighbor-Net algorithm (Bryant and Huson 2023) implemented in SplitsTree App (Huson and Bryant 2024). The resulting phylogenetic networks were prepared with Adobe Illustrator CS2 v. 12.0.0 (Adobe, USA).

### Morphological analyses of new species

Isolates were cultured in 90 mm diameter Petri dishes containing PDA (Kasvi, Pinhais, PR, Brazil), Malt Extract Agar (MEA), Oatmeal Agar (OA), Synthetic Nutrient-Deficient Agar (SNA), Czapek Yeast Extract Agar (CYA), Dichloran 18% Glycerol Agar (DG18), and Malt Yeast 40% Glucose Agar (MY40G) (Crous et al. 2009). Cultures were incubated at 25 °C for 14 days, after which colony characteristics such as diameter, pigment production, and colour were described according to Rayner (1970). The growth of the isolates was also assessed at 30 °C and 36 °C.

The morphological examinations were performed on PDA and SNA. For microscopic analysis, reproductive structures were mounted in lactic acid (85%), and 30 measurements of conidiophores, ramoconidia, conidiogenous cells, and conidia were taken using a Leica DM2500 microscope. Images were captured with a Flexacam C3 camera and processed using Leica Application Suite X v. 3.8.1.2 software. All analyses were conducted at the URM culture collection (Micoteca URM, UFPE, Recife, Brazil).

Isolates from this study are deposited in the working collection of Jadson Bezerra (JB), and their representatives are deposited in the Coleção de Culturas de Fungos da Universidade Federal de Goiás (FCCUFG), both housed in the Laboratório de Micologia (IPTSP-UFG, Goiânia, Brazil). Holotypes, as metabolically inactive cultures, are deposited in the University Recife Mycology (URM) collection (Micoteca URM, Universidade Federal de Pernambuco, Recife, Brazil), and permanent ex-holotype microscopic slides are deposited in the UFG herbarium (Herbário UFG, Goiânia, Brazil). Ex-type strains are deposited in the FCCUFG and URM culture collections.

## Results

### Abundance of *Cladosporium* species

The analysis of the 94 isolates revealed a predominance of *Cladosporium* obtained from air (96.5%) compared to soil (3.5%). In terms of species composition, the community was dominated by *C. bambusicola*, which alone accounted for 29.17% of the isolates, followed by *C. velox* (15.51%). The distribution of fungi among caves was also unequal, with most isolates concentrated in three caves: Lapa do Boqueirão, Gruna da Tarimba, and Lapa do Córrego das Dores (Table 2).

**Table 2.** Richness, distribution, and abundance (CFU) of *Cladosporium* species isolated from air and soil among six Cerrado caves in Brazil. black triangle = means a new record of the species in a cave. New species obtained in this study are shown in bold.

Species	New record	Caves						Total
		Air/Sediment						
		Lapa do Boqueirão	Garganta (Samambaia)	Lapa da Cachoeira do Funil	Lapa do Penhasco	Lapa do Córrego das Dores	Gruna da Tarimba	
*C. cladosporioides* complex								
*C. angulosum*	▲	0/0	0/0	0/0	0/0	1/0	0/0	1/0
*C. aulonemiae*	▲	8/0	0/0	0/0	0/0	0/0	0/0	8/0
*C. bambusicola*		1/1	2/0	3/1	15/2	90/0	11/0	122/4
*C. carsi* sp. nov.	▲	0/0	0/0	0/0	0/0	0/0	2/0	2/0
*C. chlamydosporiformans*	▲	3/0	0/0	0/0	9/0	1/0	36/0	49/0
*C. flabelliforme*	▲	0/0	0/0	0/0	1/0	0/0	0/0	1/0
*C. lacerdae* sp. nov.	▲	0/0	0/0	0/0	0/0	2/0	0/0	2/0
*C. macadamiae*	▲	0/0	0/0	1/0	0/1	3/0	0/0	4/1
*C. mambaiense* sp. nov.	▲	1/0	0/0	0/0	0/0	1/0	0/0	2/0
*C. nogueirae* sp. nov.	▲	3/0	0/0	0/0	0/0	1/0	0/0	4/0
*C. perangustum*	▲	0/0	1/0	0/0	0/0	0/0	0/0	1/0
*C. pernambucoense*		0/1	0/0	0/0	0/0	0/0	0/0	0/1
*C. propiciense* sp. nov	▲	0/0	10/0	0/0	0/0	0/0	0/0	10/0
*C. pruni-salicinae*	▲	0/0	0/0	0/0	0/0	0/0	9/0	9/0
*C. pseudocladosporioides*	▲	0/0	0/0	0/0	0/0	1/0	1/0	2/0
*C. puris*		2/0	0/0	0/0	0/0	0/0	0/0	2/0
*C. subuliforme*		8/0	0/0	0/0	0/0	9/0	28/0	45/0
*C. wenganense*	▲	6/0	0/0	0/0	0/0	7/0	0/0	13/0
*C. xanthochromaticum*		2/0	4/0	0/0	0/0	0/0	21/0	27/0
*C. sphaerospermum* complex								
*C. aciculare*	▲	0/0	0/0	1/0	0/0	0/0	0/0	1/0
*C. mesquitapaivae* sp. nov.	▲	0/0	0/0	0/0	0/0	2/0	0/0	2/0
*C. sphaerospermum*		49/0	0/0	0/0	0/2	0/0	0/1	49/3
*C. velox*	▲	60/3	1/0	0/0	0/3	0/0	0/0	61/6
Abundance		143/5	18/0	5/1	25/8	118/0	108/1	417/15
Richness		12	5	3	6	11	8	23

### Phylogenetic analyses

BLASTn searches in the NCBI GenBank database revealed that 78 of the sequences generated were related to *C. cladosporioides*SC and 16 to *C. sphaerospermum*SC. Initially, preliminary analyses were constructed with all the *ACT* gene sequences for each complex. In the *C. cladosporioides*SC, the analysis included 78 sequences from the isolates and 180 sequences from ex-type strains or reference material obtained from GenBank. The cave-dwelling isolates were placed into 14 known and five new species. In the *C. sphaerospermum*SC, the analysis included 16 sequences from the isolates and 46 sequences from ex-type strains or reference material obtained from GenBank. The isolates were classified as three known and one new species.

After building the *ACT* matrices for each complex, representatives were selected (41 isolates in the *C. cladosporioides*SC and 13 in the *C. sphaerospermum*SC) of the species with the greatest amplification success to construct the combined alignment, with each isolate having at least two of the genes: *ACT*, ITS, and *TEF1-α*.

### *Cladosporium cladosporioides* complex

**Figure 3.**
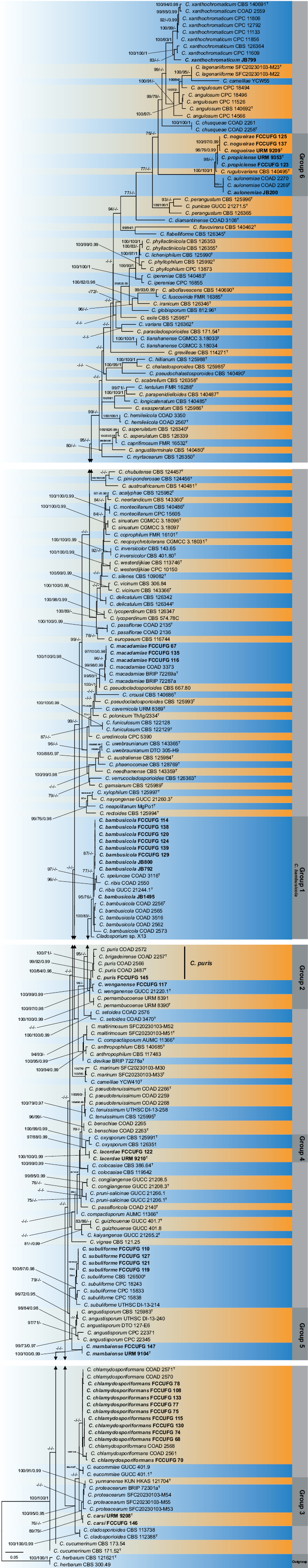
Maximum likelihood tree of the *Cladosporium cladosporioides* complex based on a combined matrix of *ACT*, ITS, and *TEF1-α* sequences. The species obtained in this study are highlighted in bold. Ex-type strains = ^T^. IQ-TREE-BS values ≥ 70%, RAxML-BS ≥ 70%, and BPP ≥ 0.95 are included next to the nodes. The tree was rooted with *Cladosporium herbarum* (CBS 121621 and CBS 300.49).

The combined matrix of three genes (*ACT*, ITS, and *TEF1-α*) (Fig. 3) consisted of 222 sequences, including the outgroup *C. herbarum* (CBS 300.49 and CBS 121621), with 1,015 characters, including gaps (*ACT*: 1–227, ITS: 228–728, and *TEF1-α*: 729–1,015), of which 587 were conserved, 384 variable, and 304 parsimony-informative. For the ML analysis, the best score of the tree and the final value of the optimisation probability were –ln = –14833.319662, and the estimated base frequencies were A = 0.224578, C = 0.299577, G = 0.240162, and T = 0.235683, with substitution rates of AC = 2.066477, AG = 5.398780, AT = 2.207302, CG = 1.383126, CT = 7.389053, and GT = 1.000000, and the gamma distribution shape parameter: α = 0.838359. The models used for IQ-TREE ML and BI were GTR+G for *ACT*, JC+G for ITS, and GTR+I+G for *TEF1-α*. GTR+I+G was used for the RAxML-HPC BlackBox ML analysis. Additionally, individual trees were constructed for each of the studied genes (Suppl. materials 1–3).

The analysis of three loci (*ACT*, ITS, and *TEF1-α*) proved to be the most effective for resolving the species (Fig. 3). Some of the phylogenetic lineages varied in position among the genes analysed, with some genes being more informative than others when analysed individually. The *ACT* and *TEF1-α* genes provided a topology with greater similarity when compared to the combined matrix (Fig. 3, Suppl. materials 1, 3). *ACT* and *TEF1-α* provided more information and were the most powerful markers for resolving the species analysed, resolving 16 of the 19 species in *ACT* (84% of the isolates sequenced for this gene) and 12 of the 12 species in *TEF1-α* (100% of the isolates sequenced for this gene) (Suppl. materials 1, 3). ITS was the least informative gene, as it did not separate any of the species analysed (Suppl. material 2).

Based on integrated evidence from phylogenetic analyses (Suppl. materials 1, 3), overlapping morphological characteristics, and PHI analysis, two synonymies are proposed. Certain isolates grouped into well-supported clades with known species but could not be distinguished from them. Consequently, *C. speluncae* and *C. ribis* are synonymised under *C. bambusicola* (Group 1), and *C. brigadeirense* is synonymised under *C. puris* (Group 2).

Furthermore, based on phylogenetic analyses (Fig. 3, Suppl. materials 1, 3), five new species are proposed (*C. carsi*, *C. lacerdae*, *C. mambaiense*, *C. nogueirae*, and *C. propiciense*), and the occurrence of 14 known species is reported (*C. angulosum*, *C. aulonemiae*, *C. bambusicola*, *C. chlamydosporiformans*, *C. flabelliforme*, *C. macadamiae*, *C. perangustum*, *C. pernambucoense*, *C. pruni-salicinae*, *C. pseudocladosporioides*, *C. puris*, *C. subuliforme*, *C. wenganense*, and *C. xanthochromaticum*) among the cave isolates. In addition, the first occurrence in caves of eight of the 14 known species is reported (*C. angulosum*, *C. aulonemiae*, *C. bambusicola*, *C. chlamydosporiformans*, *C. macadamiae*, *C. puris*, *C. subuliforme*, and *C. xanthochromaticum*), along with the novel species introduced here, expanding the geographical distribution of the *C. cladosporioides*SC in Brazilian caves.

### *Cladosporium sphaerospermum* complex

**Figure 4.**
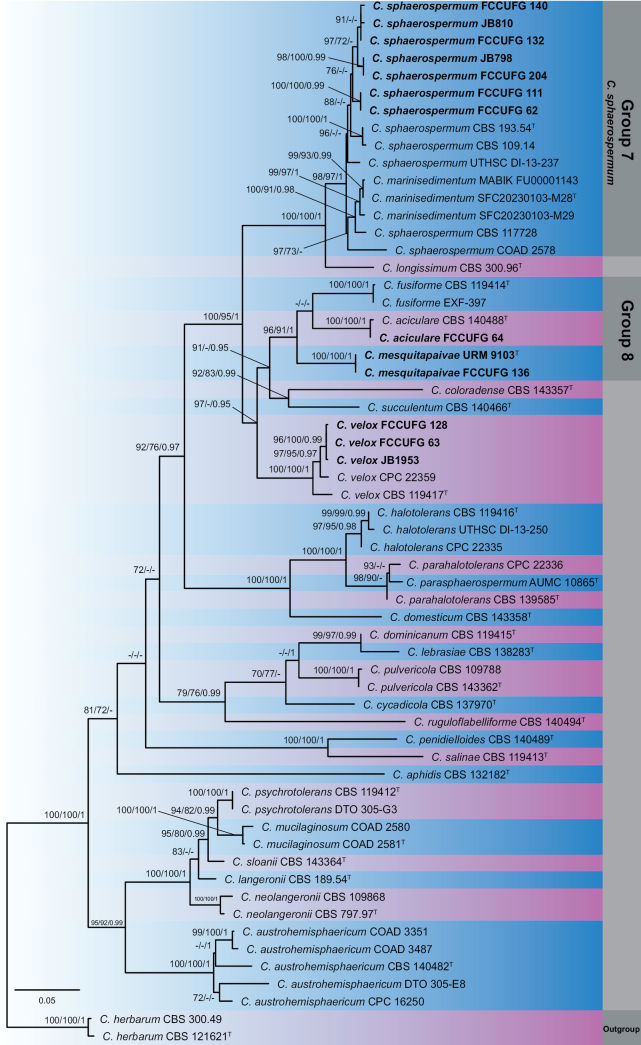
Maximum likelihood tree of the *Cladosporium sphaerospermum* complex based on a combined matrix of *ACT*, ITS, and *TEF1-α* sequences. The species obtained in this study are highlighted in bold. Ex-type strains = ^T^. IQ-TREE-BS values ≥ 70%, RAxML-BS ≥ 70%, and BPP ≥ 0.95 are included next to the nodes. The tree was rooted with *Cladosporium herbarum* (CBS 121621 and CBS 300.49).

The combined matrix of three genes (*ACT*, ITS, and *TEF1-α*) (Fig. 4) consisted of 60 sequences, including the outgroup *C. herbarum* (CBS 300.49 and CBS 121621), with 1,110 characters, including gaps (*ACT*: 1–214, ITS: 215–766, and *TEF1-α*: 767–1,110), of which 625 were conserved, 452 variable, and 373 parsimony-informative. For the ML analysis, the best score of the tree and the final value of the optimisation probability were –ln = –8885.879243, and the estimated base frequencies were A = 0.222970, C = 0.296723, G = 0.245835, and T = 0.234472, with substitution rates of AC = 1.751500, AG = 3.067940, AT = 2.048440, CG = 1.042000, CT = 5.185987, and GT = 1.000000, and the gamma distribution shape parameter: α = 1.353097. The models used for IQ-TREE ML and BI were HKY+I+G for *ACT*, SYM+I+G for ITS, and GTR+I+G for *TEF1-α*. GTR+I+G was used for the RAxML-HPC BlackBox ML analysis. Additionally, individual trees were constructed for each of the genes studied (Suppl. materials 4–6).

The analysis of three loci (*ACT*, ITS, and *TEF1-α*) proved to be the best for resolving the species (Fig. 4). Some of the phylogenetic lineages varied in position between the analysed genes. However, for this complex, all the genes (*ACT*, ITS, and *TEF1-α*) provided a topology similar to that observed in the combined matrix (Fig. 4, Suppl. materials 4–6). Thus, *ACT* and *TEF1-α* provided more information and were the most powerful markers for resolving all four (100%) species analysed (Suppl. materials 4, 6). The ITS sequences, although informative, formed a separate lineage for isolate FCCUFG 140, distinguishing it from the species in which it clustered (*C. sphaerospermum*) (Suppl. material 5).

Based on integrated evidence from phylogenetic analyses (Suppl. materials 4–6), overlapping morphological characteristics, and the PHI test, *C. marinisedimentum* is here synonymised under *C. sphaerospermum* (Group 7). Additionally, phylogenetic analyses (Fig. 4, Suppl. materials 4–6) supported the introduction of a new species (*C. mesquitapaivae*) and the identification of isolates belonging to three known species (*C. aciculare*, *C. sphaerospermum*, and *C. velox*). In addition, the first occurrence in caves of two of the three known species is reported (*C. aciculare* and *C. velox*), along with the new species introduced here, expanding the geographical distribution of the *C. sphaerospermum*SC in Brazilian caves.

### PHI analysis

**Figure 5.**
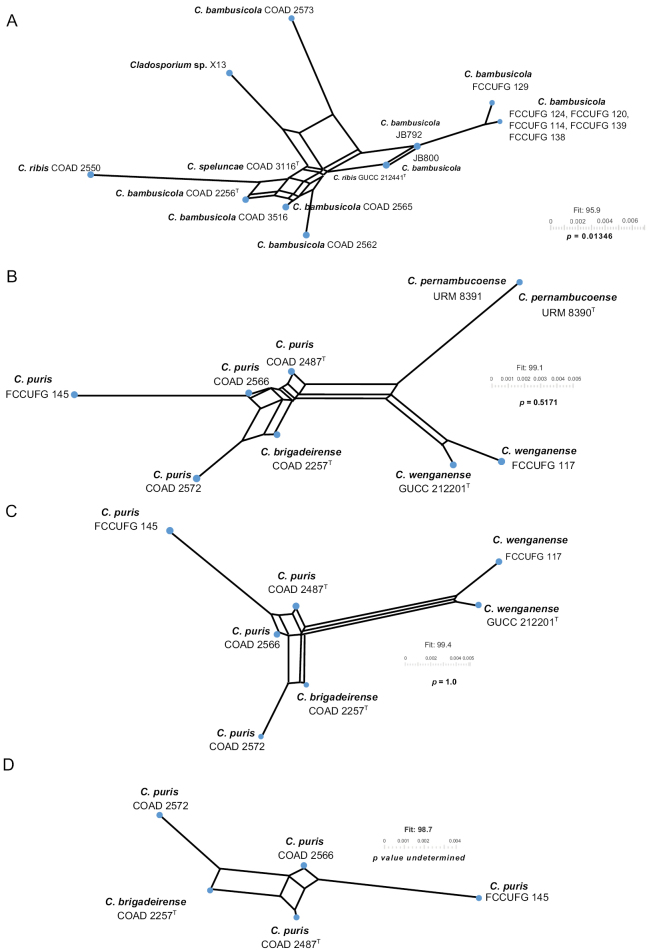
NeighborNet phylogenetic networks of phylogenetic Groups 1 and 2 of species of the *C. cladosporioides*SC based on the LogDet transformation for a combined dataset of three loci (*ACT*, ITS, and *TEF1-α*). **A–D**. The results of the PHI test are presented next to each set of species tested, and if the *p*-value is < 0.05, there is recombination. Strains with type status are indicated with a “T”.

To complement the delimitation of *Cladosporium* species, a PHI analysis was conducted, selecting groups that comprised known and phylogenetically new species. For this, the concatenated alignment (*ACT*, ITS, and *TEF1-α*) was used and divided into eight groups, following GCPSR (Taylor et al. 2000); six belonged to the *C. cladosporioides*SC and two to the *C. sphaerospermum*SC (Figs 3, 4). Group 1 (Fig. 3) included isolates from this study and ex-type strains/reference material of *C. bambusicola*, *C. speluncae*, *C. ribis*, and *Cladosporium* sp. X13 (see Pereira et al. 2022). For this group, the PHI test indicated evidence of genetic recombination (*p* = 0.01346) (Fig. 5A), suggesting intraspecific variation and casting doubt on their validity as separate taxonomic entities; these data were also important in the decision to synonymise *C. ribis* and *C. speluncae* under *C. bambusicola*.

For Group 2 (Fig. 3), three analyses were carried out using the PHI test (Fig. 5B–D). All analyses included isolates from this study. In the first analysis, ex-type/reference strains of *C. brigadeirense*, *C. puris*, *C. wenganense*, and *C. pernambucoense* were included. In the second analysis, *C. brigadeirense*, *C. puris*, and *C. wenganense* were included. In the third analysis, *C. brigadeirense* and *C. puris* were included. For this group, in the first and second analyses, the PHI test did not indicate evidence of genetic recombination (*p* = 0.5171 and *p* = 1.0, respectively) (Fig. 5B, C). In the third analysis, there were not enough informative characters to use the PHI test (Fig. 5D). Given the limitations of the PHI analysis in this case, the synonymisation of *C. brigadeirense* under *C. puris* was recommended based on morphological characters and the individual and combined phylogenetic trees (Fig. 3, Suppl. materials 1, 3).

**Figure 6.**
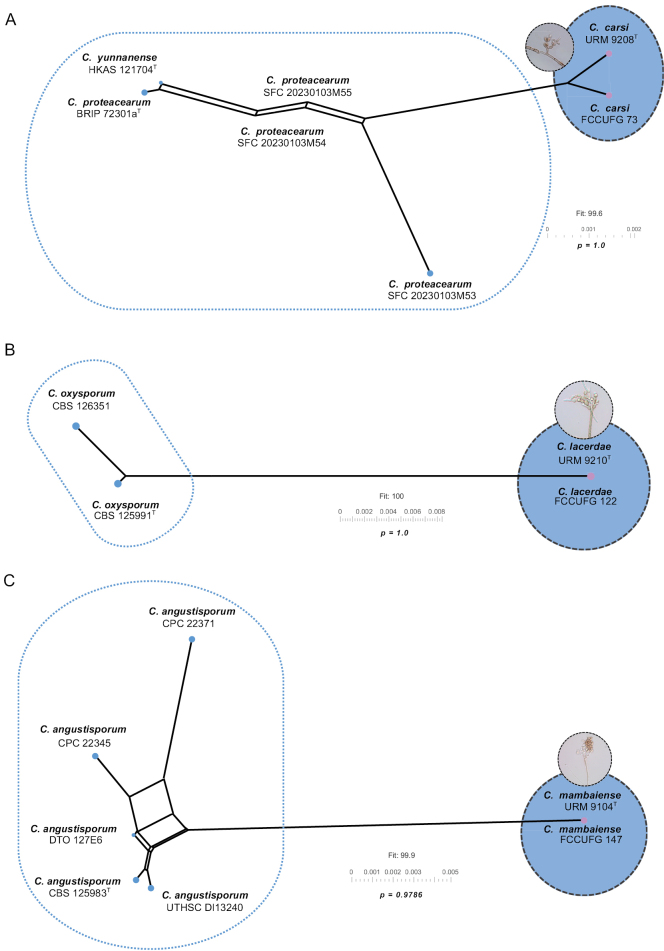
NeighborNet phylogenetic networks of phylogenetic Groups 3, 4, and 5 of species of the *C. cladosporioides*SC based on the LogDet transformation for a combined dataset of three loci (*ACT*, ITS, and *TEF1-α*). **A–C**. The results of the PHI test are presented next to each set of species tested, and if the *p*-value is < 0.05, there is recombination. Strains with type status are indicated with a “T”.

Group 3 (Fig. 3) included *C. proteacearum*, *C. yunnanense*, and *C. carsi* sp. nov. For this group, the PHI test indicated no evidence of genetic recombination (*p* = 1.0) (Fig. 6A). The same was observed for Group 4 (Fig. 3), which included *C. oxysporum* and *C. lacerdae* sp. nov. (PHI, *p* = 1.0) (Fig. 6B). Group 5 (Fig. 3) included *C. angustisporum* and *C. mambaiense* sp. nov., and the PHI test indicated no evidence of genetic recombination (*p* = 0.9786) (Fig. 6C). These results also support the introduction of these three new species.

**Figure 7.**
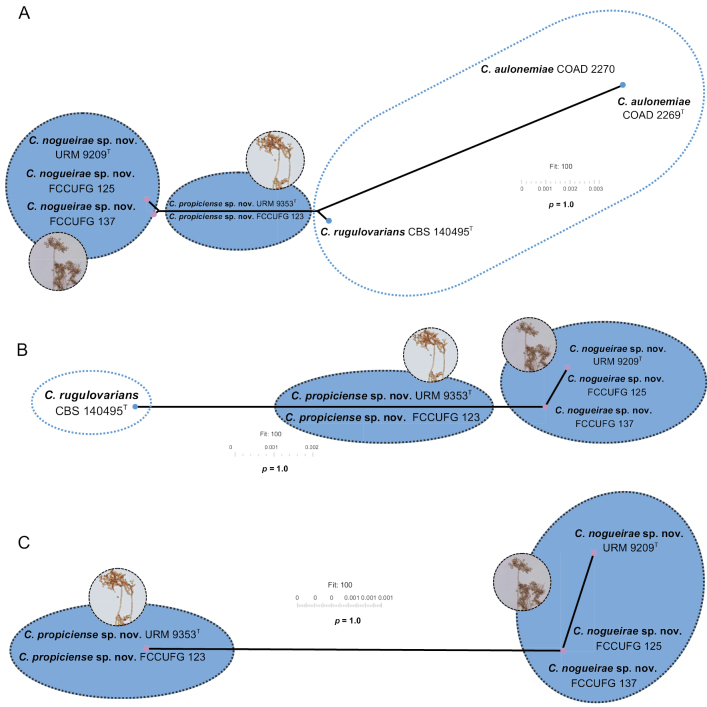
NeighborNet phylogenetic networks of phylogenetic Group 6 of species of the *C. cladosporioides*SC based on the LogDet transformation for a combined dataset of three loci (*ACT*, ITS, and *TEF1-α*). **A–C**. The results of the PHI test are presented next to each set of species tested, and if the *p*-value is < 0.05, there is recombination. Strains with type status are indicated with a “T”.

For Group 6 (Fig. 3), three analyses were carried out using the PHI test. The first analysis included *C. nogueirae* sp. nov., *C. propiciense* sp. nov., *C. rugulovarians*, and *C. aulonemiae*; the second included *C. nogueirae* sp. nov., *C. propiciense* sp. nov., and *C. rugulovarians*; and the third included *C. nogueirae* sp. nov. and *C. propiciense* sp. nov. For this group, the PHI test in the three analyses indicated no evidence of genetic recombination (*p* = 1.0 for all three analyses) (Fig. 7A–C). Therefore, this test also supports the introduction of these new species.

**Figure 8.**
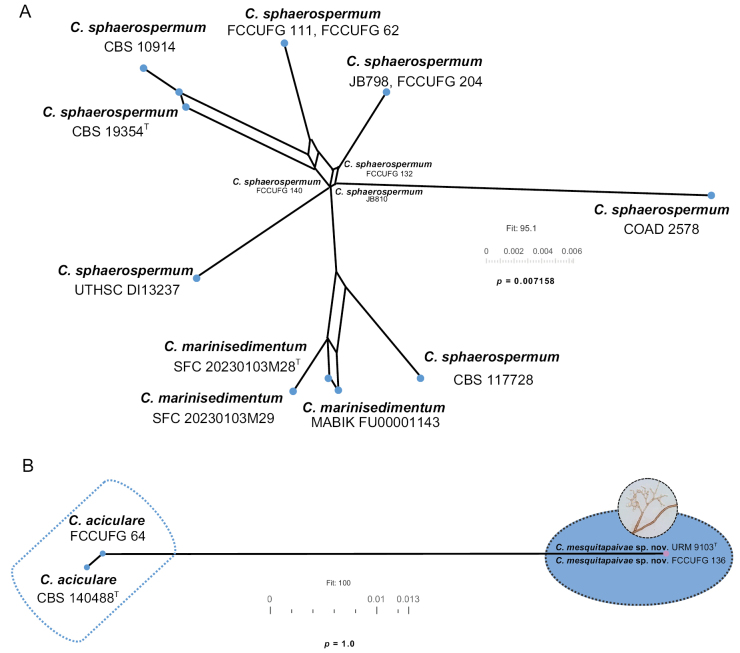
NeighborNet phylogenetic networks of phylogenetic Groups 7 and 8 of species of the *C. sphaerospermum*SC based on the LogDet transformation for a combined dataset of three loci (*ACT*, ITS, and *TEF1-α*). **A, B**. The results of the PHI test are presented next to each set of species tested, and if the *p*-value is < 0.05, there is recombination. Strains with type status are indicated with a “T”.

For the *C. sphaerospermum* complex, the PHI test was carried out for two groups. Group 7 (Fig. 4) included the ex-type strains/reference material of *C. sphaerospermum*, *C. marinisedimentum*, and isolates from this study. For this group, the PHI test indicated evidence of genetic recombination (*p* = 0.007158) (Fig. 8A). Therefore, *C. marinisedimentum* is here synonymised under *C. sphaerospermum* based on these results, morphology, and phylogeny. Group 8 (Fig. 4) included *C. aciculare*, *C. fusiforme*, and *C. mesquitapaivae* sp. nov. For this group, the PHI test did not indicate evidence of genetic recombination between *C. aciculare* and *C. mesquitapaivae* (Fig. 8B) or between *C. fusiforme* and *C. mesquitapaivae* (data not shown), reinforcing the identification of this species as an independent lineage among known species.

### *TUB* and *RPB2* barcode analysis

**Figure 9.**
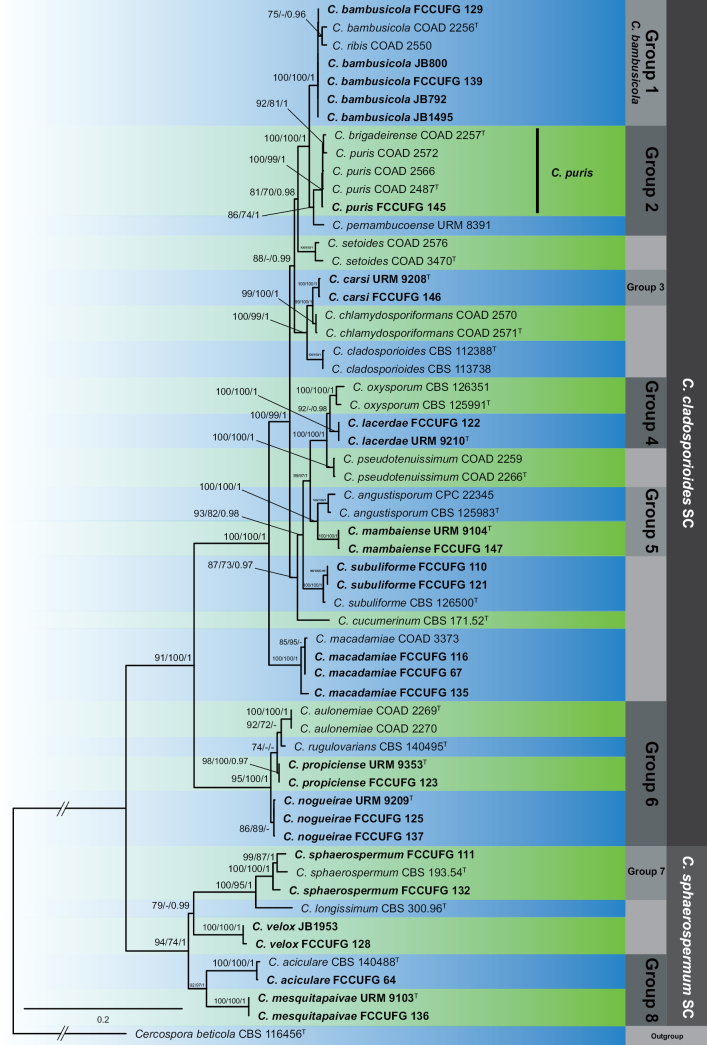
Maximum likelihood tree of the *Cladosporium cladosporioides* and *C. sphaerospermum* complexes based on a combined matrix of *ACT*, ITS, *RPB2*, *TEF1-α*, and *TUB* sequences. The species obtained in this study are highlighted in bold. Ex-type strains = ^T^. IQ-TREE-BS values ≥ 70%, RAxML-BS ≥ 70%, and BPP ≥ 0.95 are included next to the nodes. The tree was rooted with *Cercospora beticola* (CBS 116456).

After analysis of the three genes (*ACT*, ITS, and *TEF1-α*), the *TUB* and *RPB2* barcodes of ex-type strains of the new species and their relatives and the strains of synonymised species were sequenced, including 47 representatives of the *C. cladosporioides*SC and 10 of the *C. sphaerospermum*SC, and analysed in a combined matrix of five genes (*ACT*, ITS, *RPB2*, *TEF1-α*, and *TUB*) (Fig. 9).

The five-gene matrix consisted of 58 sequences, including the outgroup *Cercospora beticola* (CBS 116456), with 2,540 characters, including gaps (*ACT*: 1–218, ITS: 219–725, *RPB2*: 726–1,569, *TEF1-α*: 1,570–1,879, and *TUB*: 1,880–2,540), of which 1,464 were conserved, 1,004 variable, and 679 parsimony-informative. For the ML analysis, the best score of the tree and the final value of the optimisation probability were –ln = –14304.312891, and the estimated base frequencies were A = 0.233078, C = 0.294117, G = 0.252305, and T = 0.220499, with substitution rates of AC = 1.549483, AG = 3.888228, AT = 1.632386, CG = 1.013659, CT = 6.445771, and GT = 1.000000, and the gamma distribution shape parameter: α = 0.484084. The models used for IQ-TREE ML and BI were HKY+G for *ACT*, JC+I+G for ITS, SYM+G for *RPB2*, GTR+G for *TEF1-α*, and GTR+I+G for *TUB.* GTR+I+G was used for the RAxML-HPC BlackBox ML analysis.

The analysis of five loci (*ACT*, ITS, *RPB2*, *TEF1-α*, and *TUB*) proved to be efficient for delimiting *Cladosporium* species, mainly in the *C. cladosporioides*SC (Fig. 9). The *RPB2* and *TUB* genes provided important information and proved to be effective markers for identifying the species analysed, enabling the identification of 11 (100%) of the species sequenced for *RPB2*, while *TUB* identified 10 (91%) of the 11 species sequenced for this gene (Suppl. materials 7, 8). Therefore, the inclusion of *TUB* and *RPB2* provided more information for the phylogenetic resolution of proposed new species and synonymies (Fig. 9, Suppl. materials 7, 8).

### Taxonomy

#### Cladosporium aciculare

Taxon classificationAnimaliaCladosporialesCladosporiaceae

Bensch, Crous & U. Braun, (2015)

0FE000A4-9A85-5384-B346-968EFEC353F1

##### Typification.

AUSTRALIA • isol. from *Syzygium corynanthum* (*Myrtaceae*), Mar. 2009, P.W. Crous (holotype CBS H-22359, ex-type culture CBS 140488 = CPC 16547).

##### Description and illustration.

See Bensch et al. (2015).

##### Material examined.

BRAZIL • Goiás state, Mambaí municipality, Lapa da Cachoeira do Funil cave, isol. from air, May 2023, coll. J.F.S.A. Prazeres, E.O. Fonseca & J.D.P. Bezerra, isol. E.O. Fonseca, isolate FCCUFG 64.

##### Notes.

*Cladosporium aciculare* (*C. sphaerospermum*SC) was proposed by Bensch et al. (2015) and was isolated from *Syzygium corynanthum* (*Myrtaceae*) in Australia. In this study, isolate FCCUFG 64 from the Lapa da Cachoeira do Funil cave clustered together with the ex-type strain of *C. aciculare* (Figs 4, 9) and is here identified as this species. This study is the first report of *C. aciculare* from a Brazilian cave (found at a sampling point inside the cave).

#### Cladosporium angulosum

Taxon classificationAnimaliaCladosporialesCladosporiaceae

Sand.-Den., Deanna A. Sutton & Guarro, (2016)

2C15E235-9176-5AC2-9618-E578F0FE082C

##### Typification.

USA • isol. from human bronchoalveolar lavage fluid, Sep. 2008, D.A. Sutton (holotype CBS H-22380, ex-type culture CBS 140692 = UTHSC DI-13-235 = FMR 13348).

##### Description and illustration.

See Sandoval-Denis et al. (2016).

##### Material examined.

BRAZIL • Goiás state, Mambaí municipality, Lapa do Córrego das Dores cave, isol. from air, May 2023, coll. J.F.S.A. Prazeres, E.O. Fonseca & J.D.P. Bezerra, isol. E.O. Fonseca, isolate FCCUFG 202.

##### Notes.

*Cladosporium angulosum* (*C. cladosporioides*SC) was introduced by Sandoval-Denis et al. (2016) for a fungus isolated from human bronchoalveolar lavage in the USA. Previous records have reported *C. angulosum* in freshwater environments in Korea, on plants, in indoor environments, and as an endophytic fungus from *Camellia sinensis* (*Theaceae*) (Goh et al. 2020; Lv et al. 2023). The present study reports this species for the first time from a Brazilian cave (found at a sampling point inside the cave).

#### Cladosporium aulonemiae

Taxon classificationAnimaliaCladosporialesCladosporiaceae

P.P. Costa, A.W.C. Rosado & O.L. Pereira, (2022)

B1F8B60C-48AC-51B1-89E1-254CD56A3C07

##### Typification.

BRAZIL • Minas Gerais: Araponga, at “Parque Estadual Serra do Brigadeiro”, isol. from decayed leaf of *Aulonemia amplissima* (*Poaceae*), Mar. 2016, P.P. Costa (holotype VIC 44413, ex-type culture COAD 2269).

##### Description and illustration.

See Costa et al. (2022).

##### Material examined.

BRAZIL • Goiás state, Vila Propício municipality, Lapa do Boqueirão cave, isol. from air, May 2022, coll. P.H. Félix-Oliveira & J.D.P. Bezerra, isol. P.H. Félix-Oliveira, isolate JB200.

##### Notes.

*Cladosporium aulonemiae* (*C. cladosporioides*SC) was proposed by Costa et al. (2022) and isolated from decayed leaves of *Aulonemia amplissima* (*Poaceae*) in Brazil. In this study, isolate JB200 from the Lapa do Boqueirão cave clustered with the ex-type strain of *C. aulonemiae* (Fig. 3). This study reports the first occurrence of *C. aulonemiae* from a Brazilian cave (found at a sampling point outside the cave).

#### Cladosporium bambusicola

Taxon classificationAnimaliaCladosporialesCladosporiaceae

P.P. Costa, A.W.C. Rosado & O.L. Pereira, (2022)

8AB8205F-033A-550C-82D6-190350ED7E0D

##### Synonyms.

*Cladosporium ribis* Y.Q. Yang & Yong Wang bis, J. Fungi 9(no. 250): 13 (2023), syn. nov.

*Cladosporium speluncae* T.O. Condé, A.W.C. Rosado & O.L. Pereira, Braz. J. Microbiol. 54: 3027 (2023), syn. nov.

##### Typification.

BRAZIL • Minas Gerais, Araponga, at “Parque Estadual Serra do Brigadeiro”, isol. from decayed leaves of *Aulonemia amplissima* (*Poaceae*), Mar. 2016, (holotype VIC 44237, ex-type culture COAD 2256).

##### Description and illustration.

See Costa et al. (2022).

##### Material examined.

BRAZIL • Goiás state, Mambaí municipality, Lapa do Penhasco cave, isol. from air (isolates JB1552, FCCUFG 120, FCCUFG 138, FCCUFG 124, and JB1496) and soil (isolates JB2497 and JB2515), May 2023, coll. J.F.S.A. Prazeres, E.O. Fonseca & J.D.P. Bezerra, isol. J.F.S.A. Prazeres, Lapa do Córrego das Dores cave, isol. from air by E.O. Fonseca (isolates JB1545, FCCUFG 129, FCCUFG 139, JB1516, JB544, JB1547, JB1524, JB1526, and JB1502), Gruna da Tarimba cave, isol. from air by E.O. Fonseca (isolates JB1511, JB1529, FCCUFG 114, JB1495, and JB1492), and Lapa da Cachoeira do Funil cave, isol. from air (isolates JB1534, JB1507, JB1504) and soil (isolates JB1964) by E.O. Fonseca; • Goiás state, Vila Propício municipality, from Lapa do Boqueirão cave, isol. from air (isolate JB792) and soil (isolate JB800), May 2022, coll. P.H. Félix-Oliveira & J.D.P. Bezerra, isol. P.H. Félix-Oliveira, Garganta (Samambaia) cave, isol. from air, Sep. 2022, coll. P.H. Félix-Oliveira & J.D.P. Bezerra, isol. P.H. Félix-Oliveira (isolates JB1457 and JB1458).

##### Notes.

*Cladosporium bambusicola* (*C. cladosporioides*SC) was described as a saprophytic fungus on decayed leaves of native bamboo in Brazil (Costa et al. 2022); *C. ribis* was isolated from leaves of *Ribes burejense* (*Grossulariaceae*) in China (Yang et al. 2023); and *C. speluncae* was isolated from cave soil in Brazil (Dutra et al. 2023). In the phylogenetic analyses (Figs 3, 9, Suppl. materials 1, 3, 7), these species are indistinguishable, have overlapping morphological characteristics (Table 3), and show few differences among their DNA sequences: the type sequence of *C. ribis* (GUCC 21244.1) differs from the type sequence of *C. bambusicola* (COAD 2256) by 2 bp in the *ACT* sequence and by 7 bp in the *TEF1-α* sequence; the ITS sequence has no base pair difference. The type sequence of *C. speluncae* (COAD 3116) differs from the type sequence of *C. bambusicola* (COAD 2256) by 3 bp in the *ACT* sequence and by 2 bp in the *TEF1-α* sequence; the ITS sequence has no base pair difference. In addition, PHI analysis revealed significant recombination, indicating intraspecific variation (Fig. 5A). Therefore, these analyses propose that *C. ribis* and *C. speluncae* are synonyms of *C. bambusicola*. In this study, *C. bambusicola* was the most frequently isolated species from caves of the Brazilian savannah, expanding its geographical distribution and ecology. These isolates were found at sampling points both inside and outside the caves.

**Table 3.** Main morphological features of *Cladosporium* species treated (synonyms and new) in this study. Measurements are in μm.

Groups	Species	Conidiophores	Conidiogenous cells	Ramoconidia	Secondary ramoconidia	Intercalary conidia	Terminal conidia	References
Group 1	*C. bambusicola*	23.96–178.99 × 2.40–6.17	9.13–47.04 × 1.58–4.19	33.90–39.71 × 4.34–5.80	6.28–26.25 × 2.06–5.51	4.16–10.79 × 2.66–5.27	-	Costa et al. 2022
	*C. ribis*	8.0–103.5 × 2.0–4.0	-	-	4.0–8.5 × 2.0–3.5	2.5–4.5 × 2.0–3.0	-	Yang et al. 2023
	*C. speluncae*	16.4–394 × 1.6–4.6	5–29.6 × 1.8–4	6–27 × 1.5–4	7–15.5 × 3–4.5	3.6–9 × 2–4.6	-	Dutra et al. 2023
Group 2	*C. brigadeirense*	25.07–222.22 × 2.51–4.80	9.32–25.16 × 1.73–3.65	-	4.48–13.52 × 2.21–3.51	3.02–6.81 × 2.18–3.60	-	Costa et al. 2022
	*C. puris*	44–225 × 2–3	7.5–42.5 × 2–3.5	8–17.5 × 2.5–4	5–12.5 × 2–3.5	3.5–6 × 2–3.5	2.5–4.5 × 2–3	Freitas et al. 2021
Group 3	*C. carsi* sp. nov.	102–304 × 2.5–4	4–29 × 2–4	8–27 × 2–4	7.5–15 × 2–4	4.5–12 × 3–5	2–7 × 1–3	This study
	*C. proteacearum*	150–500 × 2.5–4	-	12–48 × 3–5	5–10 × 3–4	4–5 × 2–3	4–5 × 2–3	Prasannath et al. 2021
Group 4	*C. lacerdae* sp. nov.	90–250 × 2.5–4	6–27 × 2–4	10.5–18.5 × 2–4	8.5–24 × 2.5–4	9–14 × 2.5–4	2–6 × 2–3	This study
	*C. oxysporum*	40–720 × 2–4	14–46 × 0.8–1.5	-	7–24 × 2.5–4	11 × 2.5–3.5	3–5 × 2–3	Bensch et al. 2012
Group 5	*C. angustisporum*	22–280 × 1.5–4	10–27 × 1–1.5–2	18–55 × 2.5–3	6–26 × 2–3	4–13 × 1.5–3	3–6.5 × 1.5–2	Bensch et al. 2012
	*C. mambaiense* sp. nov.	110–554 × 2.5–4	7–29 × 3–5	16.5–25 × 2–5	11–30 × 2–3.5	7–13 × 2.5–4	3–5 × 1–3	This study
Group 6	*C. nogueirae* sp. nov.	108–384 × 2.5–4	5–11 × 1–3	11–22 × 2–5	5.5–12 × 1–3	6–8 × 2–6	4–7 × 1–3	This study
	*C. propiciense* sp. nov.	90–420 × 2.5–3.5	5–11 × 1–3	10–27 × 2–5	4–16 × 1–3	6–9 × 2–6	4–7 × 1–3	This study
	*C. rugulovarians*	475 × 3.5–5	60 × 1–1.5	20–55 × 3–4.5	(9–)11–24(–30) × (2–)2.5–5.5	5–8.5(–10) × 3.5–5(–6)	3–6.5 × 3–5	Bensch et al. 2015
Group 7	*C. sphaerospermum*	10–300(–500) × 2–5	10–30 × 1–1.5(–2)	12–35 × 3–5	-	4–10 × 3–5	2–5 × 2–4	Bensch et al. 2012
	*C. marinisedimentum*	220 × 1.5–3.7	16.2–41.5 × 2–3.2	9.2–31.8 × 1.7–3.3	7.8–31.6 × 2.1–3.4	3.2–9.1 × 2.1–3.6	2.6–4.6 × 2.1–3.2	Lee et al. 2023
Group 8	*C. aciculare*	28–250 × 3–4	15–40 × 2–5	22–40 × 2–2.5	5–23 × 2–3	4–10 × 2–2.5	3–4 × 1.5–2	Bensch et al. 2015
	*C. fusiforme*	10–100 × 2–4	-	-	5–23 × 2–3	2.5–6.5 × 2–3	-	Zalar et al. 2007
	*C. mesquitapaivae* sp. nov.	27–90 × 2–3	4–7 × 1–3	11–12.5 × 2–3.5	5–8 × 2–3.5	3–7 × 2–3	2–3 × 1.5	This study

#### Cladosporium carsi

Taxon classificationAnimaliaCladosporialesCladosporiaceae

J.F.S.A. Prazeres, P.H. Félix-Oliveira, E.O. Fonseca, Souza-Motta & J.D.P. Bezerra sp. nov.

41893099-4D82-597D-A133-D9C07D1BF5B2

860817

Fig. 10

##### Etymology.

The epithet is based on “carsus”, the original Latin designation from which “karst” is derived.

##### Typification.

BRAZIL • Goiás state, Mambaí municipality, Gruna da Tarimba cave, 14°24'43"S, 46°10'30"W, isol. from air, May 2023, coll. J.F.S.A. Prazeres, J.D.P. Bezerra & E.O. Fonseca, isol. E.O. Fonseca [**holotype**URM 9208HT (preserved in metabolically inactive state), UFG 39349 (microscopic slide ex-holotype), culture ex-type URM 9208 = FCCUFG 73].

**Figure 10.**
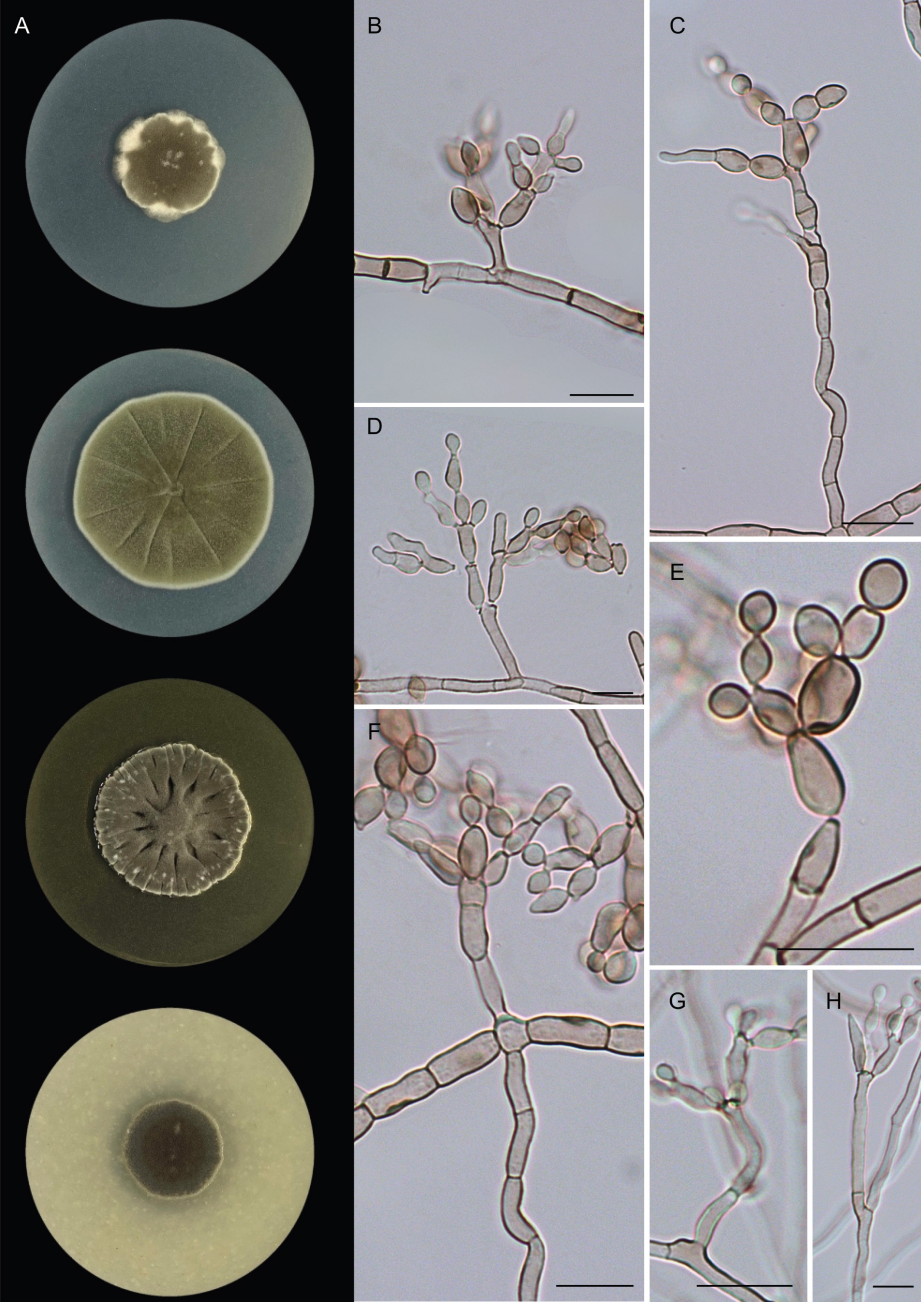
*Cladosporium carsi* (URM 9208, ex-type). **A**. Colonies (from top to bottom) on SNA, PDA, MEA, and OA after 14 days at 25 °C in the dark. **B**. Micronematous conidiophore, ramoconidia, conidiogenous cells, and conidia. **C**. Macronematous conidiophore, ramoconidia, conidiogenous cells, and conidia. **D–F**. Conidiophores, ramoconidia, conidiogenous cells, and conidial chains. **G**. Curved conidiophore, ramoconidia, secondary ramoconidia, conidiogenous cells, and conidia. **H**. Erect conidiophore, ramoconidia, secondary ramoconidia, conidiogenous cells, and conidia. Scale bars: 10 μm.

##### Description.

***Hyphae*** superficial and immersed; hyphae septate, branched, subhyaline to dark brown, smooth and thick-walled. ***Conidiophores*** solitary, micronematous, semimicronematous, and macronematous, arising terminally and laterally from hyphae, erect, cylindrical, non-nodular, geniculate, septate, usually branched, subhyaline, with thickened and refractive wall, 102–304 × 2.5–4 μm. ***Conidiogenous cells*** integrated, cylindrical or subcylindrical, terminal and intercalary, thickened and somewhat darkened conidiogenous loci, 4–29.5 × 2–4 μm. ***Ramoconidia*** often formed, aseptate, cylindrical, dark brown, smooth, with protruding hila and truncated base, 8–27 × 2–4 μm. ***Secondary ramoconidia*** septate, ellipsoidal to subcylindrical, usually attenuated at centre, 8–15 × 2–3.5 μm. ***Intercalary conidia*** numerous, formed in branched chains, aseptate, subhyaline to pale olivaceous brown, limoniform, ovoid and ellipsoidal, 4–12 × 2–4 μm; ***terminal conidia*** small, ovoid to ellipsoidal, 2–7 × 1–3 μm.

##### Culture characteristics.

Colonies average diameters (mm) of 29, 53, 41, and 26 on SNA, PDA, MEA, and OA, respectively. On SNA, greenish olivaceous, with a lighter, well-defined edge, reverse olivaceous black, aerial mycelium, smooth, compact at the centre, without prominent exudates. On PDA, greenish olivaceous, velvety texture, distinct radial furrowing pattern, reverse olivaceous black, aerial mycelium, velvety, with elevations forming sectors, furrowed, without prominent exudates. On MEA, olivaceous grey, with pronounced radial grooves, slightly lighter outer ring, reverse olivaceous black, aerial mycelium, velvety, furrowed, without exudate. On OA, olivaceous grey, smooth texture, faint central ring, subtle radial pattern, reverse olivaceous black, aerial mycelium, smooth, without prominent exudates. Colonies average diameters (mm) of 42, 43, and 38 on CYA, DG18, and MY40G, respectively. Colony growth on PDA was observed at 30 °C, and no growth was observed at 36 °C.

##### Additional material examined.

BRAZIL • Goiás state, Mambaí municipality, Gruna da Tarimba cave, isol. from air, May 2023, coll. J.F.S.A. Prazeres, J.D.P. Bezerra & E.O. Fonseca, isol. E.O. Fonseca, isolate FCCUFG 146.

##### Notes.

*Cladosporium carsi* sp. nov. (*C. cladosporioides*SC) is morphologically (Table 3) and phylogenetically (Figs 3, 9) related to *C. proteacearum*. *Cladosporium carsi* differs morphologically from *C. proteacearum*, a species isolated from flower blight of *Macadamia integrifolia* in Australia (Prasannath et al. 2021), by having slightly smaller conidiophores (150–500 × 2.5–4 µm in *C. proteacearum*) that are erect, multiseptate, and have a subcylindrical and flexuous appearance. The primary ramoconidia are larger (12–48 × 3–5 µm) than those of *C. carsi* (8–27 × 2–4 µm). The intercalary conidia of *C. proteacearum* are smaller (4–5 × 2–3 µm) than those of *C. carsi* (4–12 × 2–4 µm) and have a different form. The closest phylogenetic species, *C. proteacearum* (BRIP 72301a), differs from *C. carsi* (URM 9208) by 3 bp in the *ACT* sequence and 9 bp in the *TEF1-α* sequence; the ITS sequence has no base pair difference. *RPB2* and *TUB* sequences were not obtained from *C. proteacearum* (BRIP 72301a) to compare with *C. carsi* (URM 9208), but the difference in bp of *C. carsi* is similar to that reported for the other analysed species. Additionally, the PHI test did not reveal evidence of genetic recombination (Fig. 6A). These isolates were found at a sampling point inside the cave.

#### Cladosporium chlamydosporiformans

Taxon classificationAnimaliaCladosporialesCladosporiaceae

C.M. Pereira & R.W. Barreto, (2024)

BBFE850C-811A-506F-8B2F-EAFC2C19F347

##### Typification.

KENYA • Eastern Province, Marsabit National Park, isol. from uredinia of *Hemileia vastatrix* on leaves of *Coffea arabica*, Jan. 2015, H.C. Evans (holotype VIC 47529, a dried metabolically inactive culture, ex-type culture COAD 2571).

##### Description and illustration.

See Pereira et al. (2024).

##### Material examined.

BRAZIL • Goiás state, Mambaí municipality, Lapa do Penhasco cave, isol. from air, May 2023, coll. J.F.S.A. Prazeres, E.O. Fonseca & J.D.P. Bezerra, isol. J.F.S.A. Prazeres (isolates FCCUFG 130, FCCUFG 74, FCCUFG 78, FCCUFG 75, and FCCUFG 77), Lapa do Córrego das Dores cave, isol. from air by E.O. Fonseca (isolates FCCUFG 133), and Gruna da Tarimba cave, isol. from air by E.O. Fonseca (isolates FCCUFG 108, FCCUFG 115, and FCCUFG 68); • Goiás state, Vila Propício municipality, from Lapa do Boqueirão cave, isol. from air, May 2022, coll. P.H. Félix-Oliveira & J.D.P. Bezerra, isol. P.H. Félix-Oliveira (isolate FCCUFG 70).

##### Notes.

*Cladosporium chlamydosporiformans* (*C. cladosporioides*SC) was proposed by Pereira et al. (2024) for a fungus found on uredinia of *Hemileia vastatrix* on leaves of *Coffea arabica* (*Rubiaceae*) in Kenya and has also been reported in Brazil and Ethiopia. In this study, 10 isolates from four caves formed a well-supported clade with the ex-type strain of *C. chlamydosporiformans* (Fig. 3). The cave environment represents a new habitat report for this species (found at sampling points both inside and outside the caves).

#### Cladosporium flabelliforme

Taxon classificationAnimaliaCladosporialesCladosporiaceae

Bensch, Summerell, Crous & U. Braun, (2010)

46727EB2-3EA9-59E5-AD6B-D980C5A2624A

##### Typification.

AUSTRALIA • Northern Territory, Foggy Dam, isol. from *Melaleuca cajuputi* (*Myrtaceae*), Sep. 2007, coll. B.A. Summerell, isol. P.W. Crous (holotype CBS H-20433, ex-type culture CBS 126345 = CPC 14523).

##### Description and illustration.

See Bensch et al. (2010).

##### Material examined.

BRAZIL • Goiás state, Mambaí municipality, Lapa do Penhasco cave, isol. from air, May 2023, coll. J.F.S.A. Prazeres, E.O. Fonseca & J.D.P. Bezerra, isol. J.F.S.A. Prazeres, isolate FCCUFG 201.

##### Notes.

*Cladosporium flabelliforme* (*C. cladosporioides*SC), proposed by Bensch et al. (2010), was initially isolated from *Melaleuca cajuputi* (*Myrtaceae*). This species has also been used for the optimisation and enhanced production of thrombolytic enzymes (Hariharan et al. 2023). In the present study, isolate FCCUFG 201 formed a well-supported clade with the ex-type strain of *C. flabelliforme* (Fig. 3). This is the first report of this species from a Brazilian cave (found at a sampling point inside the cave).

#### Cladosporium lacerdae

Taxon classificationAnimaliaCladosporialesCladosporiaceae

J.F.S.A. Prazeres, P.H. Félix-Oliveira, E.O. Fonseca, Souza-Motta & J.D.P. Bezerra sp. nov.

14A55AED-0802-5459-B434-6B55DF27DB3F

860818

Fig. 11

##### Etymology.

Named in honour of Dr. Heidi Lacerda of the Plataforma Multiusuários de Genômica e Transcriptômica do Centro de Biociências (MULTISEQ) of the Universidade Federal de Pernambuco (UFPE, Recife, Brazil) for her work at the sequencing facility for the last 15 years.

##### Typification.

BRAZIL • Goiás state, Mambaí, Lapa do Córrego das Dores, 14°25'45"S, 46°13'10"W, isol. from air, May 2023, coll. J.F.S.A. Prazeres, E.O. Fonseca & J.D.P. Bezerra, isol. E.O. Fonseca [**holotype**URM 9210HT (preserved in metabolically inactive state), UFG 39353 (microscopic slide ex-holotype), culture ex-type URM 9210 = FCCUFG 72].

**Figure 11.**
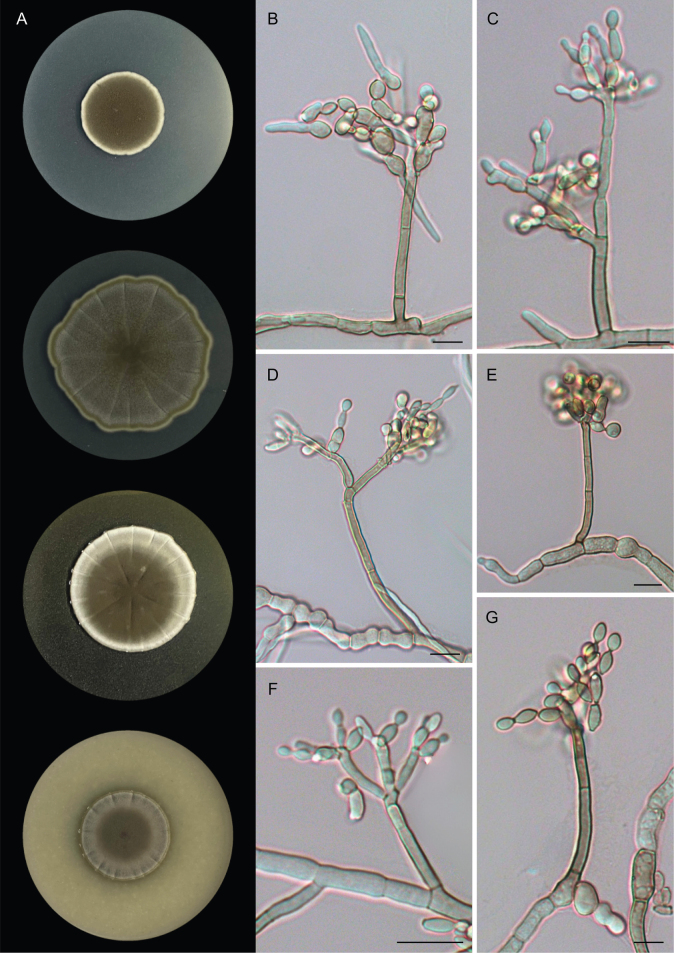
*Cladosporium lacerdae* (URM 9210, ex-type). **A**. Colonies (from top to bottom) on SNA, PDA, MEA, and OA after 14 days at 25 °C in the dark. **B–E**. Macronematous conidiophores, ramoconidia, conidiogenous cells, and conidial chains. **F**. Semimacronematous conidiophore. **G**. Macronematous conidiophore and conidial chains. Scale bars: 10 μm.

##### Description.

***Hyphae*** branched, septate, hyaline to dark brown, smooth, thin-walled. ***Conidiophores*** solitary, semimicronematous to macronematous, dark brown, arising terminally and laterally from hyphae, erect, cylindrical-oblong to filiform, non-nodular, usually geniculate, unbranched to branched, regularly septate, 90–250 × 2.5–4 μm. ***Conidiogenous cells*** integrated, cylindrical-oblong, terminal and intercalary, with 1–3 darkened apical conidiogenous loci, 6–27 × 2–4 μm. ***Ramoconidia*** often formed, subcylindrical, cylindrical-oblong to oblong, aseptate to septate, minutely verrucose, 10.5–18.5 × 2–4 μm. ***Secondary ramoconidia*** ellipsoid, fusiform to subcylindrical or cylindrical, aseptate, with 1–2 distal hila, light brown, smooth or almost smooth to minutely verrucose, walls not thickened, 8.5–24 × 2.5–4 μm. ***Intercalary conidia*** in branched chains, branching in all directions, aseptate, ovoid, ellipsoid, small fusiform, 9–14 × 2.5–4 μm; ***terminal conidia*** fusiform to ellipsoid, 2–6 × 2–3 μm.

##### Culture characteristics.

Colonies average diameters (mm) of 30, 54, 44, and 33 on SNA, PDA, MEA, and OA, respectively. On SNA, olivaceous, with a distinct light yellow margin, olivaceous black reverse, velvety aerial mycelium, without prominent exudates. On PDA, greenish olivaceous, with a distinct radial pattern and pronounced darker striations giving a segmented appearance, reverse olivaceous black, aerial mycelium, without prominent exudates. On MEA, grey olivaceous, outer ring lighter and whitish, reverse olivaceous black, aerial mycelium velvety, furrowed, without prominent exudates. On OA, pale olivaceous grey, smooth, with a faint central ring and less pronounced radial furrows, reverse olivaceous black, aerial mycelium smooth, without prominent exudates. Colonies average diameters (mm) of 53, 53, and 57 on CYA, DG18, and MY40G, respectively. Colony growth on PDA was observed at 30 °C, and no growth was observed at 36 °C.

##### Additional material examined.

BRAZIL • Goiás state, Mambaí municipality, Lapa do Córrego das Dores cave, isol. from air, May 2023, coll. J.F.S.A. Prazeres, E.O. Fonseca & J.D.P. Bezerra, isol. E.O. Fonseca, isolate FCCUFG 122.

##### Notes.

*Cladosporium lacerdae* sp. nov. (*C. cladosporioides*SC) is morphologically (Table 3) and phylogenetically (Figs 3, 9) related to *C. oxysporum*, a species described from dead leaves of *Passiflora* sp. in Cuba (Bensch et al. 2012). Microscopically, *C. lacerdae* has solitary conidiophores, ranging from semimicronematous to macronematous, erect, cylindrical-oblong, non-nodular, and regularly septate (90–250 × 2.5–4 μm). In contrast, *C. oxysporum* displays macronematous and occasionally micronematous conidiophores, erect and slightly flexuous, with distinct, spaced nodules, and measuring 40–720 × 2–4 μm. The conidiogenous cells of *C. lacerdae* are integrated, cylindrical-oblong, terminal, and intercalary, with 1–3 darkened apical conidiogenous loci (6–27 × 2–4 μm), whereas those of *C. oxysporum* are subdenticulate, with 1–4 loci per node, measuring 14–46 × 0.8–1.5 μm, often thickened and refractive. Ramoconidia are smaller and frequently formed in *C. lacerdae* (10.5–18.5 × 2–4 μm), whereas they are larger and occasionally observed in *C. oxysporum* (25–30 × 5–6 μm). Terminal conidia of *C. lacerdae* are ellipsoid to fusiform and measure 2–6 × 2–3 μm. Secondary ramoconidia are ellipsoid, fusiform to subcylindrical, or cylindrical, with 1–2 distal hila, light brown, smooth to minutely verrucose, and measure 8.5–24 × 2.5–4 μm. In contrast, *C. oxysporum* produces globose to subglobose terminal conidia, 3–5 × 2–3 μm, and intercalary conidia in branched chains. These intercalary conidia are ovoid to ellipsoid, occasionally small fusiform, measuring 9–14 × 2.5–4 μm, with 2–5 distal hila that are slightly thickened and refractive. The closest phylogenetic species, *C. oxysporum* (CBS 125991), differs from *C. lacerdae* (URM 9210) by 8 bp in *ACT*, 16 bp in *RPB2*, 8 bp in *TEF1-α*, and 7 bp in *TUB*; the ITS sequence has no base pair difference. For this species, the PHI test did not indicate evidence of genetic recombination (Fig. 6B). These isolates were found at a sampling point outside the cave.

#### Cladosporium macadamiae

Taxon classificationAnimaliaCladosporialesCladosporiaceae

Prasannath, Akinsanmi & R.G. Shivas, (2021)

0282CA3B-3141-5E2E-BC46-5D9AA2C50826

##### Typification.

AUSTRALIA • Queensland, Nambour, isol. from flower blight of *Macadamia integrifolia* (*Proteaceae*), Sep. 2019, O.A. Akinsanmi (holotype BRIP 72269a, includes ex-type culture).

##### Description and illustration.

See Prasannath et al. (2021).

##### Material examined.

BRAZIL • Goiás state, Mambaí municipality, Lapa do Penhasco cave, isol. from soil, May 2023, coll. J.F.S.A. Prazeres, E.O. Fonseca & J.D.P. Bezerra, isol. J.F.S.A. Prazeres (isolate FCCUFG 67), Lapa do Córrego das Dores cave, isol. from air by E.O. Fonseca (isolate FCCUFG 116), and Lapa da Cachoeira do Funil cave, isol. from air by E.O. Fonseca (isolate FCCUFG 135).

##### Notes.

*Cladosporium macadamiae* (*C. cladosporioides*SC) was introduced by Prasannath et al. (2021) from isolates associated with grey and green mould symptoms on macadamia flowers in Australia. In the present study, three isolates obtained from the air and soil of three caves formed a well-supported clade with the ex-type strain of *C. macadamiae* (Figs 3, 9), constituting the first report of this species from a Brazilian cave (found at sampling points both inside and outside the caves).

#### Cladosporium mambaiense

Taxon classificationAnimaliaCladosporialesCladosporiaceae

J.F.S.A. Prazeres, P.H. Félix-Oliveira, E.O. Fonseca, Souza-Motta & J.D.P. Bezerra sp. nov.

B04F37F4-9083-576F-BEA6-D1C915C772B7

860819

Fig. 12

##### Etymology.

Named after Mambaí, the municipality where most of the caves studied here are located.

##### Typification.

BRAZIL • Goiás state, Mambaí municipality, Lapa do Córrego das Dores cave, 14°25'45"S, 46°13'10"W, isol. from air, May 2023, coll. J.F.S.A. Prazeres, E.O. Fonseca & J.D.P. Bezerra, isol. E.O. Fonseca [**holotype**URM 9104HT (preserved in metabolically inactive state), UFG 39351 (microscopic slide ex-holotype), culture ex-type URM 9104 = FCCUFG 71].

**Figure 12.**
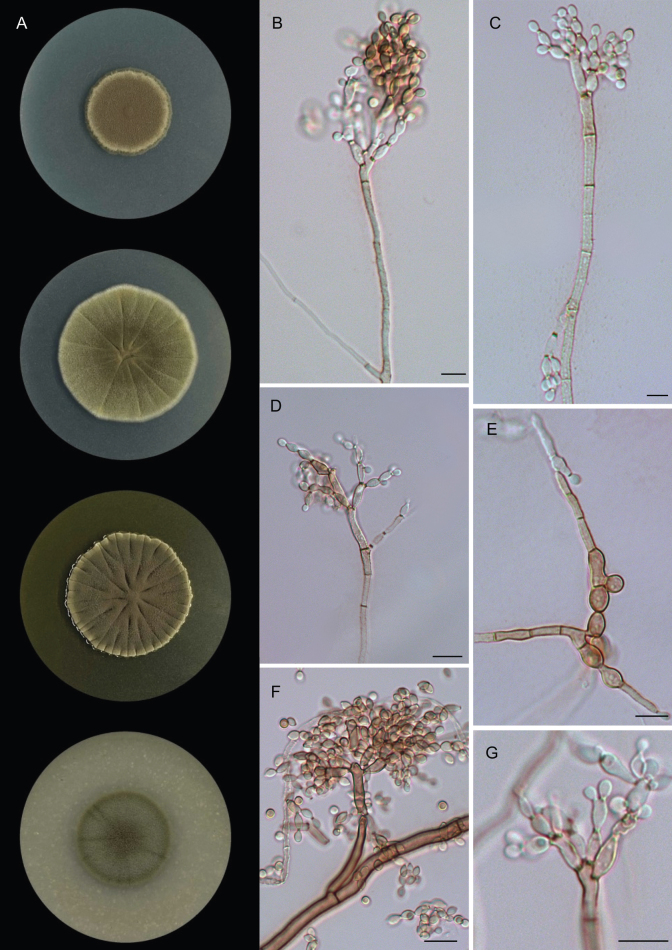
*Cladosporium mambaiense* (URM 9104, ex-type). **A**. Colonies (from top to bottom) on SNA, PDA, MEA, and OA after 14 days at 25 °C in the dark. **B, C**. Macronematous conidiophores, ramoconidia, conidiogenous cells, and conidia. **D**. Ramoconidia and conidial chain. **E**. Details of a germinating conidial chain. **F**. Semimacronematous conidiophore, ramoconidia, conidiogenous cells, and conidia. **G**. Details of ramoconidia and conidia. Scale bars: 10 μm.

##### Description.

***Hyphae*** branched, septate, hyaline to light brown, smooth, thick-walled. ***Conidiophores*** solitary, semimacronematous to macronematous, branched, hyaline to light brown, arising terminally and laterally from hyphae, erect to flexuous, often filiform, non-nodular, regularly septate, septa often darkened and thick, 110–554 × 2.5–4 μm. ***Conidiogenous cells*** integrated, cylindrical-oblong, terminal and intercalary, with 1–4 darkened apical loci, 7–29 × 3–5 μm. ***Ramoconidia*** often formed, subcylindrical, cylindrical-oblong to oblong, aseptate, smooth, 16.5–25 × 2–5 μm. ***Secondary ramoconidia*** ellipsoid, fusiform to subcylindrical or cylindrical, aseptate, hyaline to light brown, smooth or minutely verrucose, with slightly thickened walls, 11–30 × 2–3.5 μm. ***Intercalary conidia*** in branched chains, branching in all directions, without regular septation, ovoid, ellipsoid, small fusiform, 7–13 × 2.5–4 μm; ***terminal conidia*** small, fusiform to ellipsoid, 3–5 × 1–3 μm.

##### Culture characteristics.

Colonies average diameters (mm) of 29, 44, 40, and 37 on SNA, PDA, MEA, and OA, respectively. On SNA, the colonies are greenish olivaceous, with a smooth, compact centre and a lighter, well-defined edge, reverse olivaceous black, smooth, compact aerial mycelium, without prominent exudates. On PDA, olivaceous buff, velvety texture, distinct radial groove pattern, reverse olivaceous black, smooth aerial mycelium, without prominent exudates. On MEA, olivaceous grey, with pronounced radial furrows and a slightly lighter outer ring, reverse olivaceous black, compact, smooth aerial mycelium, without prominent exudates. On OA, grey olivaceous, with a smooth texture, faint central ring and a subtle radial pattern, reverse olivaceous black, smooth aerial mycelium, without prominent exudates. Colonies average diameters (mm) of 44, 50, and 47 on CYA, DG18, and MY40G, respectively. Colony growth on PDA was observed at 30 °C, and no growth was observed at 36 °C.

##### Additional material examined.

BRAZIL • Goiás state, Vila Propício municipality, Lapa do Boqueirão cave, isol. from air, May 2022, coll. P.H. Félix-Oliveira & J.D.P. Bezerra, isol. P.H. Félix-Oliveira (isolate FCCUFG 147).

##### Notes.

*Cladosporium mambaiense* sp. nov. (*C. cladosporioides*SC) is morphologically (Table 3) and phylogenetically (Figs 3, 9) related to *C. angustisporum*. *Cladosporium mambaiense* differs morphologically from *C. angustisporum*, a species described from *Alloxylon wickhamii* (*Proteaceae*) in Australia (Bensch et al. 2012), mainly by the size and shape of its reproductive structures. *Cladosporium mambaiense* has longer (110–554 × 2.5–4 μm) and erect conidiophores, whereas the conidiophores of *C. angustisporum* are smaller (22–280 × 2.5–4 μm). Ramoconidia of *C. mambaiense* are subcylindrical, smooth, and smaller (16.5–25 × 2–5 μm), whereas those of *C. angustisporum* are cylindrical and measure 18–55 × 2.5–3 μm. The closest phylogenetic species, *C. angustisporum* (CBS 125983), differs from *C. mambaiense* (URM 9104) by 12 bp in *ACT*, 14 bp in *TEF1-α*, and 15 bp in *TUB*; the ITS sequence shows no base pair difference. The *RPB2* sequence was not obtained from the ex-type strain of *C. angustisporum* (CBS 125983), but it was obtained from *C. angustisporum* (CPC 22345), also included in the analysis, and a 32 bp difference was observed. The PHI test did not indicate evidence of genetic recombination (Fig. 6C). These isolates were found at sampling points inside the caves.

#### Cladosporium mesquitapaivae

Taxon classificationAnimaliaCladosporialesCladosporiaceae

J.F.S.A. Prazeres, P.H. Félix-Oliveira, E.O. Fonseca, Souza-Motta & J.D.P. Bezerra sp. nov.

EEFBA511-9D32-59D4-A896-AE0773CB38D4

860820

Fig. 13

##### Etymology.

Named in honour of Prof. Dr. Laura Mesquita Paiva of the Departamento de Micologia at the Universidade Federal de Pernambuco (UFPE, Recife, Brazil) for her contribution to the academic study of fungi.

##### Typification.

BRAZIL • Goiás state, Mambaí municipality, Lapa do Córrego das Dores cave, 14°25'45"S, 46°13'10"W, isol. from air, May 2023, coll. J.F.S.A. Prazeres, E.O. Fonseca & J.D.P. Bezerra, isol. E.O. Fonseca [**holotype**URM 9103HT (preserved in metabolically inactive state), UFG 39348 (microscopic slide ex-holotype), culture ex-type URM 9103 = FCCUFG 65].

**Figure 13.**
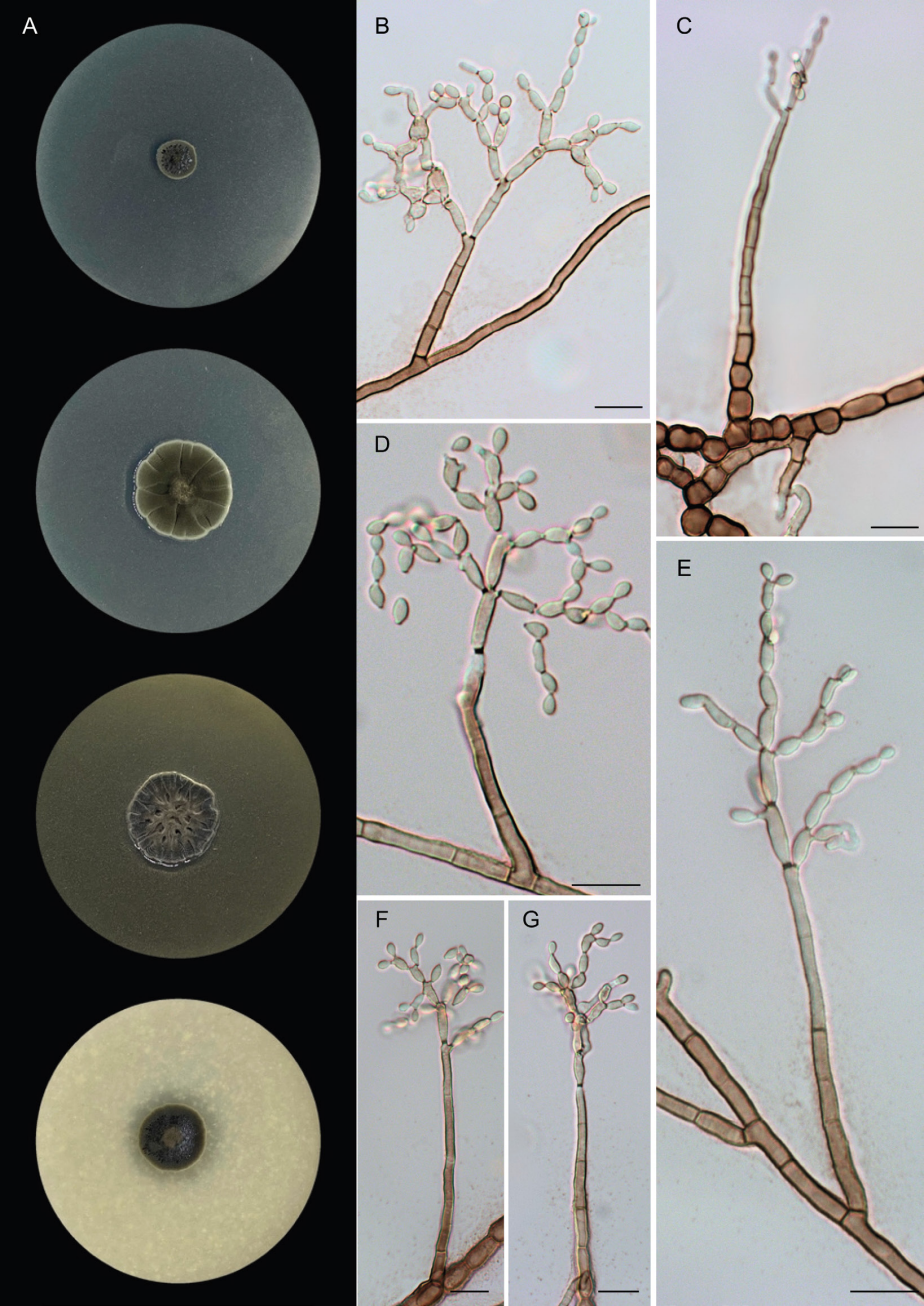
*Cladosporium mesquitapaivae* (URM 9103, ex-type). **A**. Colonies (from top to bottom) on SNA, PDA, MEA, and OA after 14 days at 25 °C in the dark. **B–E**. Macronematous conidiophores, ramoconidia, conidiogenous cells, and conidial chains. Scale bars: 10 μm.

##### Description.

***Hyphae*** branched, septate, olive-brown, smooth, thick-walled. ***Conidiophores*** solitary, macronematous, arising terminally and laterally from hyphae, usually formed by stromatic hyphae, erect, straight to slightly flexuous, cylindrical-oblong to filiform, commonly geniculate, unbranched to branched, regularly septate, septa often darkened, conidiogenous locus at the apex, 27–90 × 2.5–4 μm. ***Conidiogenous cells*** integrated, terminal and intercalary, cylindrical-oblong, long, with 1-3 thickened blackish apical conidiogenous loci, 4–17 × 1–3 μm. ***Ramoconidia*** often formed, subcylindrical to cylindrical-oblong, usually aseptate, 8–15.5 × 2–3.5 μm. ***Secondary ramoconidia*** ellipsoid, fusiform to subcylindrical or cylindrical, aseptate, with 1–2 distal hila, hyaline to light brown, smooth, walls not thickened, 8–11 × 2–3.5 μm. ***Intercalary conidia*** in branched chains, branching in all directions, aseptate, ovoid, ellipsoid, small fusiform 3–7 × 2–4 μm; ***terminal conidia*** fusiform to ellipsoid 2–3 × 1.5 μm.

##### Culture characteristics.

Colonies average diameters (mm) of 10, 26, 23, and 17 on SNA, PDA, MEA, and OA, respectively. On SNA, violaceous black, with some parts of the mycelium shiny (stromatic hyphae), generally flat growth with elevation in the centre of the colony, diffuse with sectors, light grey margins, olivaceous black reverse, compact aerial mycelium, smooth, without prominent exudates. On PDA, violaceous black with a dense, furrowed centre and smooth margins, reverse olivaceous black, smooth aerial mycelium, without prominent exudates. On MEA, violaceous black, with a defined central zone and lighter outer margins, reverse olivaceous black, velvety smooth aerial mycelium, without prominent exudates. On OA, violaceous black with some parts of the mycelium shiny (stromatic hyphae), flat growth with elevation in the colony’s centre, olivaceous green margins, reverse olivaceous black, smooth aerial mycelium, without prominent exudates. Colonies average diameters (mm) of 35, 40, and 27 on CYA, DG18, and MY40G, respectively. Colony growth on PDA was observed at 30 °C, and no growth was observed at 36 °C.

##### Additional material examined.

BRAZIL • Goiás state, Mambaí municipality, Lapa do Córrego das Dores cave, isol. from air, May 2023, coll. J.F.S.A. Prazeres, E.O. Fonseca & J.D.P. Bezerra, isol. E.O. Fonseca, isolate FCCUFG 136.

##### Notes.

*Cladosporium mesquitapaivae* sp. nov. (*C. sphaerospermum*SC) is morphologically (Table 3) and phylogenetically (Figs 4, 9) related to a clade with *C. aciculare* and *C. fusiforme*. *Cladosporium mesquitapaivae* differs from *C. aciculare*, a species described from *Syzygium corynanthum* (*Myrtaceae*) in Australia (Bensch et al. 2015), by having smaller conidiophores (27–90 × 2.5–4 μm), ramoconidia (8–15.5 × 2–3.5 μm), and intercalary conidia (3–7 × 2–3 μm). *Cladosporium fusiforme* was described from hypersaline water in Slovenia (Zalar et al. 2007) and is differentiated from *C. mesquitapaivae* by its larger conidiophores (10–100 × 2–4 μm) and secondary ramoconidia (5–23 × 2–3 μm). The closest phylogenetic species, *C. aciculare* (CBS 140488), differs from *C. mesquitapaivae* (URM 9103) by 17 bp in *ACT*, 14 bp in ITS, 39 bp in *TEF1-α*, and 29 bp in *TUB*, and *C. fusiforme* (CBS 119414) differs from *C. mesquitapaivae* (URM 9103) by 25 bp in *ACT*, 8 bp in ITS, and 39 bp in *TEF1-α*. The *RPB2* sequence was not obtained from *C. mesquitapaivae* (URM 9103). The PHI test did not indicate evidence of genetic recombination between *C. aciculare* and *C. mesquitapaivae* (Fig. 8B) or between *C. fusiforme* and *C. mesquitapaivae* (data not shown). These isolates were found at sampling points inside the cave.

#### Cladosporium nogueirae

Taxon classificationAnimaliaCladosporialesCladosporiaceae

P.H. Félix-Oliveira, J.F.S.A. Prazeres, Souza-Motta & J.D.P. Bezerra sp. nov.

40880F09-5A80-5142-9641-006D4086F5E3

860821

Fig. 14

##### Etymology.

Named in honour of Prof. Dr. Ina de Souza Nogueira of the Departamento de Biologia Geral at the Universidade Federal de Goiás (UFG, Goiânia, Brazil) for her contribution to the academic study of fungi in the Goiás state.

**Figure 14.**
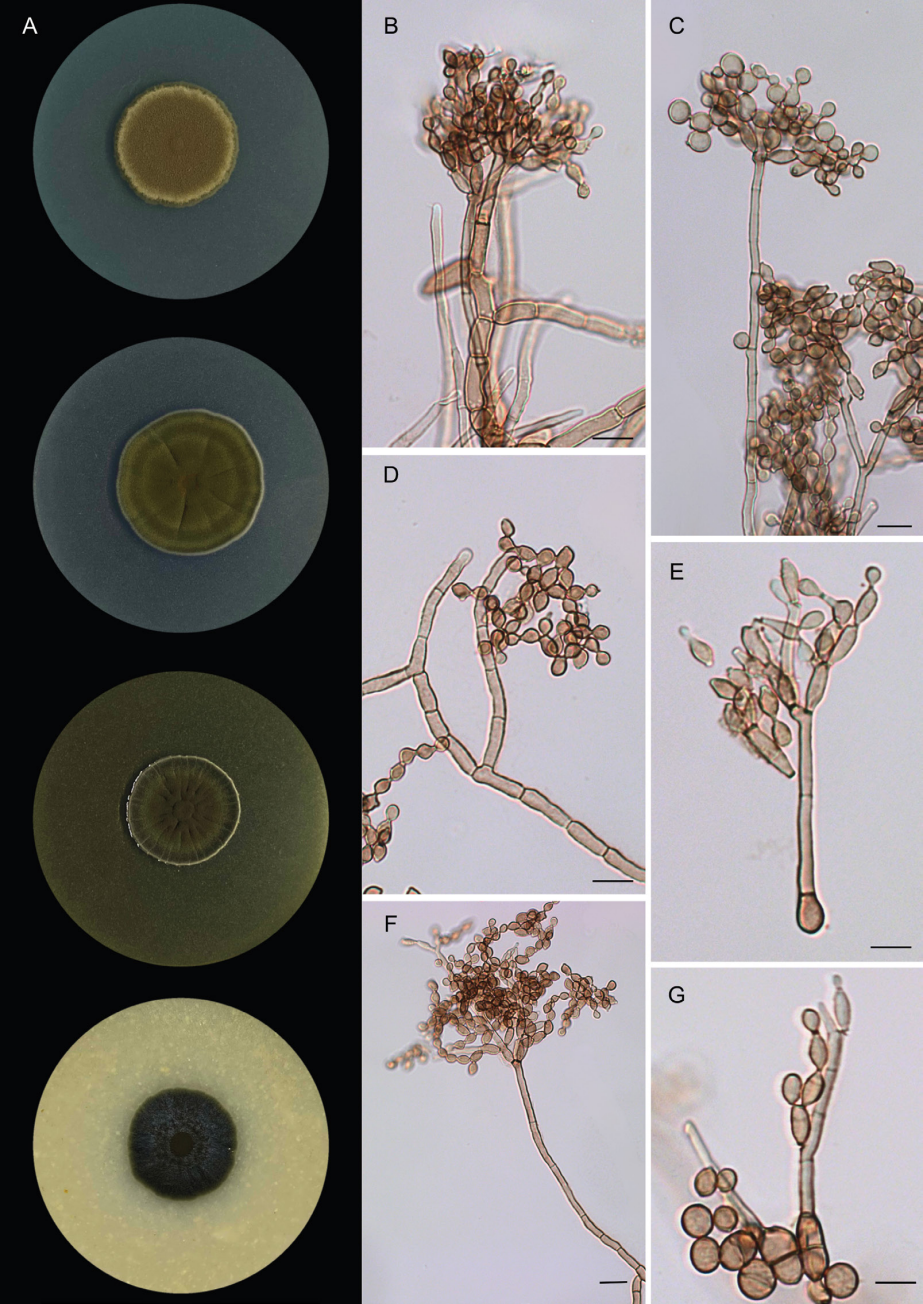
*Cladosporium nogueirae* (URM 9209, ex-type). **A**. Colonies (from top to bottom) on SNA, PDA, MEA, and OA after 14 days at 25 °C in the dark. **B–D**. Macronematous conidiophores, ramoconidia, conidiogenous cells, and conidial chains. **E**. Details of a secondary ramoconidium forming a conidiophore. **F**. Macronematous conidiophore and conidial chains. **G**. Details of a germinating conidium forming a conidiophore. Scale bars: 10 μm.

##### Typification.

BRAZIL • Goiás state, Mambaí municipality, Lapa do Córrego das Dores cave, 14°25'45"S, 46°13'10"W, isol. from air, May 2023, coll. J.F.S.A. Prazeres, E.O. Fonseca & J.D.P. Bezerra, isol. E.O. Fonseca [holotype URM 9209HT (preserved in metabolically inactive state), UFG 39352 (microscopic slide ex-holotype), culture ex-type URM 9209 = FCCUFG 61].

##### Description.

***Hyphae*** branched, septate, dark brown, smooth, thickened walls. ***Conidiophores*** solitary, macronematous, arising terminally and laterally from hyphae, erect to slightly flexuous, cylindrical-oblong to filiform, neither nodular nor geniculate, unbranched to branched, regularly septate, septa often darkened, 108–170 × 2.5–3.5 μm. ***Conidiogenous cells*** integrated, cylindrical-oblong, terminal and intercalary, with 1–2 darkened apical conidiogenous loci, 5–11 × 1–3 μm. ***Ramoconidia*** often formed, rarely septate, subcylindrical to cylindrical-oblong, minutely verruculose, 11–22 × 2–5 μm. ***Secondary ramoconidia*** ellipsoid, fusiform to subcylindrical or cylindrical, rarely septate, with 1–3 distal hila, light brown, smooth to minutely verrucose, walls not thickened or occasionally slightly thickened, 5.5–12 × 1–3 μm. ***Intercalary conidia*** in branched chains, rarely septate, ovoid, ellipsoid, fusiform, 6–8 × 2–6 μm; ***terminal conidia*** fusiform to ellipsoid, 4–7 × 1–3 μm.

##### Culture characteristics.

Colonies average diameters (mm) of 22, 37, 27, and 29 on SNA, PDA, MEA, and OA, respectively. On SNA, olivaceous with a dark centre and a lighter outer zone, reverse olivaceous black. Aerial mycelium, smooth, compact, without prominent exudates. On PDA, greenish olivaceous with a distinct radial pattern in the centre, reverse olivaceous black. Aerial mycelium, smooth with slight furrowing, without prominent exudates. On MEA, grey olivaceous with central furrowing and a defined lighter edge, reverse olivaceous black. Aerial mycelium, slightly furrowed, without prominent exudates. On OA, pale olivaceous with a darker centre and smooth texture, reverse olivaceous black. Aerial mycelium, smooth, without prominent exudates. Colonies average diameters (mm) of 35, 38, and 17 on CYA, DG18, and MY40G, respectively. Colony growth on PDA was observed at 30 °C, and no growth was observed at 36 °C.

##### Additional material examined.

BRAZIL • Goiás state, Vila Propício municipality, Lapa do Boqueirão cave, isol. from air, May 2022, coll. J.D.P. Bezerra & P.H. Félix-Oliveira, isol. P.H. Félix-Oliveira, isolates FCCUFG 137, and FCCUFG 125.

##### Notes.

*Cladosporium nogueirae* sp. nov. (*C. cladosporioides*SC) is morphologically (Table 3) and phylogenetically (Figs 3, 9) related to *C. rugulovarians* and *C. propiciense* sp. nov. (this study), a clade having only species from Brazil. *Cladosporium nogueirae* and *C. rugulovarians* can be differentiated by several morphological characteristics, including the size and shape of their structures. The conidiophores of *C. rugulovarians* are distinctly longer (up to 475 µm), geniculate, and dichotomously branched, in contrast to those of *C. nogueirae*, which are not geniculate and measure 108–170 × 2.5–3.5 μm. The conidiogenous cells are also larger in *C. rugulovarians* (up to 60 µm long) than in *C. nogueirae* (5–11 × 1–3 μm). The ramoconidia of *C. rugulovarians* (20–55 × 3–4.5 μm) are larger than those of *C. nogueirae* (11–22 × 2–5 μm). Similarly, the secondary ramoconidia of *C. rugulovarians* [(9–)11–24(–30) × (2–)2.5–5.5 μm] are larger than those of *C. nogueirae* (5.5–12 × 1–3 μm). The terminal conidia also differ in shape and size, being fusiform to ellipsoid and measuring 4–7 × 1–3 μm in *C. nogueirae*, and globose to subglobose, measuring 3–6.5 × 3–5 μm in *C. rugulovarians*. The morphological differentiation between *C. nogueirae* (URM 9209) and *C. propiciense* (URM 9353) is highlighted below. The closest phylogenetic species, *C. rugulovarians* (CBS 140495), differs from *C. nogueirae* (URM 9209) by 4 bp in *ACT*, 16 bp in *RPB2*, 5 bp in *TEF1-α*, and 6 bp in *TUB*, and the differences in bp from *C. propiciense* (URM 9353) are described below; ITS has no bp difference. The PHI test did not indicate evidence of genetic recombination (Fig. 7A, B). These isolates were found at sampling points both inside and outside the caves.

#### Cladosporium perangustum

Taxon classificationAnimaliaCladosporialesCladosporiaceae

Bensch, Crous & U. Braun, (2010)

707206AC-081F-5572-B5F8-E3B11576F2F2

##### Typification.

SOUTH AFRICA • isol. from *Cussonia* sp. (*Araliaceae*), Mar. 2007, P.W. Crous (holotype CBS H-20451, ex-type culture CBS 125996 = CPC 13815).

##### Description and illustration.

See Bensch et al. (2010).

##### Material examined.

BRAZIL • Goiás state, Vila Propício municipality, Garganta (Samambaia) cave, isol. from air, Sep. 2022, coll. P.H. Félix-Oliveira, J.D.P. Bezerra & R.F.F. Franco, isol. P.H. Félix-Oliveira, isolate FCCUFG 200.

##### Notes.

*Cladosporium perangustum* (*C. cladosporioides*SC) is a widely distributed species found on plant material, on other ascomycetes, and isolated from food, with reports from Africa (South Africa), Asia (India, Thailand), Australasia (Australia, New Zealand, Polynesia), Europe (Germany), and North America (USA) (Bensch et al. 2010, 2012, 2018). The isolate obtained from the air formed a well-supported clade with the ex-type strain of *C. perangustum* (Suppl. material 1). The present study expands knowledge of this species’ ecology, constituting the first report of this species in a Brazilian cave (found at a sampling point inside the cave.

#### Cladosporium pernambucoense

Taxon classificationAnimaliaCladosporialesCladosporiaceae

M.L.S. Pereira, J.D.P. Bezerra & Souza-Motta, (2022)

7227B45E-2C58-559B-B130-19575C98D9F3

##### Typification.

BRAZIL • Pernambuco state, Ibimirim municipality, Catimbau National Park, Furna do Morcego bat cave, isol. from air, Oct. 2019, M.L.S. Pereira (holotype URM 94479, ex-type culture URM 8390 = Isolate X7).

##### Description and illustration.

See Pereira et al. (2022).

##### Material examined.

BRAZIL • Goiás state, Vila Propício municipality, Lapa do Boqueirão cave, isol. from soil, May 2022, coll. P.H. Félix-Oliveira & J.D.P. Bezerra, isol. P.H. Félix-Oliveira, isolate JB791.

##### Notes.

*Cladosporium pernambucoense* (*C. cladosporioides*SC) was first isolated from the air in a Caatinga cave in Brazil (Pereira et al. 2022). In the present study, it was isolated from soil samples in Cerrado caves, expanding its geographical distribution in Brazil. To date, this species has been reported exclusively from caves, and this work represents the second report of this species (found at a sampling point inside the cave).

#### Cladosporium propiciense

Taxon classificationAnimaliaCladosporialesCladosporiaceae

P.H. Félix-Oliveira, J.F.S.A. Prazeres, Souza-Motta & J.D.P. Bezerra sp. nov.

144EF458-D5AF-5BA3-979D-C9C8826A3292

860822

Fig. 15

##### Etymology.

The name is in reference to the municipality of Vila Propício, where the fungus was collected from a cave.

##### Typification.

BRAZIL • Goiás state, Vila Propício municipality, Garganta (Samambaia) cave, 15°22'54"S, 48°41'55"W, isol. from air, Sep. 2022, coll. P.H. Félix-Oliveira, J.D.P. Bezerra & R.F.F. Franco, isol. P.H. Félix-Oliveira [holotype URM 9353HT (preserved in a metabolically inactive state), UFG 39350 (microscopic slide ex-holotype), culture ex-type URM 9353 = FCCUFG 76].

##### Description.

***Hyphae*** branched, septate, dark brown, smooth, thick-walled. ***Conidiophores*** solitary, macronematous, arising terminally and laterally from hyphae, erect to slightly flexuous, cylindrical-oblong to filiform, neither nodular nor geniculate, unbranched to branched, regularly septate, septa often darkened, 90–420 × 2.5–3.5 μm. ***Conidiogenous cells*** integrated, cylindrical-oblong, terminal and intercalary, with 1–3 darkened apical conidiogenous loci, 5–11 × 1–3 μm. ***Ramoconidia*** frequently formed, transversely and horizontally septate, subcylindrical, cylindrical-oblong and oblong, 10–27 × 2–5 μm. ***Secondary ramoconidia*** ellipsoid, fusiform to subcylindrical or cylindrical, septate, with 1–4 distal hila, light brown, smooth to minutely verrucose, walls not thickened or occasionally slightly thickened, 4–16 × 1–3 μm. ***Intercalary conidia*** in branched chains, rarely septate, ovoid, ellipsoid, fusiform, 6–9 × 2–6 μm; ***terminal conidia*** fusiform to ellipsoid, 4–7 × 1–3 μm.

**Figure 15.**
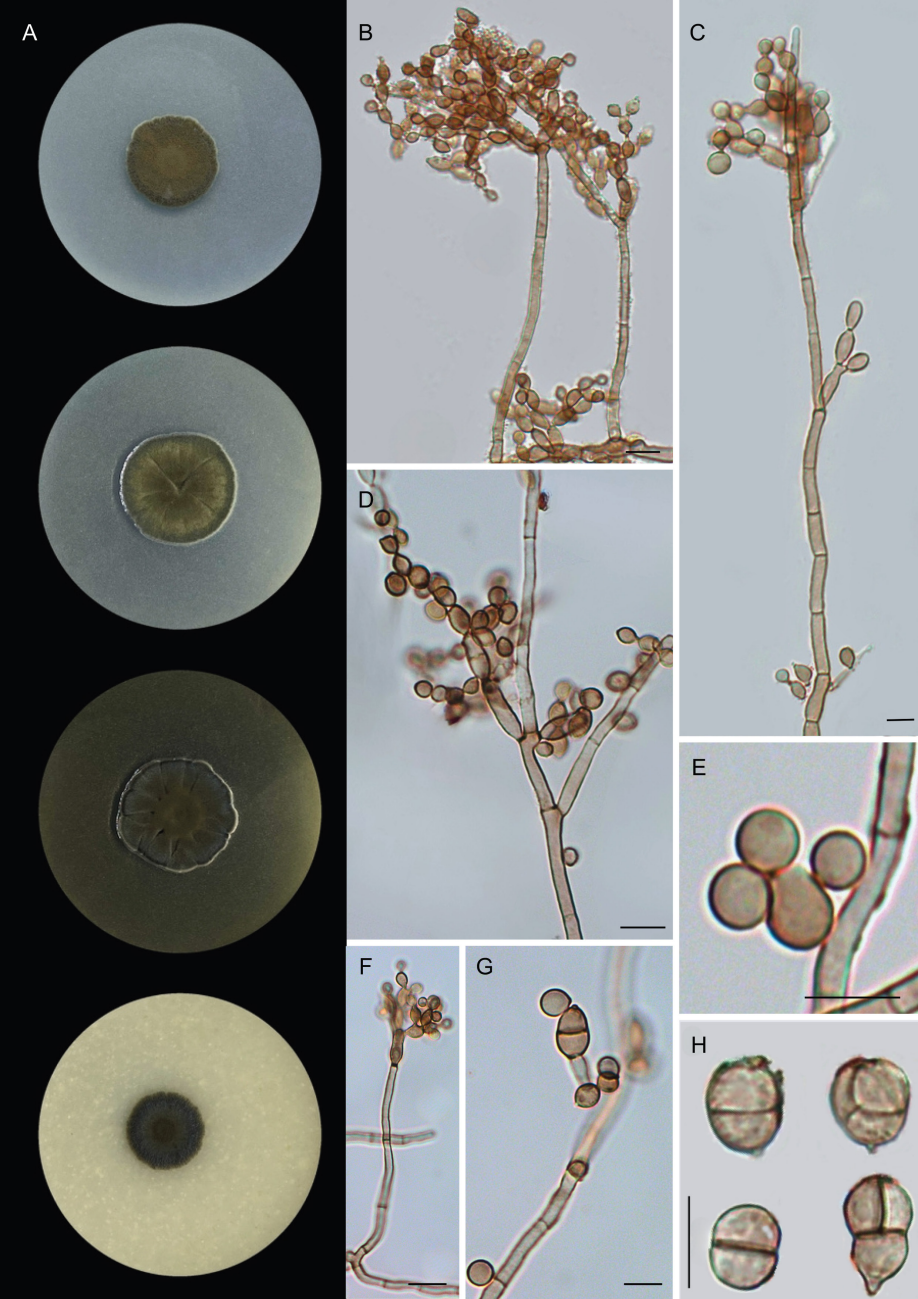
*Cladosporium propiciense* (URM 9353, ex-type). **A**. Colonies (from top to bottom) on SNA, PDA, MEA, and OA after 14 days at 25 °C in the dark. **B–D**. Macronematous conidiophores with conidial chains. **E**. Ramoconidia and globose conidia. **F**. Macronematous conidiophores with conidial chains. **G**. Details of septate ramoconidia and conidia. **H**. Details of ramoconidia. Scale bars: 10 μm.

##### Culture characteristics.

Colonies average diameters (mm) of 23, 31, 31, and 20 on SNA, PDA, MEA, and OA, respectively. On SNA, olivaceous, with a darker centre and a lighter, diffuse outer edge, reverse dark olivaceous. Aerial mycelium, low, velvety, without exudates. On PDA, greenish olivaceous to olivaceous with well-defined radial furrows, reverse dark olivaceous. Aerial mycelium, compact and velvety with radial furrows, without exudates. On MEA, dark mouse grey, elevated centre, reverse dark olivaceous to almost black. Aerial mycelium, dense, velvety, with some central folds. On OA, dark mouse grey and uniform olivaceous with a smooth and slightly velvety texture, reverse dark olivaceous. Aerial mycelium, low, compact, without exudates. Colonies average diameters (mm) of 29, 38, and 24 on CYA, DG18, and MY40G, respectively. Colony growth on PDA was observed at 30 °C, and no growth was observed at 36 °C.

##### Additional material examined.

BRAZIL • Goiás state, Vila Propício municipality, Garganta (Samambaia) cave, isol. from air, Sep. 2022, coll. P.H. Félix-Oliveira, J.D.P. Bezerra & R.F.F. Franco, isol. P.H. Félix-Oliveira, isolate FCCUFG 123.

##### Notes.

*Cladosporium propiciense* sp. nov. (*C. cladosporioides*SC) is morphologically (Table 3) and phylogenetically (Figs 3, 9) related to *C. nogueirae* sp. nov. (this study) and *C. rugulovarians*, a clade having only species described from Brazilian materials. *Cladosporium propiciense* can be differentiated mainly based on the size and septation of its reproductive structures. *Cladosporium propiciense* produces longer conidiophores (90–420 × 2.5–3.5 μm), whereas the conidiophores of *C. nogueirae* are smaller (108–170 × 2.5–3.5 μm). The ramoconidia of *C. propiciense* are often septate and measure 10–27 × 2–5 μm, whereas those of *C. nogueirae* are rarely septate and slightly smaller (11–22 × 2–5 μm). The secondary ramoconidia are often septate in *C. propiciense* but rarely septate in *C. nogueirae*. The conidiophores of *C. rugulovarians* are distinctly longer (up to 475 µm), geniculate, and dichotomously branched, in contrast to those of *C. propiciense*, which are not geniculate and measure 90–420 × 2.5–3.5 μm. The conidiogenous cells are also larger in *C. rugulovarians* (up to 60 µm long) than in *C. propiciense* (5–11 × 1–3 μm). The ramoconidia of *C. rugulovarians* (20–55 × 3–4.5 μm) are larger than those of *C. propiciense* (10–27 × 2–5 μm). Similarly, the secondary ramoconidia of *C. rugulovarians* [(9–)11–24(–30) × (2–)2.5–5.5 μm] are larger than those of *C. propiciense* (4–16 × 1–3 μm). The terminal conidia also differ in shape and size, being fusiform to ellipsoid and measuring 4–7 × 1–3 μm in *C. propiciense*, and globose to subglobose, measuring 3–6.5 × 3–5 μm in *C. rugulovarians*. The phylogenetically closest species, *C. nogueirae* (URM 9209), differs from *C. propiciense* (URM 9353) by 3 bp in *ACT*, 11 bp in *RPB2*, 3 bp in *TEF1-α*, and 6 bp in *TUB*, and no differences were observed for ITS; *C. rugulovarians* (CBS 140495) differs from *C. propiciense* by 1 bp in *ACT*, 12 bp in *RPB2*, and 2 bp in *TEF1-α*, and no differences were observed for ITS and *TUB.* The PHI test did not indicate evidence of genetic recombination (Fig. 7B, C). These isolates were found at sampling points outside the cave.

#### Cladosporium pruni-salicinae

Taxon classificationAnimaliaCladosporialesCladosporiaceae

Y.Q. Yang & Yong Wang bis [as ‘ pruni-salicina’], (2023)

37F143E5-324E-5C39-8042-89F732DB5D1B

##### Typification.

CHINA • Guizhou Province, Kaiyang County, isol. from leaves of *Prunus salicina*, Jun. 2021, Y.Q. Yang (holotype HGUP 21206; ex-type culture GUCC 21206.1).

##### Description and illustration.

Yang et al. (2023).

##### Material examined.

BRAZIL • Goiás state, Mambaí municipality, Gruna da Tarimba cave, isol. from air, May 2023, coll. J.F.S.A. Prazeres, E.O. Fonseca & J.D.P. Bezerra, isol. E.O. Fonseca, isolate FCCUFG 198.

##### Notes.

*Cladosporium pruni-salicinae* (*C. cladosporioides*SC) was initially isolated from leaves of *Prunus salicina* (*Rosaceae*) (Yang et al. 2023). In the present study, one isolate obtained from the air of a cave formed a well-supported lineage with the ex-type strain of *C. pruni-salicinae* (Suppl. material 1), constituting the first report of this species from a Brazilian cave (found at a sampling point inside the cave).

#### Cladosporium pseudocladosporioides

Taxon classificationAnimaliaCladosporialesCladosporiaceae

Bensch, Crous & U. Braun, (2010)

4DE5C6B0-35C4-539E-88CF-EC7D553C7487

##### Typification.

NETHERLANDS • Zwolle, isol. from outside air, 7 Jan. 2007, M. Meijer (holotype CBS H-20445, ex-type cultures CBS 125993 = CPC 14189, CPC 14193).

##### Description and illustration.

Bensch et al. (2010).

##### Material examined.

BRAZIL • Goiás state, Mambaí municipality, Gruna da Tarimba cave, isol. from air, May 2023, coll. J.F.S.A. Prazeres, E.O. Fonseca & J.D.P. Bezerra, isol. J.F.S.A. Prazeres (isolate FCCUFG 199), Lapa do Córrego das Dores cave, isol. from air by E.O. Fonseca (isolate FCCUFG 203).

##### Notes.

*Cladosporium pseudocladosporioides* (*C. cladosporioides*SC) is a widely distributed species that has been isolated from diverse substrates, including plant material, fungal sporocarps, air, soil, water, and food (Bensch et al. 2010), and has also been associated with raspberry fruit rot in the province of Quebec (Delisle-Houde et al. 2024). The two isolates obtained from air formed a well-supported clade with the ex-type strain of *C. pseudocladosporioides* (Suppl. material 1). The present study expands the known habitats of this species, constituting the first report in a Brazilian cave (found at a sampling point outside the caves).

#### Cladosporium puris

Taxon classificationAnimaliaCladosporialesCladosporiaceae

M.L.R. Freitas & O.L. Pereira, (2021)

EDA86140-D41D-5929-A654-C093F7632434

##### Synonym.

*Cladosporium brigadeirense* P.P. Costa, A.W.C. Rosado & O.L. Pereira, Phytotaxa 560: 19 (2022), syn. nov.

##### Typification.

BRAZIL • Minas Gerais state, Canaã, on submerged litter in streams, Sep. 2017 (holotype VIC 44468, ex-type culture COAD 2487).

##### Description and illustration.

See Freitas et al. (2021).

##### Materials examined.

BRAZIL • Goiás state, Vila Propício municipality, Lapa do Boqueirão cave, isol. from air, May 2022, coll. P.H. Félix-Oliveira & J.D.P. Bezerra, isol. P.H. Félix-Oliveira, isolate FCCUFG 145.

##### Notes.

*Cladosporium puris* (*C. cladosporioides*SC) was described by Freitas et al. (2021) from submerged leaves in streams in Brazil. Subsequently, *C. brigadeirense* was proposed by Costa et al. (2022) as a sister species to *C. puris*, isolated from a decayed leaf of *Chusquea urelytra* (*Poaceae*) in the same Brazilian region. In the phylogenetic analyses (Figs 3, 9, Suppl. materials 1–3, 7, 8), these species are indistinguishable, as they share overlapping morphological characteristics (Table 3) and exhibit only a few differences in their DNA sequences: the type sequence of *C. brigadeirense* (COAD 2257) differs from the type sequence of *C. puris* (COAD 2487) by 3 bp in the *ACT* sequence, 5 bp in the *TEF1-α* sequence, and 2 bp in the *TUB* sequence; the ITS and *RPB2* sequences showed no base pair difference. Therefore, *C. brigadeirense* is here reduced to a synonym of *C. puris*. This study expands the known geographical distribution and ecological niche of *C. puris* to a Cerrado cave (found at a sampling point outside the cave)

#### Cladosporium sphaerospermum

Taxon classificationAnimaliaCladosporialesCladosporiaceae

Penz., (1882)

E1FB8ADA-B77A-56D8-904D-4D45DBA089E3

##### Synonym.

*Cladosporium marinisedimentum* W.Jun Lee & Y.W. Lim, MycoKeys 98: 98 (2023), syn. nov.

##### Typification.

ITALY • Padova, on faded leaves and stems of *Citrus* sp. (*Rutaceae*), Feb. 1882, O. Penzig (not preserved). NETHERLANDS • from the nail of man, 1949, coll. and isol. R.W. Zappey (neotype CBS H-19738, ex-neotype culture CBS 193.54 = ATCC 11289 = IMI 049637).

##### Description and illustration.

See Bensch et al. (2012).

##### Material examined.

BRAZIL • Goiás state, Mambaí municipality, Lapa do Penhasco cave, isol. from soil, May 2023, coll. J.F.S.A. Prazeres, E.O. Fonseca & J.D.P. Bezerra, isol. J.F.S.A. Prazeres (isolate FCCUFG 111), Gruna da Tarimba cave, isol. from soil by E.O. Fonseca (isolate FCCUFG 62); • Goiás state, Vila Propício municipality, from Lapa do Boqueirão cave, isol. from air, May 2022, coll. P.H. Félix-Oliveira & J.D.P. Bezerra, isol. P.H. Félix-Oliveira (isolates JB798, FCCUFG 204, FCCUFG 132, FCCUFG 140, and JB810).

##### Notes.

*Cladosporium sphaerospermum* (*C. sphaerospermum*SC) was described by Penzig (1882) from decaying leaves and branches of *Citrus (Rutaceae)* in Italy. *Cladosporium marinisedimentum* was proposed by Lee et al. (2023) for a fungus isolated from deep-sea sediments and sea sands in the Republic of Korea. These species are indistinguishable based on the phylogenetic analyses (Figs 4, 9, Suppl. materials 4–7) and share overlapping morphological characteristics (Table 3), as well as exhibiting few differences in their DNA sequences: the type sequence of *C. marinisedimentum* (SFC20230103-M28) differs from the type sequence of *C. sphaerospermum* (CBS 193.54) by 4 bp in the *ACT* sequence and 18 bp in the *TEF1-α* sequence; the ITS sequence has no base pair difference. The PHI analysis showed significant recombination, indicating intraspecific variation (Fig. 8A). Thus, *C. marinisedimentum* is here synonymised with *C. sphaerospermum*. These isolates were found at sampling points both inside and outside the caves.

#### Cladosporium subuliforme

Taxon classificationAnimaliaCladosporialesCladosporiaceae

Bensch, Crous & U. Braun, (2010)

F553059F-8DD5-5020-A5A4-106B0ADA51E9

##### Typification.

THAILAND • Chiang Mai, Sansai, Mai Jo, palm nursery, isol. from *Chamaedorea metallica* (*Arecaceae*), Dec. 2006, coll. I. Hidayat & J. Meeboon FIH 401, isol. P.W. Crous (holotype CBS H-20448, ex-type culture CBS 126500 = CPC 13735).

##### Description and illustration.

Bensch et al. (2010).

##### Material examined.

BRAZIL • Goiás state, Mambaí municipality, Lapa do Córrego das Dores cave, isol. from air, May 2023, coll. J.F.S.A. Prazeres, E.O. Fonseca & J.D.P. Bezerra, isol. E.O. Fonseca (isolates FCCUFG 110, JB1530, JB1569, and JB1540), and Gruna da Tarimba cave, isol. from air by E.O. Fonseca (isolates FCCUFG 127 and JB1497); • Goiás state, Vila Propício municipality, from Lapa do Boqueirão cave, isol. from air, May 2022, coll. P.H. Félix-Oliveira & J.D.P. Bezerra, isol. P.H. Félix-Oliveira (isolates FCCUFG 119, FCCUFG 121, JB794, JB813, and JB802).

##### Notes.

*Cladosporium subuliforme* (*C. cladosporioides*SC) was introduced by Bensch et al. (2010) for a fungus from *Chamaedorea metallica* (*Arecaceae*) in Thailand. The 10 isolates obtained from air formed a well-supported clade with the ex-type strain of *C. subuliforme*. The present study expands knowledge of this species, constituting the first report from a Brazilian cave (found at sampling points both inside and outside the caves).

#### Cladosporium velox

Taxon classificationAnimaliaCladosporialesCladosporiaceae

Zalar, de Hoog & Gunde-Cim., (2007)

CEAB2BF6-F28D-55C0-A87E-C9D4317F7CB8

##### Typification.

INDIA • Charidij, isol. from *Bambusa* sp., W. Gams (holotype CBS H-19735, ex-type culture CBS 119417).

##### Description and illustration.

Zalar et al. (2007).

##### Materials examined.

BRAZIL • Goiás state, Mambaí municipality, Lapa do Penhasco cave, isol. from soil, May 2023, coll. J.F.S.A. Prazeres, E.O. Fonseca & J.D.P. Bezerra, isol. J.F.S.A. Prazeres (isolate JB1953); • Goiás state, Vila Propício municipality, from Lapa do Boqueirão cave, isol. from air (isolates JB790 and FCCUFG 128) and soil (isolates JB797 and JB814), May 2022, coll. P.H. Félix-Oliveira & J.D.P. Bezerra, isol. P.H. Félix-Oliveira, Garganta (Samambaia) cave, isol. from air, Sep. 2022, coll. P.H. Félix-Oliveira & J.D.P. Bezerra, isol. P.H. Félix-Oliveira (isolate FCCUFG 63).

##### Notes.

*Cladosporium velox* (*C. sphaerospermum*SC), described by Zalar et al. (2007), was initially isolated from bamboo (*Bambusa* sp.) in India and has also been recorded from hypersaline water in Slovenia. Additionally, it has been identified as an agent of cotton disease in China (Li et al. 2023). The six isolates, obtained from air and soil of three caves, formed a well-supported clade with the ex-type strain of *C. velox* (Figs 4, 9). This study expands knowledge of the ecology of this species, constituting the first report from a Brazilian cave (found at sampling points both inside and outside the caves).

#### Cladosporium wenganense

Taxon classificationAnimaliaCladosporialesCladosporiaceae

Y.Q. Yang & Yong Wang bis [as ‘ wenganensis’], (2023)

3763E2EC-F603-581A-8C93-975EE0ADB611

##### Typification.

CHINA • Guizhou Province, Wengan County, isol. from leaves of *Prunus persica* (*Rosaceae)*, Jun. 2021, Y.Q. Yang (holotype HGUP 21220, ex-type culture GUCC 21220.1).

##### Description and illustration.

Yang et al. (2023).

##### Material examined.

BRAZIL • Goiás state, Mambaí municipality, Lapa do Córrego das Dores cave, isol. from air, May 2023, coll. J.F.S.A. Prazeres, E.O. Fonseca & J.D.P. Bezerra, isol. E.O. Fonseca (isolates JB1541); • Goiás state, Vila Propício municipality, from Lapa do Boqueirão cave, isol. from air, May 2022, coll. P.H. Félix-Oliveira & J.D.P. Bezerra, isol. P.H. Félix-Oliveira (isolates FCCUFG 117).

##### Notes.

*Cladosporium wenganense* (*C. cladosporioides*SC) was introduced by Yang et al. (2023) from the leaves of *Prunus persica* (*Rosaceae*) in China. One isolate obtained from air formed a well-supported lineage with the ex-type strain of *C. wenganense* (Fig. 3). The present study expands understanding of its ecology, constituting the first report from a Brazilian cave (found at a sampling point inside the cave).

#### Cladosporium xanthochromaticum

Taxon classificationAnimaliaCladosporialesCladosporiaceae

Sand.-Den., Gené & Cano, (2016)

88C17EDD-3A05-5DD1-9A90-841C82CF3F67

##### Typification.

USA • Texas, isol. from human bronchoalveolar lavage fluid, Sep. 2010, D.A. Sutton (holotype CBS H-22388, ex-type culture CBS 140691 = UTHSC DI-13-211 = FMR 13324).

##### Description and illustration.

Sandoval-Denis et al. (2016).

##### Material examined.

BRAZIL • Goiás state, Mambaí municipality, Gruna da Tarimba cave, isol. from air, May 2023, coll. J.F.S.A. Prazeres, E.O. Fonseca & J.D.P. Bezerra, isol. E.O. Fonseca (isolate JB1499); • Goiás state, Vila Propício municipality, from Lapa do Boqueirão cave, isol. from air, May 2022, coll. P.H. Félix-Oliveira & J.D.P. Bezerra, isol. P.H. Félix-Oliveira (isolate JB799), Garganta (Samambaia) cave, isol. from air, Sep. 2022, coll. P.H. Félix-Oliveira & J.D.P. Bezerra, isol. P.H. Félix-Oliveira (isolate JB1456).

##### Notes.

*Cladosporium xanthochromaticum* (*C. cladosporioides*SC), described by Sandoval-Denis et al. (2016), was initially isolated from human bronchoalveolar lavage fluid in the USA and has also been recorded as an endophytic fungus from *Camellia sinensis* (*Theaceae*) in China (Lv et al. 2023). In the present study, three isolates obtained from air of three Cerrado caves formed a well-supported clade with the ex-type strain of *C. xanthochromaticum* (Fig. 3). Previously recorded in a cave from the Caatinga biome, the present study constitutes the first report of this species from a cave in the Cerrado biome (found at a sampling point inside the caves), thus expanding its known distribution within Brazilian subterranean ecosystems.

### Checklist of *Cladosporium* spp. in Brazilian caves

The list of *Cladosporium* species recorded in Brazilian caves reveals remarkable diversity, highlighting the wide distribution and adaptability of these species to different conditions. In Brazil, *C. cladosporioides* has been the most frequently reported species from caves (Taylor et al. 2013, 2014; Alves et al. 2022; Prazeres et al. 2025). However, in this study, no isolates were identified as *C. cladosporioides*; instead, other species within the same species complex were abundantly found (Suppl. material 9: table SS2). Among the Brazilian biomes, the Cerrado has the most significant number of studies on cave fungi, with the genus *Cladosporium* being the most frequently recorded (Prazeres et al. 2025). This can be attributed to the greater amount of research conducted in this biome, reflecting its relevance as a global biodiversity hotspot and the high diversity of underground environments in the region (Prazeres et al. 2025). Additionally, *C. herbarum*, *C. oxysporum*, *C. subuliforme*, *C. tenuissimum*, *C. xanthochromaticum*, *C. austrohemisphaericum*, *C. parahalotolerans*, *C. puris*, *C. sphaerospermum*, *C. diamantinense*, and *C. speluncae* were documented in caves of the Caatinga, Cerrado, and Atlantic Forest biomes, and most studies have isolated *Cladosporium* species from cave air; however, they have also been found in soil, bat ectoparasites, and bat guano (Alves et al. 2022; Carvalho et al. 2022; Pereira et al. 2022; Dutra et al. 2023; Lima et al. 2024; Prazeres et al. 2025).

This study expanded this list by including 23 species distributed across two *Cladosporium* SCs (Table 2, Suppl. material 9: table SS2). The *C. cladosporioides*SC included 19 species: *C. angulosum*, *C. aulonemiae*, *C. bambusicola*, *C. chlamydosporiformans*, *C. flabelliforme*, *C. carsi* sp. nov., *C. lacerdae* sp. nov., *C. macadamiae*, *C. mambaiense* sp. nov., *C. nogueirae* sp. nov., *C. perangustum*, *C. pernambucoense*, *C. pruni-salicinae*, *C. pseudocladosporioides*, *C. puris*, *C. propiciense* sp. nov., *C. subuliforme*, *C. wenganense*, and *C. xanthochromaticum*. The *C. sphaerospermum*SC included *C. aciculare*, *C. mesquitapaivae* sp. nov., *C. sphaerospermum*, and *C. velox*. Among the species recorded in this study, those documented for the first time in Brazilian caves are highlighted: *C. angulosum*, *C. aulonemiae*, *C. chlamydosporiformans*, *C. flabelliforme*, *C. macadamiae*, *C. perangustum*, *C. pruni-salicinae*, *C. pseudocladosporioides*, *C. wenganense*, *C. aciculare*, *C. velox*, and the new species introduced here (Tables 2, 3).

## Discussion

This study substantially advances understanding of *Cladosporium* richness in Brazilian caves by analysing 94 isolates from soil and air samples of six caves in the Cerrado biome. Among these, 23 species were identified as belonging to the *C. cladosporioides*SC and *C. sphaerospermum*SC, and six new species (*C. carsi*, *C. lacerdae*, *C. mambaiense*, *C. mesquitapaivae*, *C. nogueirae*, and *C. propiciense*) were described through an integrative approach combining morphological and multi-locus DNA analyses. This methodological rigour underscores the importance of combining molecular and morphological data for the robust taxonomy of *Cladosporium* from understudied environments. In addition, phylogenetic and morphological analyses led to the synonymy of *C. ribis* and *C. speluncae* under *C. bambusicola*, *C. brigadeirense* under *C. puris*, and *C. marinisedimentum* under *C. sphaerospermum*. These findings provide critical insights into the evolutionary relationships within the genus and demonstrate the taxonomic complexity of fungi inhabiting caves. Additionally, this study also showed that the inclusion of new isolates expands the geographical distribution of known *Cladosporium* species. The analysis of a larger collection also favours multi-locus analyses and makes the re-evaluation of species boundaries within *Cladosporium* possible (Wirsel et al. 2002).

The adoption of combined methods, including morphology, physiology, and molecular analyses (multi-locus phylogenetic analysis and PHI test), has contributed substantially to increasing the accuracy and reliability of *Cladosporium* species identification. Species delimitation in this genus based solely on morphology has become challenging due to the overlap of macro- and micromorphological characteristics (Bensch et al. 2010, 2012; Marin-Felix et al. 2017). In this study, the *ACT* and *TEF1-α* markers were initially the most informative, being particularly effective in identifying *Cladosporium* species. However, ITS sequences were challenging for resolving many species in the *C. cladosporioides*SC, as they demonstrated no differences between phylogenetically related species. *ACT* and *TEF1-α* are currently the accepted genes for analysing *Cladosporium* species and the stability of lineages/clades (Braun et al. 2003; Bensch et al. 2010). By contrast, ITS was more informative among species of the *C. sphaerospermum*SC and had satisfactory resolution when compared to *ACT* and *TEF1-α*. In the *C. sphaerospermum*SC, the ITS phylogeny was capable of resolving 100% of the species. Species in the *C. sphaerospermum*SC are characterised by significant genetic diversity, which is evident in the variability of ITS sequences (Zalar et al. 2007; Bensch et al. 2012).

To assist the phylogenetic analyses, the PHI test was used to refine the analysis of these results and to complement the GCPSR criteria (Taylor et al. 2000). Thus, the synonyms and novelties proposed here were tested for possible genetic recombination to demonstrate their evolutionary independence and show that they constituted independent taxonomic lineages. Significantly, the results of the PHI test helped to confirm the phylogenetic relationships predicted by the multi-locus analyses (*ACT*, ITS, and *TEF1-α*), providing new evidence for the analysis of species boundaries in *Cladosporium*. To better understand the new *Cladosporium* species introduced here, mainly in the *C. cladosporioides*SC, the addition of *RPB2* and *TUB* sequences proved to be important for investigating species boundaries, particularly *RPB2*, which showed greater genetic distance and provided resolution for the species analysed. Furthermore, a combined matrix of five genes (*ACT*, ITS, *RPB2*, *TEF1-α*, and *TUB*) confirmed the phylogenetic decisions and assisted further delimitation of closely related species. For example, Schubert et al. (2007) analysed the *C. herbarum*SC based on five markers (*ACT*, calmodulin, ITS, *TEF1-α*, and histone H3) and demonstrated better distinction of closely phylogenetically related species. Later, Bensch et al. (2010) utilised the currently recommended markers for identifying *Cladosporium* species (*ACT*, ITS, and *TEF1-α*) to enhance the resolution of species distinction within the *C. cladosporioides* complex. These authors suggested the need for additional markers to resolve some clades or species within this complex (Bensch et al. 2010, 2012). Later, other markers were adopted for genera related to *Cladosporium* (e.g. Bezerra et al. 2017). Recently, based on comparative genome analysis, Zheng et al. (2025) provided important information for elucidating species boundaries among *Cladosporium* species complexes. The use of *ACT*, *RPB2*, *TEF1-α*, and *TUB* as the main phylogenetic barcodes to recognise *Cladosporium* species is proposed here, particularly within the *C. cladosporioides*SC.

Before this study, only 14 species of *Cladosporium* had been reported in Brazilian caves (*C. anthropophilum*, *C. austrohemisphaericum*, *C. bambusicola*, *C. cavernicola*, *C. cladosporioides*, *C. diamantinense*, *C. halotolerans*, *C. oxysporum*, *C. parahalotolerans*, *C. pernambucoense*, *C. puris*, *C. sphaerospermum*, *C. subuliforme*, and *C. xanthochromaticum*), underscoring the rarity and significance of research focused exclusively on this genus in cave ecosystems (Cunha et al. 2020; Carvalho et al. 2022; Pereira et al. 2022; Alves et al. 2022; Dutra et al. 2023). Among these studies, the majority were based exclusively on morphological identification. This approach, although useful, often results in inaccurate and limited identifications, typically restricted to the genus level, without reaching species-level definition (Taylor et al. 2013, 2014).

Interestingly, Group 6 (“Brazilian clade”) treated here (Figs 3, 9) is a unique clade within the *C. cladosporioides*SC, encompassing species still reported only from Brazil (*C. aulonemiae*, *C. nogueirae* sp. nov., *C. propiciense* sp. nov., and *C. rugulovarians*). Bensch et al. (2015) introduced *C. rugulovarians* for a fungus isolated from leaf sheaths of unidentified *Poaceae* collected in Chapada dos Guimarães, a protected area of the Brazilian savannah (Cerrado) in the state of Mato Grosso. The authors described this species as “quite unique and only comparable with *C. varians*” (Bensch et al. 2015). Later, Costa et al. (2022) described *C. aulonemiae* for isolates obtained from the decayed leaves of *A. amplissima* (*Poaceae*) in the Parque Estadual Serra do Brigadeiro, a protected area of the Atlantic Forest in the state of Minas Gerais. Phylogenetically, Costa et al. (2022) also showed *C. aulonemiae* as a unique lineage within the *C. cladosporioides*SC, related only to *C. rugulovarians*. Both sites of the first isolation of *C. aulonemiae* and *C. rugulovarians* are approximately 1,500 km apart and protect different biomes as conservation units in the country. The introduction of *C. nogueirae* sp. nov. and *C. propiciense* sp. nov., related to these species, is particularly interesting, as they were isolated from the air of two caves in the Cerrado biome, State of Goiás. These caves are approximately 7 km apart, and the municipality where they are located is approximately 1,000 km from the areas where *C. aulonemiae* and *C. rugulovarians* were first collected. Additionally, *C. aulonemiae* is reported here for the first time from a cave, and no reports of it or *C. rugulovarians* have been found since their description. These species remain unique lineages within the *C. cladosporioides*SC, and further study, including new isolates, will help to uncover the substrate/ecosystem specificity, geographical distribution, and intraspecific variation of species in this clade.

These findings substantially expanded the known diversity of *Cladosporium* species in cave ecosystems by describing six new species and adding several new records for Brazilian caves, highlighting the Cerrado as an important reservoir of fungal diversity. Among the species found here, *C. bambusicola* was the most abundant, representing 29.17% of the isolates, followed by *C. velox* (15.51%). The detection of *C. bambusicola* in cave environments marks its first record in this ecosystem. Initially described from plant material in Brazil (Costa et al. 2022), *C. bambusicola* was also recently reported as a potential mycoparasite of *Hemileia vastatrix* in Brazil (Pereira et al. 2024). Its presence in caves may highlight the remarkable adaptability of this species to different environments, suggesting ecological plasticity and novel roles in subterranean ecosystems. Except for the new species introduced here, most of the *Cladosporium* species found in caves have already been reported as plant pathogens (Bhartiya et al. 2022; Yang et al. 2023), endophytes (Yang et al. 2023), or associated with soils and other substrates (Nicoletti et al. 2024; Li et al. 2024; Farwell et al. 2024; Liu et al. 2024), reinforcing the hypothesis of the external origin of fungi in caves (Zhang et al. 2018). The presence of these organisms in cave environments highlights the influence of external sources on the underground ecosystem, emphasising the complexity of ecological interactions in this unique habitat (Zhang et al. 2018).

The genus *Cladosporium* is an increasing component of global cave mycodiversity. In a global analysis based on 14 studies of fungi in caves, Biagioli et al. (2023) positioned *Cladosporium* (11.89%) among the most abundant genera of *Ascomycota*. The same study demonstrated that climatic conditions and external environments may influence subterranean microbial patterns. This global trend is illustrated by regional studies, such as those of Poli et al. (2024) in Italian caves, which observed that sediments in a tourist cave hosted a diverse fungal community compared to the external environment, possibly due to visitors transporting propagules or organic material. In Slovakian caves, Ogórek et al. (2024) reported *C. ramotenellum* among the fungal community. In Brazil, the dominance of *Cladosporium* spp. in caves is particularly evident, highlighting the presence of these species on multiple substrates within caves (Prazeres et al. 2025). This notable species diversity was exemplified by Pereira et al. (2022), who found eight *Cladosporium* spp. while studying a cave in the Caatinga biome. The ubiquity of *Cladosporium* in Brazilian biomes may also support the theory proposed by Finlay (2002), which suggests that the success of this genus is due to the long-distance dispersal of its pigmented and desiccation-resistant mitotic spores (conidia).

These findings highlight the ecological significance and adaptive potential of *Cladosporium* species in subterranean environments, confirming the value of tropical caves for targeted studies in unique biomes, such as the Cerrado in Brazil. Additionally, the importance of utilising modern phylogenetic analysis based on multi-locus markers in identifying both known and new species is emphasised. The addition of tropical isolates to phylogenetic studies has revealed the distribution of known *Cladosporium* species and helped clarify their relationships with other lineages, thereby enhancing understanding of species delimitation and intraspecific variation within species and species complexes. The high diversity and novel occurrences of *Cladosporium* species in Brazilian caves provide valuable insights into fungal ecology and evolution in these habitats. It is also worth noting that this high level of diversity is observed despite the inherent limitations of the passive sampling method, which is susceptible to air currents and gravitational settling of particles, factors that may affect the estimation of the fungal community in cave air. Knowledge of the mycobiome of caves may contribute to the protection of their unique biodiversity and offer insights into taxonomy, ecological services, pathogenicity, geographical distribution, potential biotechnological applications, and fungal survival mechanisms in caves.

## Supplementary Material

XML Treatment for Cladosporium aciculare

XML Treatment for Cladosporium angulosum

XML Treatment for Cladosporium aulonemiae

XML Treatment for Cladosporium bambusicola

XML Treatment for Cladosporium carsi

XML Treatment for Cladosporium chlamydosporiformans

XML Treatment for Cladosporium flabelliforme

XML Treatment for Cladosporium lacerdae

XML Treatment for Cladosporium macadamiae

XML Treatment for Cladosporium mambaiense

XML Treatment for Cladosporium mesquitapaivae

XML Treatment for Cladosporium nogueirae

XML Treatment for Cladosporium perangustum

XML Treatment for Cladosporium pernambucoense

XML Treatment for Cladosporium propiciense

XML Treatment for Cladosporium pruni-salicinae

XML Treatment for Cladosporium pseudocladosporioides

XML Treatment for Cladosporium puris

XML Treatment for Cladosporium sphaerospermum

XML Treatment for Cladosporium subuliforme

XML Treatment for Cladosporium velox

XML Treatment for Cladosporium wenganense

XML Treatment for Cladosporium xanthochromaticum

## Additional information

### Artificial Intelligence (AI) use

The authors accept full responsibility for the content of the manuscript, including the disclosure of any use of AI.

Regarding the use of AI in the preparation of this manuscript, the authors declare the following: Grammarly and DeepL.

**Used for**: Language, style and writing.

### Adherence to national and international regulations

The sampling expedition into Brazilian caves was authorised by the Ministério do Meio Ambiente (MMA)/Instituto Chico Mendes de Conservação da Biodiversidade (ICMBio) (permit number: 82254-5), and the study was registered in the Sistema Nacional de Gestão do Patrimônio Genético (SisGen)/MMA/Conselho de Gestão do Patrimônio Genético (CGen) (registration number: ACD07BF).

### Author ORCIDs

Pedro H. Félix-Oliveira https://orcid.org/0009-0004-6816-6282

José F. S. A. Prazeres https://orcid.org/0000-0003-1188-035X

Layanne O. Ferro https://orcid.org/0000-0003-3757-0085

Ana C. A. Antunes https://orcid.org/0009-0003-0501-2959

Caroline F. Lima https://orcid.org/0009-0003-7648-2843

Dominnyke S. S. Neves https://orcid.org/0009-0001-1277-3903

Emily O. Fonseca https://orcid.org/0000-0002-1751-0424

Lorena S. Miranda https://orcid.org/0000-0002-0600-784X

Renata S. Momoli https://orcid.org/0000-0001-7309-4974

Cristina M. Souza-Motta https://orcid.org/0000-0002-0964-8271

Konstanze Bensch https://orcid.org/0009-0006-3404-9955

Pedro W. Crous https://orcid.org/0000-0001-9085-8825

Jadson D. P. Bezerra https://orcid.org/0000-0002-7782-8602

### Data availability

Fungal isolates are deposited in public culture collections in Brazil, DNA sequences are available in GenBank, and alignments were deposited in Figshare.

## Supplementary materials

Supplementary material 1 Supplementary image 1 Data type pdf Explanation note Maximum-likelihood IQTree tree of *C. cladosporioides*SC based on an individual dataset of *ACT* sequences. The species obtained in this study are highlighted in bold. Ex-type strains = T. IQ-TREE-BS values ≥ 70% and RAxML-BS ≥ 70% are included next to the nodes. The tree was rooted with *Cladosporium herbarum* (CBS 121621 and CBS 300.49). https://binary.pensoft.net/file/1667893This dataset is made available under the Open Database License (http://opendatacommons.org/licenses/odbl/1.0/). The Open Database License (ODbL) is a license agreement intended to allow users to freely share, modify, and use this Dataset while maintaining this same freedom for others, provided that the original source and author(s) are credited. Pedro H. Félix-Oliveira, José F. S. A. Prazeres, Layanne O. Ferro, Ana C. A. Antunes, Caroline F. Lima, Dominnyke S. S. Neves, Emily O. Fonseca, Lorena S. Miranda, Renata S. Momoli, Cristina M. Souza-Motta, Konstanze Bensch, Pedro W. Crous, Jadson D. P. Bezerra

Supplementary material 2 Supplementary image 2 Data type pdf Explanation note Maximum-likelihood IQTree tree of *C. cladosporioides*SC based on an individual dataset of ITS sequences. The species obtained in this study are highlighted in bold. Ex-type strains = T. IQ-TREE-BS values ≥ 70% and RAxML-BS ≥ 70% are included next to the nodes. The tree was rooted with *Cladosporium herbarum* (CBS 121621 and CBS 300.49) https://binary.pensoft.net/file/1667894This dataset is made available under the Open Database License (http://opendatacommons.org/licenses/odbl/1.0/). The Open Database License (ODbL) is a license agreement intended to allow users to freely share, modify, and use this Dataset while maintaining this same freedom for others, provided that the original source and author(s) are credited. Pedro H. Félix-Oliveira, José F. S. A. Prazeres, Layanne O. Ferro, Ana C. A. Antunes, Caroline F. Lima, Dominnyke S. S. Neves, Emily O. Fonseca, Lorena S. Miranda, Renata S. Momoli, Cristina M. Souza-Motta, Konstanze Bensch, Pedro W. Crous, Jadson D. P. Bezerra

Supplementary material 3 Supplementary image 3 Data type pdf Explanation note Maximum-likelihood IQTree tree of *C. cladosporioides*SC based on an individual dataset of *TEF1-α* sequences. The species obtained in this study are highlighted in bold. Ex-type strains = T. IQ-TREE-BS values ≥ 70% and RAxML-BS ≥ 70% are included next to the nodes. The tree was rooted with *Cladosporium herbarum* (CBS 121621 and CBS 300.49) https://binary.pensoft.net/file/1667895This dataset is made available under the Open Database License (http://opendatacommons.org/licenses/odbl/1.0/). The Open Database License (ODbL) is a license agreement intended to allow users to freely share, modify, and use this Dataset while maintaining this same freedom for others, provided that the original source and author(s) are credited. Pedro H. Félix-Oliveira, José F. S. A. Prazeres, Layanne O. Ferro, Ana C. A. Antunes, Caroline F. Lima, Dominnyke S. S. Neves, Emily O. Fonseca, Lorena S. Miranda, Renata S. Momoli, Cristina M. Souza-Motta, Konstanze Bensch, Pedro W. Crous, Jadson D. P. Bezerra

Supplementary material 4 Supplementary image 4 Data type pdf Explanation note Maximum-likelihood IQTree tree of *C. sphaerospermum*SC based on an individual dataset of *ACT* sequences. The species obtained in this study are highlighted in bold. Ex-type strains = T. IQ-TREE-BS values ≥ 70% and RAxML-BS ≥ 70% are included next to the nodes. The tree was rooted with *Cladosporium herbarum* (CBS 121621 and CBS 300.49) https://binary.pensoft.net/file/1667896This dataset is made available under the Open Database License (http://opendatacommons.org/licenses/odbl/1.0/). The Open Database License (ODbL) is a license agreement intended to allow users to freely share, modify, and use this Dataset while maintaining this same freedom for others, provided that the original source and author(s) are credited. Pedro H. Félix-Oliveira, José F. S. A. Prazeres, Layanne O. Ferro, Ana C. A. Antunes, Caroline F. Lima, Dominnyke S. S. Neves, Emily O. Fonseca, Lorena S. Miranda, Renata S. Momoli, Cristina M. Souza-Motta, Konstanze Bensch, Pedro W. Crous, Jadson D. P. Bezerra

Supplementary material 5 Supplementary image 5 Data type pdf Explanation note Maximum-likelihood IQTree tree of *C. sphaerospermum*SC based on an individual dataset of ITS sequences. The species obtained in this study are highlighted in bold. Ex-type strains = T. IQ-TREE-BS values ≥ 70%, and RAxML-BS ≥ 70% are included next to the nodes. The tree was rooted with *Cladosporium herbarum* (CBS 121621 and CBS 300.49) https://binary.pensoft.net/file/1667897This dataset is made available under the Open Database License (http://opendatacommons.org/licenses/odbl/1.0/). The Open Database License (ODbL) is a license agreement intended to allow users to freely share, modify, and use this Dataset while maintaining this same freedom for others, provided that the original source and author(s) are credited. Pedro H. Félix-Oliveira, José F. S. A. Prazeres, Layanne O. Ferro, Ana C. A. Antunes, Caroline F. Lima, Dominnyke S. S. Neves, Emily O. Fonseca, Lorena S. Miranda, Renata S. Momoli, Cristina M. Souza-Motta, Konstanze Bensch, Pedro W. Crous, Jadson D. P. Bezerra

Supplementary material 6 Supplementary image 6 Data type pdf Explanation note Maximum-likelihood IQTree tree of *C. sphaerospermum*SC based on an individual dataset of *TEF1-α* sequences. The species obtained in this study are highlighted in bold. Ex-type strains = T. IQ-TREE-BS values ≥ 70% and RAxML-BS ≥ 70% are included next to the nodes. The tree was rooted with *Cladosporium herbarum* (CBS 121621 and CBS 300.49) https://binary.pensoft.net/file/1667898This dataset is made available under the Open Database License (http://opendatacommons.org/licenses/odbl/1.0/). The Open Database License (ODbL) is a license agreement intended to allow users to freely share, modify, and use this Dataset while maintaining this same freedom for others, provided that the original source and author(s) are credited. Pedro H. Félix-Oliveira, José F. S. A. Prazeres, Layanne O. Ferro, Ana C. A. Antunes, Caroline F. Lima, Dominnyke S. S. Neves, Emily O. Fonseca, Lorena S. Miranda, Renata S. Momoli, Cristina M. Souza-Motta, Konstanze Bensch, Pedro W. Crous, Jadson D. P. Bezerra

Supplementary material 7 Supplementary image 7 Data type pdf Explanation note Maximum-likelihood IQTree tree of *C. cladosporioides*SC and *C. sphaerospermum*SC based on an individual dataset of *RPB2* sequences. The species obtained in this study are highlighted in bold. Ex-type strains = T. IQ-TREE-BS values ≥ 70% and RAxML-BS ≥ 70% are included next to the nodes. The tree was rooted with *Cercospora beticola* (CBS 116456) https://binary.pensoft.net/file/1667899This dataset is made available under the Open Database License (http://opendatacommons.org/licenses/odbl/1.0/). The Open Database License (ODbL) is a license agreement intended to allow users to freely share, modify, and use this Dataset while maintaining this same freedom for others, provided that the original source and author(s) are credited. Pedro H. Félix-Oliveira, José F. S. A. Prazeres, Layanne O. Ferro, Ana C. A. Antunes, Caroline F. Lima, Dominnyke S. S. Neves, Emily O. Fonseca, Lorena S. Miranda, Renata S. Momoli, Cristina M. Souza-Motta, Konstanze Bensch, Pedro W. Crous, Jadson D. P. Bezerra

Supplementary material 8 Supplementary image 8 Data type pdf Explanation note Maximum-likelihood IQTree tree of *C. cladosporioides*SC and *C. sphaerospermum*SC based on an individual dataset of *TUB* sequences. The species obtained in this study are highlighted in bold. Ex-type strains = T. IQ-TREE-BS values ≥ 70% and RAxML-BS ≥ 70% are included next to the nodes. The tree was rooted with *Cercospora beticola* (CBS 116456). https://binary.pensoft.net/file/1667900This dataset is made available under the Open Database License (http://opendatacommons.org/licenses/odbl/1.0/). The Open Database License (ODbL) is a license agreement intended to allow users to freely share, modify, and use this Dataset while maintaining this same freedom for others, provided that the original source and author(s) are credited. Pedro H. Félix-Oliveira, José F. S. A. Prazeres, Layanne O. Ferro, Ana C. A. Antunes, Caroline F. Lima, Dominnyke S. S. Neves, Emily O. Fonseca, Lorena S. Miranda, Renata S. Momoli, Cristina M. Souza-Motta, Konstanze Bensch, Pedro W. Crous, Jadson D. P. Bezerra

Supplementary material 9 Supplementary tables Data type pdf Explanation note **table SS1**. Feature summaries of the studied caves, including geographic coordinates, number of sampling points, count of fungal isolates by substrate (air and soil), temperature, and humidity. **table SS2**. Checklist of *Cladosporium* species reported in caves of different Brazilian biomes, with details on the location, substrate, and reference for each record. https://binary.pensoft.net/file/1667901This dataset is made available under the Open Database License (http://opendatacommons.org/licenses/odbl/1.0/). The Open Database License (ODbL) is a license agreement intended to allow users to freely share, modify, and use this Dataset while maintaining this same freedom for others, provided that the original source and author(s) are credited. Pedro H. Félix-Oliveira, José F. S. A. Prazeres, Layanne O. Ferro, Ana C. A. Antunes, Caroline F. Lima, Dominnyke S. S. Neves, Emily O. Fonseca, Lorena S. Miranda, Renata S. Momoli, Cristina M. Souza-Motta, Konstanze Bensch, Pedro W. Crous, Jadson D. P. Bezerra
