# Supplementary material for: Cladosporium from caves of the Brazilian savannah (Cerrado) and the description of six new species
Source: IMA Fungus. 2026 Jun 3;17:e191673. doi: 10.3897/imafungus.17.191673 (PMC13254554; doi:10.3897/imafungus.17.191673)
Supplement: Supplementary material 1 — Supplementary image 1 [file imafungus-17-e191673-s001.pdf]

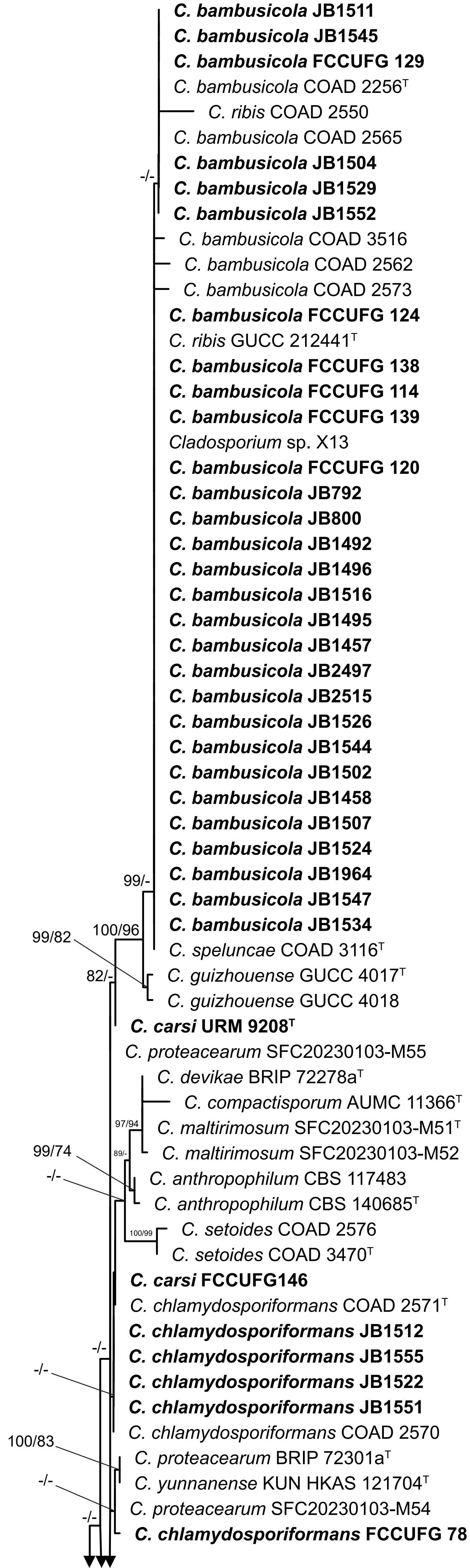

C. bambusicola JB1511
C. bambusicola JB1545
C. bambusicola FCCUFG 129
C. bambusicola COAD 2256T
C. ribis COAD 2550
C. bambusicola COAD 2565
C. bambusicola JB1504
C. bambusicola JB1529
C. bambusicola JB1552
-/-
C. bambusicola COAD 3516
C. bambusicola COAD 2562
C. bambusicola COAD 2573
C. bambusicola FCCUFG 124
C. ribis GUCC 212441T
C. bambusicola FCCUFG 138
C. bambusicola FCCUFG 114
C. bambusicola FCCUFG 139
Cladosporium sp. X13
C. bambusicola FCCUFG 120
C. bambusicola JB792
C. bambusicola JB800
C. bambusicola JB1492
C. bambusicola JB1496
C. bambusicola JB1516
C. bambusicola JB1495
C. bambusicola JB1457
C. bambusicola JB2497
C. bambusicola JB2515
C. bambusicola JB1526
C. bambusicola JB1544
C. bambusicola JB1502
C. bambusicola JB1458
C. bambusicola JB1507
C. bambusicola JB1524
C. bambusicola JB1964
99/-
C. bambusicola JB1547
C. bambusicola JB1534
100/96
99/82
C. speluncae COAD 3116T
82/-
C. guizhouense GUCC 4017T
C. guizhouense GUCC 4018
C. carsi URM 9208T
C. proteacearum SFC20230103-M55
C. devikae BRIP 72278aT
C. compactisporum AUMC 11366T
97/94
C. maltirimosum SFC20230103-M51T
89/-
99/74
C. maltirimosum SFC20230103-M52
-/-
C. anthropophilum CBS 117483
C. anthropophilum CBS 140685T
100/99
C. setoides COAD 2576
C. setoides COAD 3470T
C. carsi FCCUFG146
C. chlamydosporiformans COAD 2571T
-/-
C. chlamydosporiformans JB1512
C. chlamydosporiformans JB1555
-/-
C. chlamydosporiformans JB1522
C. chlamydosporiformans JB1551
C. chlamydosporiformans COAD 2570
100/83
C. proteacearum BRIP 72301aT
-/-
C. yunnanense KUN HKAS 121704T
C. proteacearum SFC20230103-M54
-/-
C. chlamydosporiformans FCCUFG 78

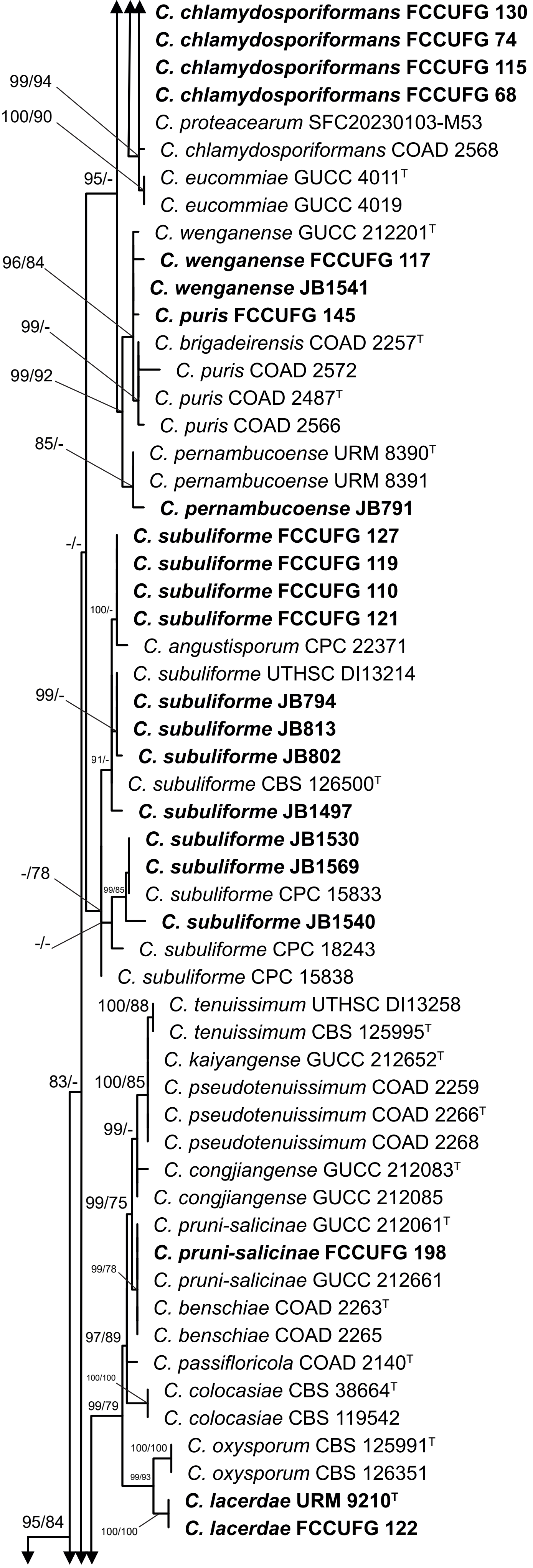
C. chlamydosporiformans FCCUFG 130
C. chlamydosporiformans FCCUFG 74
C. chlamydosporiformans FCCUFG 115
C. chlamydosporiformans FCCUFG 68
C. proteacearum SFC20230103-M53
C. chlamydosporiformans COAD 2568
C. eucommiae GUCC 4011T
C. eucommiae GUCC 4019
C. wenganense GUCC 212201T
C. wenganense FCCUFG 117
C. wenganense JB1541
C. puris FCCUFG 145
C. brigadeirensis COAD 2257T
C. puris COAD 2572
C. puris COAD 2487T
C. puris COAD 2566
C. pernambucoense URM 8390T
C. pernambucoense URM 8391
C. pernambucoense JB791
C. subuliforme FCCUFG 127
C. subuliforme FCCUFG 119
C. subuliforme FCCUFG 110
C. subuliforme FCCUFG 121
C. angustisporum CPC 22371
C. subuliforme UTHSC DI13214
C. subuliforme JB794
C. subuliforme JB813
C. subuliforme JB802
C. subuliforme CBS 126500T
C. subuliforme JB1497
C. subuliforme JB1530
C. subuliforme JB1569
C. subuliforme CPC 15833
C. subuliforme JB1540
C. subuliforme CPC 18243
C. subuliforme CPC 15838
C. tenuissimum UTHSC DI13258
C. tenuissimum CBS 125995T
C. kaiyangense GUCC 212652T
C. pseudotenuissimum COAD 2259
C. pseudotenuissimum COAD 2266T
C. pseudotenuissimum COAD 2268
C. congjiangense GUCC 212083T
C. congjiangense GUCC 212085
C. pruni-salicinae GUCC 212061T
C. pruni-salicinae FCCUFG 198
C. pruni-salicinae GUCC 212661
C. benschiae COAD 2263T
C. benschiae COAD 2265
C. passifloricola COAD 2140T
C. colocasiae CBS 38664T
C. colocasiae CBS 119542
C. oxysporum CBS 125991T
C. oxysporum CBS 126351
C. lacerdae URM 9210T
C. lacerdae FCCUFG 122
99/94
100/90
95/-
96/84
99/-
99/92
85/-
-/-
100/-
99/-
91/-
-/78
99/85
-/-
100/88
100/85
83/-
99/-
99/75
99/78
97/89
100/100
99/79
100/100
99/93
100/100
95/84

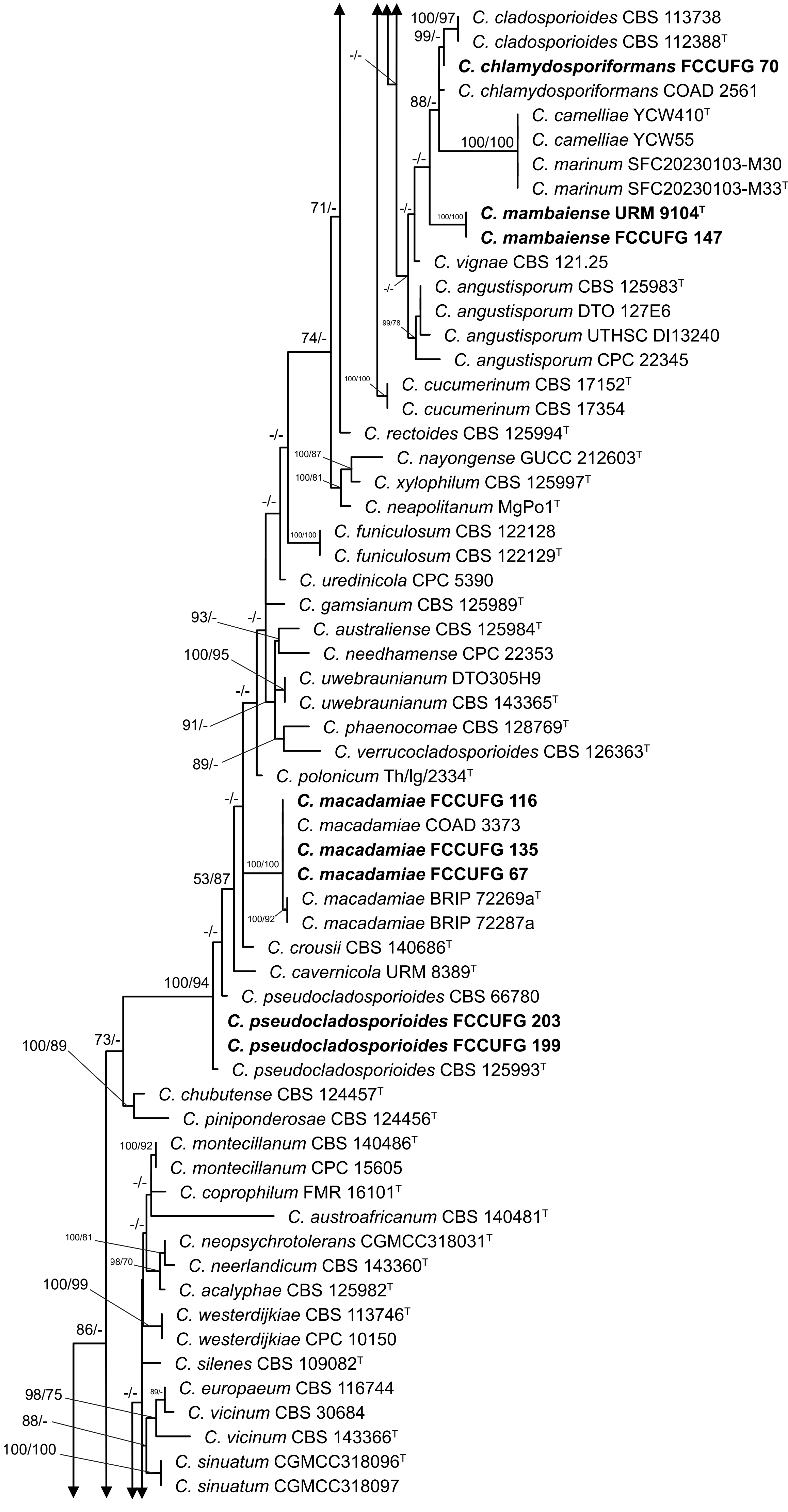
C. cladosporioides CBS 113738
C. cladosporioides CBS 112388^{T}
C. chlamydosporiformans FCCUFG 70
C. chlamydosporiformans COAD 2561
C. camelliae YCW410^{T}
C. camelliae YCW55
C. marinum SFC20230103-M30
C. marinum SFC20230103-M33^{T}
C. mambaiense URM 9104^{T}
C. mambaiense FCCUFG 147
C. vignae CBS 121.25
C. angustisporum CBS 125983^{T}
C. angustisporum DTO 127E6
C. angustisporum UTHSC DI13240
C. angustisporum CPC 22345
C. cucumerinum CBS 17152^{T}
C. cucumerinum CBS 17354
C. rectoides CBS 125994^{T}
C. nayongense GUCC 212603^{T}
C. xylophilum CBS 125997^{T}
C. neapolitanum MgPo1^{T}
C. funiculosum CBS 122128
C. funiculosum CBS 122129^{T}
C. uredinicola CPC 5390
C. gamsianum CBS 125989^{T}
C. australiense CBS 125984^{T}
C. needhamense CPC 22353
C. uwebraunianum DTO305H9
C. uwebraunianum CBS 143365^{T}
C. phaenocomae CBS 128769^{T}
C. verrucocladosporioides CBS 126363^{T}
C. polonicum Th/lg/2334^{T}
C. macadamiae FCCUFG 116
C. macadamiae COAD 3373
C. macadamiae FCCUFG 135
C. macadamiae FCCUFG 67
C. macadamiae BRIP 72269a^{T}
C. macadamiae BRIP 72287a
C. crousii CBS 140686^{T}
C. cavernicola URM 8389^{T}
C. pseudocladosporioides CBS 66780
C. pseudocladosporioides FCCUFG 203
C. pseudocladosporioides FCCUFG 199
C. pseudocladosporioides CBS 125993^{T}
C. chubutense CBS 124457^{T}
C. piniponderosae CBS 124456^{T}
C. montecillanum CBS 140486^{T}
C. montecillanum CPC 15605
C. coprophilum FMR 16101^{T}
C. austroafricanum CBS 140481^{T}
C. neopsychrotolerans CGMCC318031^{T}
C. neerlandicum CBS 143360^{T}
C. acalyphae CBS 125982^{T}
C. westerdijkiae CBS 113746^{T}
C. westerdijkiae CPC 10150
C. silenes CBS 109082^{T}
C. europaeum CBS 116744
C. vicinum CBS 30684
C. vicinum CBS 143366^{T}
C. sinuatum CGMCC318096^{T}
C. sinuatum CGMCC318097
100/97
99/-
-/-
88/-
100/100
-/-
-/-
71/-
74/-
99/78
-/-
100/87
100/81
100/100
93/-
100/95
91/-
89/-
53/87
100/92
100/94
73/-
100/89
86/-
100/99
98/75
88/-
100/100
98/70
100/81
89/-

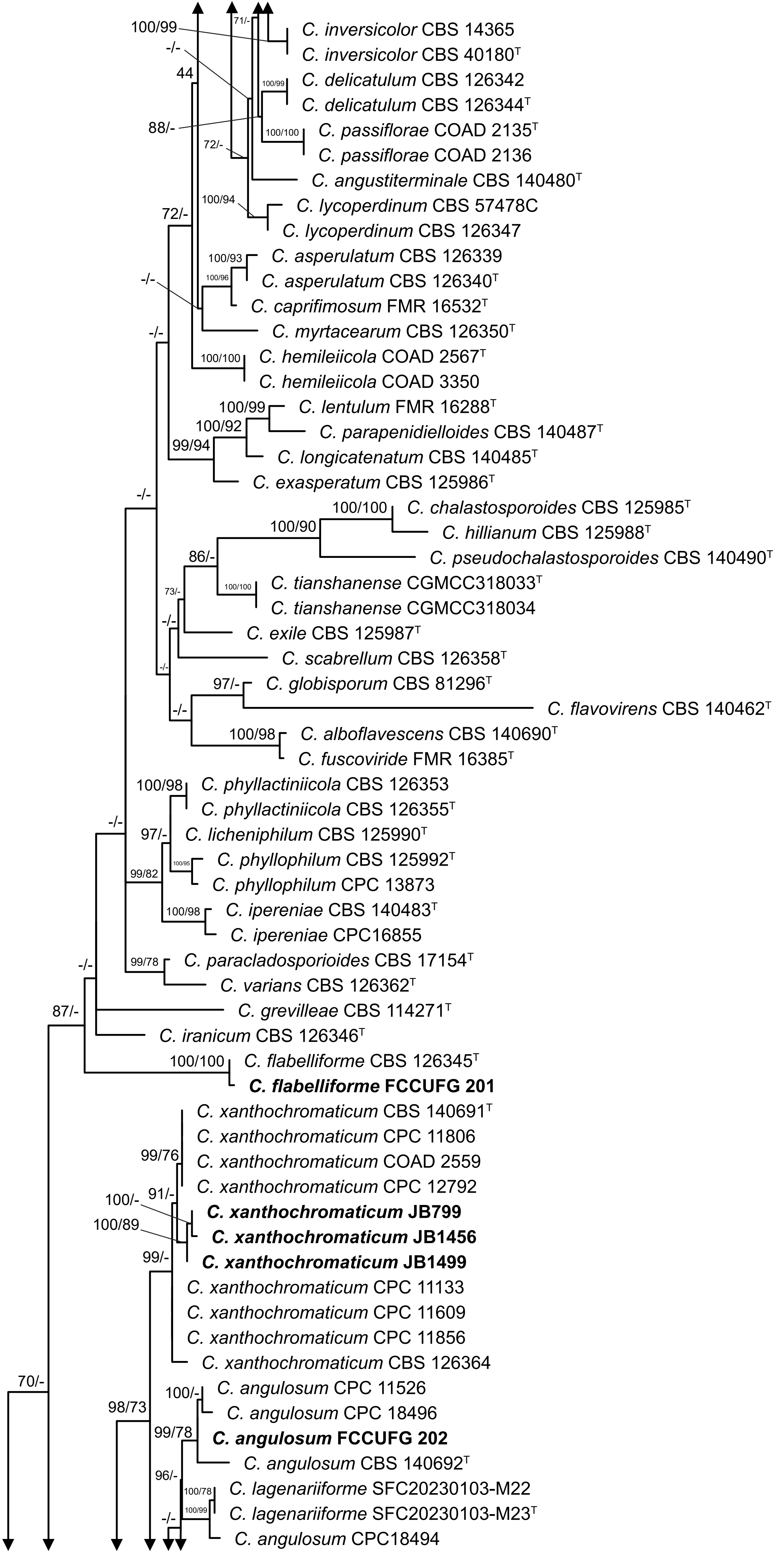
C. inversicolor CBS 14365
C. inversicolor CBS 40180T
C. delicatulum CBS 126342
C. delicatulum CBS 126344T
C. passiflorae COAD 2135T
C. passiflorae COAD 2136
C. angustiterminale CBS 140480T
C. lycoperdinum CBS 57478C
C. lycoperdinum CBS 126347
C. asperulatum CBS 126339
C. asperulatum CBS 126340T
C. caprifimosum FMR 16532T
C. myrtacearum CBS 126350T
C. hemileiicola COAD 2567T
C. hemileiicola COAD 3350
C. lentulum FMR 16288T
C. parapenidielloides CBS 140487T
C. longicatenatum CBS 140485T
C. exasperatum CBS 125986T
C. chalastosporoides CBS 125985T
C. hillianum CBS 125988T
C. pseudochalastosporoides CBS 140490T
C. tianshanense CGMCC318033T
C. tianshanense CGMCC318034
C. exile CBS 125987T
C. scabrellum CBS 126358T
C. globisporum CBS 81296T
C. flavovirens CBS 140462T
C. alboflavescens CBS 140690T
C. fuscoviride FMR 16385T
C. phyllactiniicola CBS 126353
C. phyllactiniicola CBS 126355T
C. licheniphilum CBS 125990T
C. phyllophilum CBS 125992T
C. phyllophilum CPC 13873
C. ipereniae CBS 140483T
C. ipereniae CPC16855
C. paracladosporioides CBS 17154T
C. varians CBS 126362T
C. grevilleae CBS 114271T
C. iranicum CBS 126346T
C. flabelliforme CBS 126345T
C. flabelliforme FCCUFG 201
C. xanthochromaticum CBS 140691T
C. xanthochromaticum CPC 11806
C. xanthochromaticum COAD 2559
C. xanthochromaticum CPC 12792
C. xanthochromaticum JB799
C. xanthochromaticum JB1456
C. xanthochromaticum JB1499
C. xanthochromaticum CPC 11133
C. xanthochromaticum CPC 11609
C. xanthochromaticum CPC 11856
C. xanthochromaticum CBS 126364
C. angulosum CPC 11526
C. angulosum CPC 18496
C. angulosum FCCUFG 202
C. angulosum CBS 140692T
C. lagenariiforme SFC20230103-M22
C. lagenariiforme SFC20230103-M23T
C. angulosum CPC18494

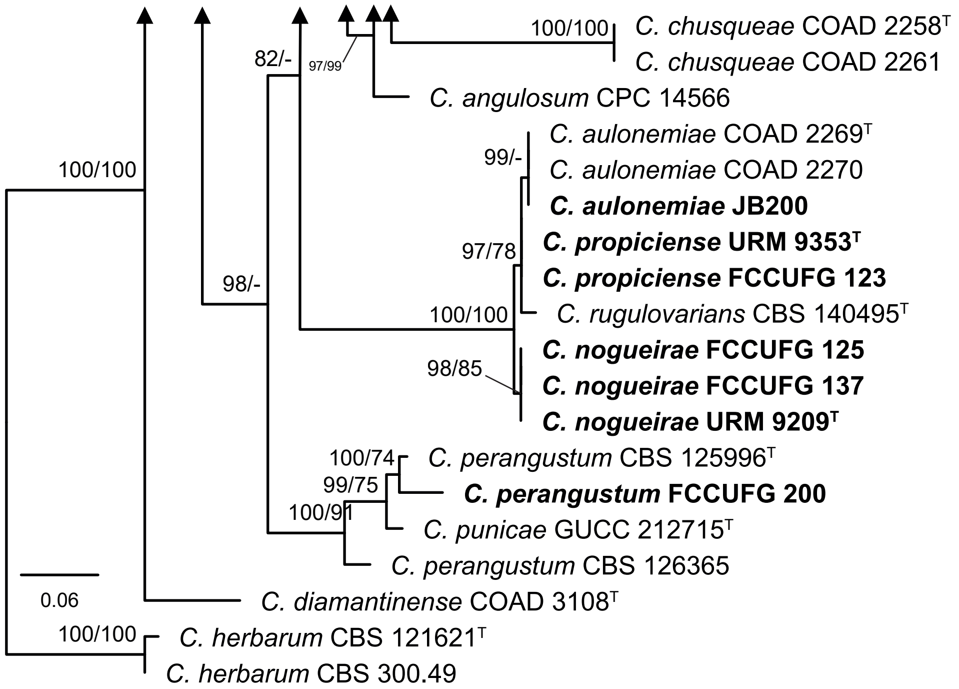

Figure S1. Maximum-likelihood IQTree tree of *C. cladosporioides* SC based on an individual dataset of *ACT* sequences. The species obtained in this study are highlighted in **bold**. Ex-type strains = T. IQ-TREE-BS values ≥ 70% and RAxML-BS ≥ 70% are included next to the nodes. The tree was rooted with *Cladosporium herbarum* (CBS 121621 and CBS 300.49).
