# Supplementary material for: Cladosporium from caves of the Brazilian savannah (Cerrado) and the description of six new species
Source: IMA Fungus. 2026 Jun 3;17:e191673. doi: 10.3897/imafungus.17.191673 (PMC13254554; doi:10.3897/imafungus.17.191673)
Supplement: Supplementary material 2 — Supplementary image 2 [file imafungus-17-e191673-s002.pdf]

***C. chlamydosporiformans* FCCUFG 130**
***C. macadamiae* FCCUFG 135**
C. vicinum CBS 143366^{T}
C. angustiterminale CBS 140480^{T}
***C. mambaiense* URM 9104^{T}**
***C. chlamydosporiformans* FCCUFG 115**
C. pseudocladosporioides CBS 125993^{T}
C. macadamiae BRIP 72287a
C. angustisporum CPC 22371
C. brigadeirensis COAD 2257^{T}
***C. wenganense* FCCUFG 117**
C. proteacearum SFC20230103-M53
C. angustisporum CPC 22345
C. subuliforme UTHSC DI13214
C. pseudocladosporioides CBS 66780
C. chlamydosporiformans COAD 2568
C. funiculosum CBS 122128
C. chlamydosporiformans COAD 2561
C. sinuatum CGMCC318096^{T}
***C. chlamydosporiformans* FCCUFG 74**
C. neopsychrotolerans CGMCC318031^{T}
***C. carsi* FCCUFG 146**
***C. mambaiense* FCCUFG 147**
C. chlamydosporiformans COAD 2571^{T}
***C. carsi* URM 9208^{T}**
C. needhamense CBS 143359^{T}
C. marinum SFC20230103-M33^{T}
C. sinuatum CGMCC318097
C. proteacearum BRIP 72301a^{T}
C. eucommiae GUCC 4011^{T}
C. austroafricanum CBS140481^{T}
***C. chlamydosporiformans* FCCUFG 133**
C. proteacearum SFC20230103-M54
C. europaeum CBS 116744
C. puris COAD 2487^{T}
***C. chlamydosporiformans* FCCUFG 75**
C. proteacearum SFC20230103-M55
C. uwebraunianum CBS 143365^{T}
C. delicatulum CBS 126342
***C. chlamydosporiformans* FCCUFG 78**
C. puris COAD 2566
C. nayongense GUCC 212603^{T}
C. australiense CBS 125984^{T}
C. eucommiae GUCC 4019
C. uwebraunianum DTO305H9
C. macadamiae BRIP 72269a^{T}
C. angustisporum DTO 127E6
***C. chlamydosporiformans* FCCUFG 77**
C. montecillanum CBS 140486^{T}
***C. chlamydosporiformans* FCCUFG 68**
C. angustisporum UTHSC DI13240
C. cladosporioides CBS 112388^{T}
***C. macadamiae* FCCUFG 116**
***C. macadamiae* FCCUFG 67**
C. westerdijkiae CPC 10150
C. montecillanum CPC 15605

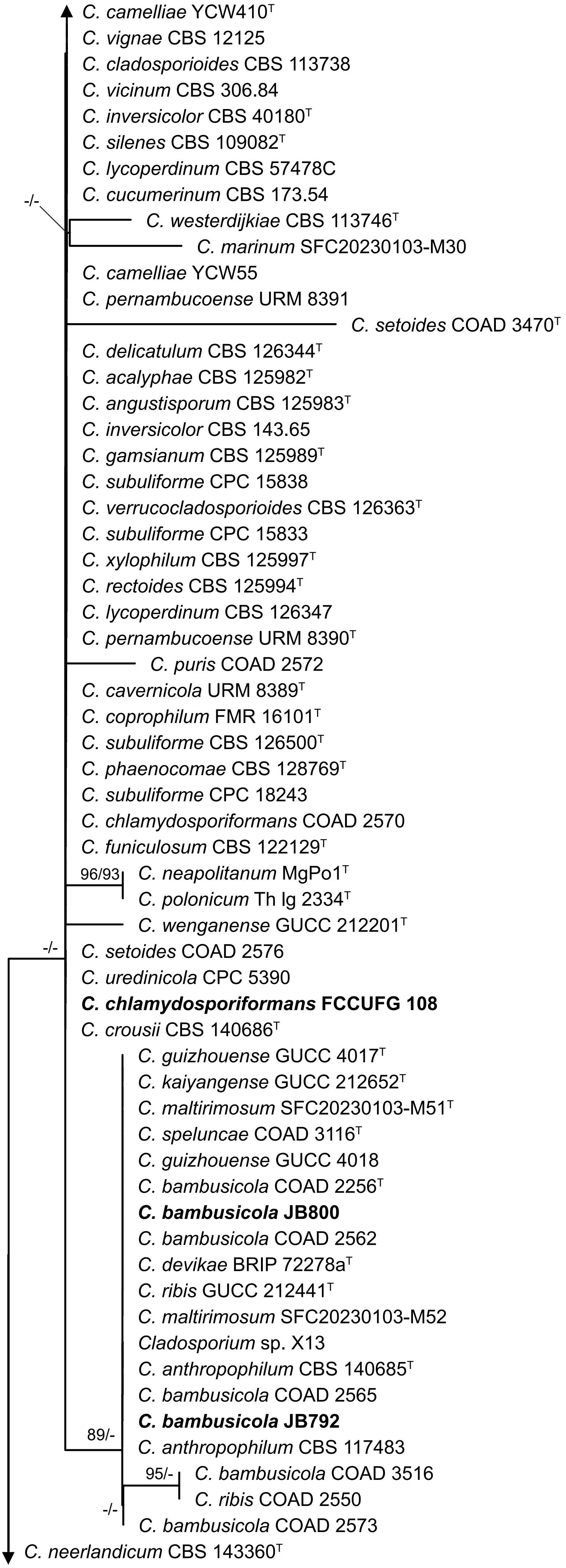
C. camelliae YCW410^{T}
C. vignae CBS 12125
C. cladosporioides CBS 113738
C. vicinum CBS 306.84
C. inversicolor CBS 40180^{T}
C. silenes CBS 109082^{T}
C. lycoperdinum CBS 57478C
C. cucumerinum CBS 173.54
-/-
C. westerdijkiae CBS 113746^{T}
C. marinum SFC20230103-M30
C. camelliae YCW55
C. pernambucoense URM 8391
C. setoides COAD 3470^{T}
C. delicatulum CBS 126344^{T}
C. acalyphae CBS 125982^{T}
C. angustisporum CBS 125983^{T}
C. inversicolor CBS 143.65
C. gamsianum CBS 125989^{T}
C. subuliforme CPC 15838
C. verrucocladosporioides CBS 126363^{T}
C. subuliforme CPC 15833
C. xylophilum CBS 125997^{T}
C. rectoides CBS 125994^{T}
C. lycoperdinum CBS 126347
C. pernambucoense URM 8390^{T}
C. puris COAD 2572
C. cavernicola URM 8389^{T}
C. coprophilum FMR 16101^{T}
C. subuliforme CBS 126500^{T}
C. phaenocomae CBS 128769^{T}
C. subuliforme CPC 18243
C. chlamydosporiformans COAD 2570
C. funiculosum CBS 122129^{T}
96/93
C. neapolitanum MgPo1^{T}
C. polonicum Th Ig 2334^{T}
C. wenganense GUCC 212201^{T}
-/-
C. setoides COAD 2576
C. uredinicola CPC 5390
C. chlamydosporiformans FCCUFG 108
C. crousii CBS 140686^{T}
C. guizhouense GUCC 4017^{T}
C. kaiyangense GUCC 212652^{T}
C. maltirimosum SFC20230103-M51^{T}
C. speluncae COAD 3116^{T}
C. guizhouense GUCC 4018
C. bambusicola COAD 2256^{T}
C. bambusicola JB800
C. bambusicola COAD 2562
C. devikae BRIP 72278a^{T}
C. ribis GUCC 212441^{T}
C. maltirimosum SFC20230103-M52
Cladosporium sp. X13
C. anthropophilum CBS 140685^{T}
C. bambusicola COAD 2565
C. bambusicola JB792
89/-
C. anthropophilum CBS 117483
95/-
C. bambusicola COAD 3516
C. ribis COAD 2550
-/-
C. bambusicola COAD 2573
C. neerlandicum CBS 143360^{T}

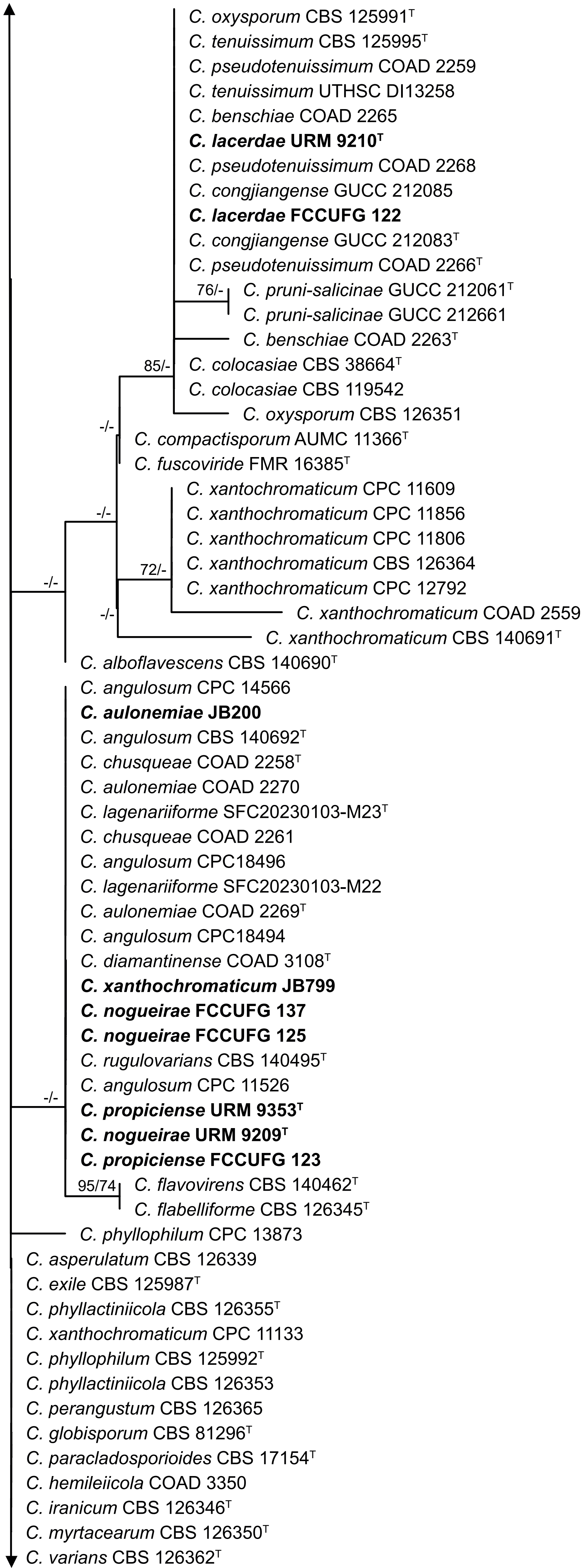
C. oxysporum CBS 125991T
C. tenuissimum CBS 125995T
C. pseudotenuissimum COAD 2259
C. tenuissimum UTHSC DI13258
C. benschiae COAD 2265
C. lacerdae URM 9210T
C. pseudotenuissimum COAD 2268
C. congjiangense GUCC 212085
C. lacerdae FCCUFG 122
C. congjiangense GUCC 212083T
C. pseudotenuissimum COAD 2266T
76/-
C. pruni-salicinae GUCC 212061T
C. pruni-salicinae GUCC 212661
C. benschiae COAD 2263T
85/-
C. colocasiae CBS 38664T
C. colocasiae CBS 119542
C. oxysporum CBS 126351
-/-
C. compactisporum AUMC 11366T
C. fuscoviride FMR 16385T
C. xantochromaticum CPC 11609
C. xanthochromaticum CPC 11856
-/-
C. xanthochromaticum CPC 11806
C. xanthochromaticum CBS 126364
72/-
C. xanthochromaticum CPC 12792
-/-
-/-
C. xanthochromaticum COAD 2559
C. xanthochromaticum CBS 140691T
C. alboflavescens CBS 140690T
C. angulosum CPC 14566
C. aulonemiae JB200
C. angulosum CBS 140692T
C. chusqueae COAD 2258T
C. aulonemiae COAD 2270
C. lagenariiforme SFC20230103-M23T
C. chusqueae COAD 2261
C. angulosum CPC18496
C. lagenariiforme SFC20230103-M22
C. aulonemiae COAD 2269T
C. angulosum CPC18494
C. diamantinense COAD 3108T
C. xanthochromaticum JB799
C. nogueirae FCCUFG 137
C. nogueirae FCCUFG 125
C. rugulovarians CBS 140495T
C. angulosum CPC 11526
-/-
C. propiciense URM 9353T
C. nogueirae URM 9209T
C. propiciense FCCUFG 123
95/74
C. flavovirens CBS 140462T
C. flabelliforme CBS 126345T
C. phyllophilum CPC 13873
C. asperulatum CBS 126339
C. exile CBS 125987T
C. phyllactiniicola CBS 126355T
C. xanthochromaticum CPC 11133
C. phyllophilum CBS 125992T
C. phyllactiniicola CBS 126353
C. perangustum CBS 126365
C. globisporum CBS 81296T
C. paracladosporioides CBS 17154T
C. hemileiicola COAD 3350
C. iranicum CBS 126346T
C. myrtacearum CBS 126350T
C. varians CBS 126362T

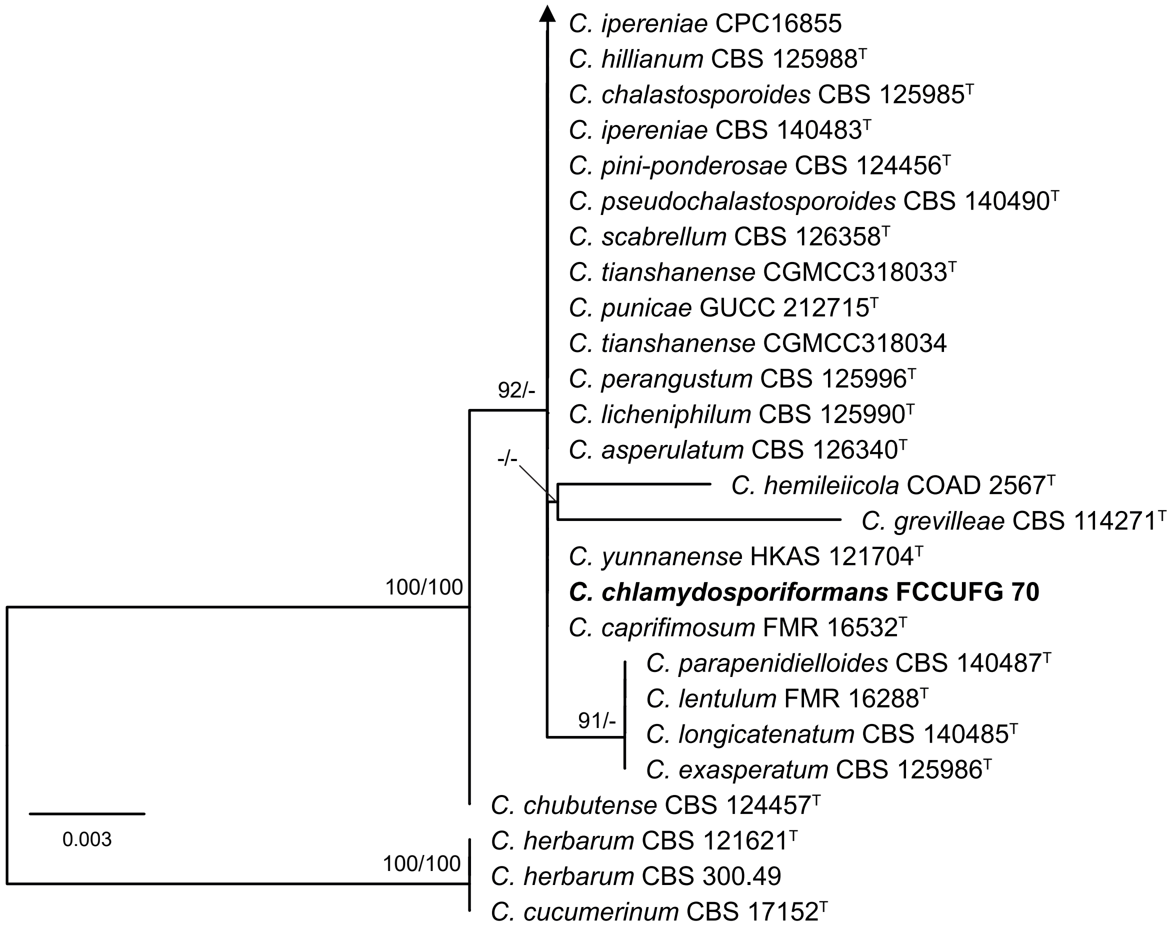

Figure S2. Maximum-likelihood IQTree tree of *C. cladosporioides* SC based on an individual dataset of ITS sequences. The species obtained in this study are highlighted in **bold**. Ex-type strains = T. IQ-TREE-BS values ≥ 70% and RAxML-BS ≥ 70% are included next to the nodes. The tree was rooted with *Cladosporium herbarum* (CBS 121621 and CBS 300.49).
