# Supplementary figures and images for: Cladosporium from caves of the Brazilian savannah (Cerrado) and the description of six new species
Source: IMA Fungus. 2026 Jun 3;17:e191673. doi: 10.3897/imafungus.17.191673 (PMC13254554; doi:10.3897/imafungus.17.191673)

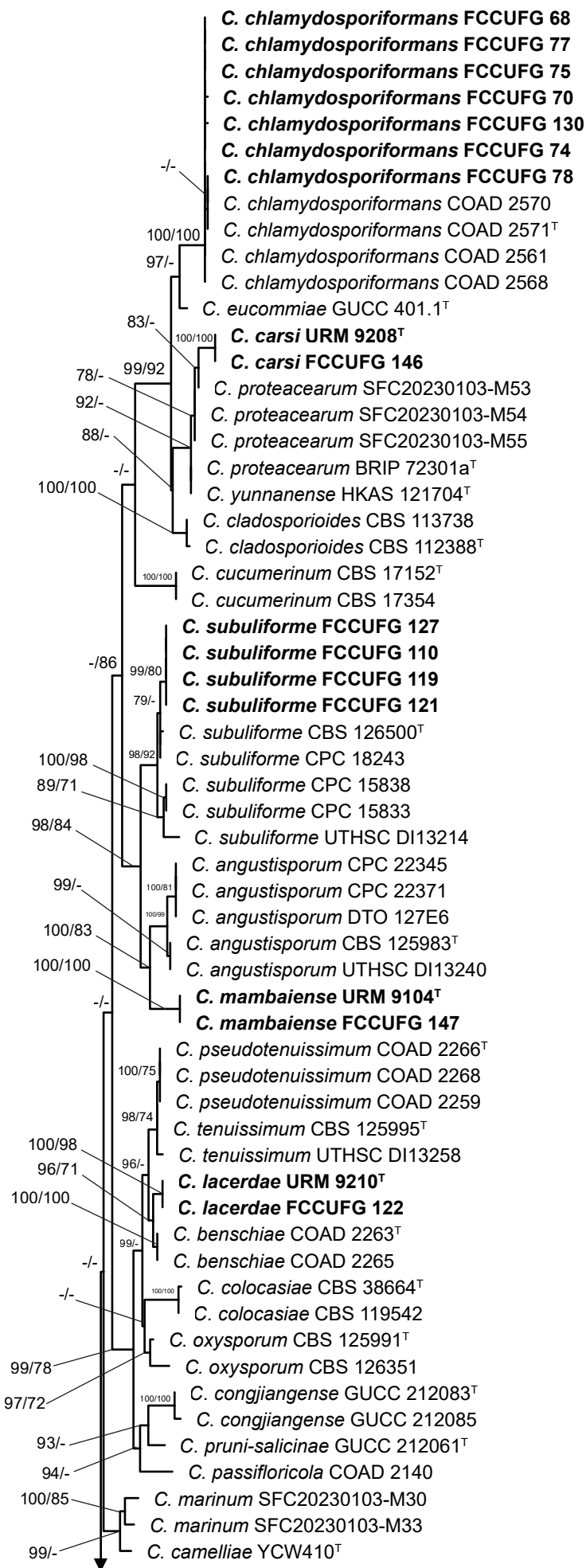

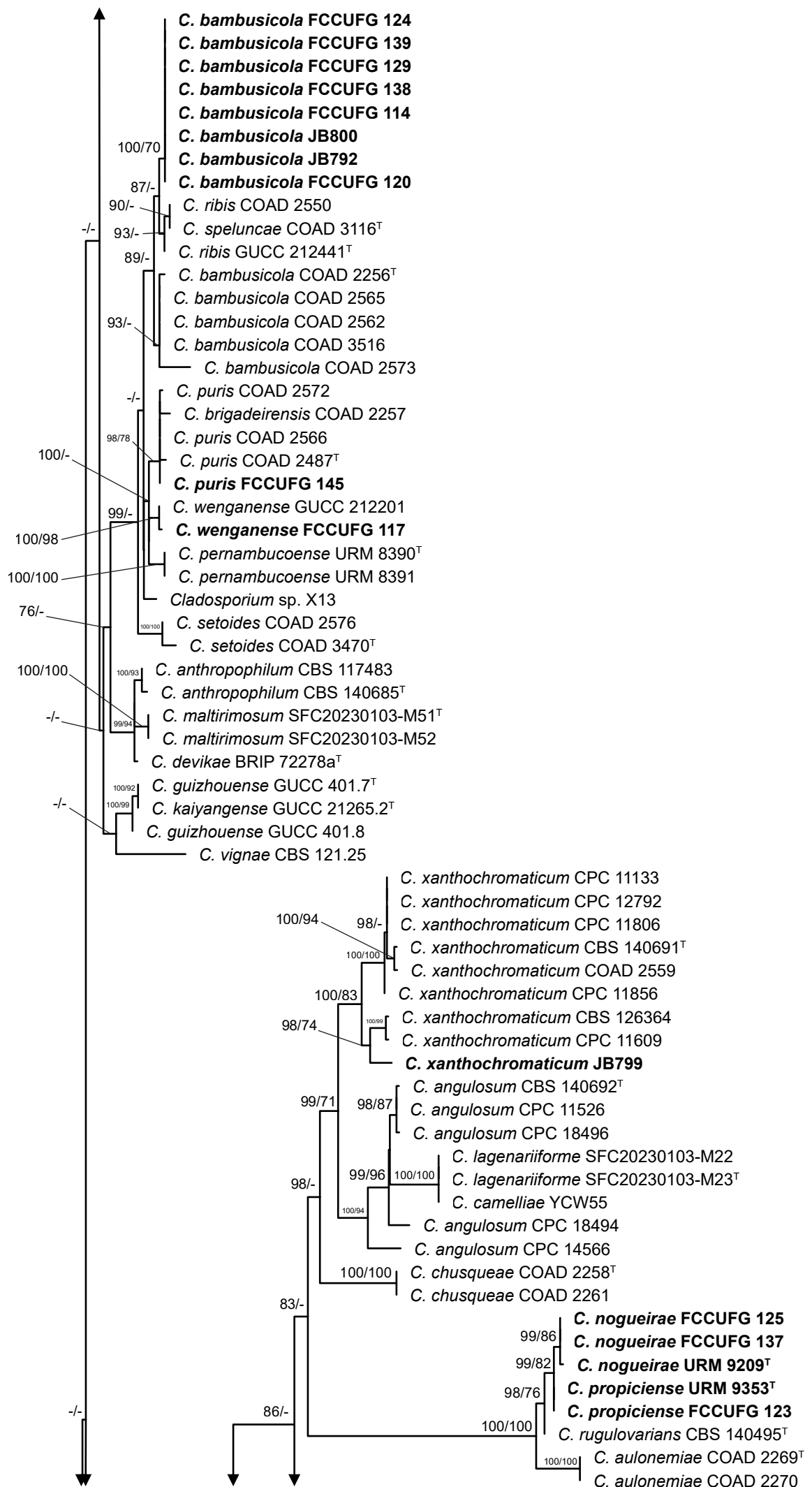

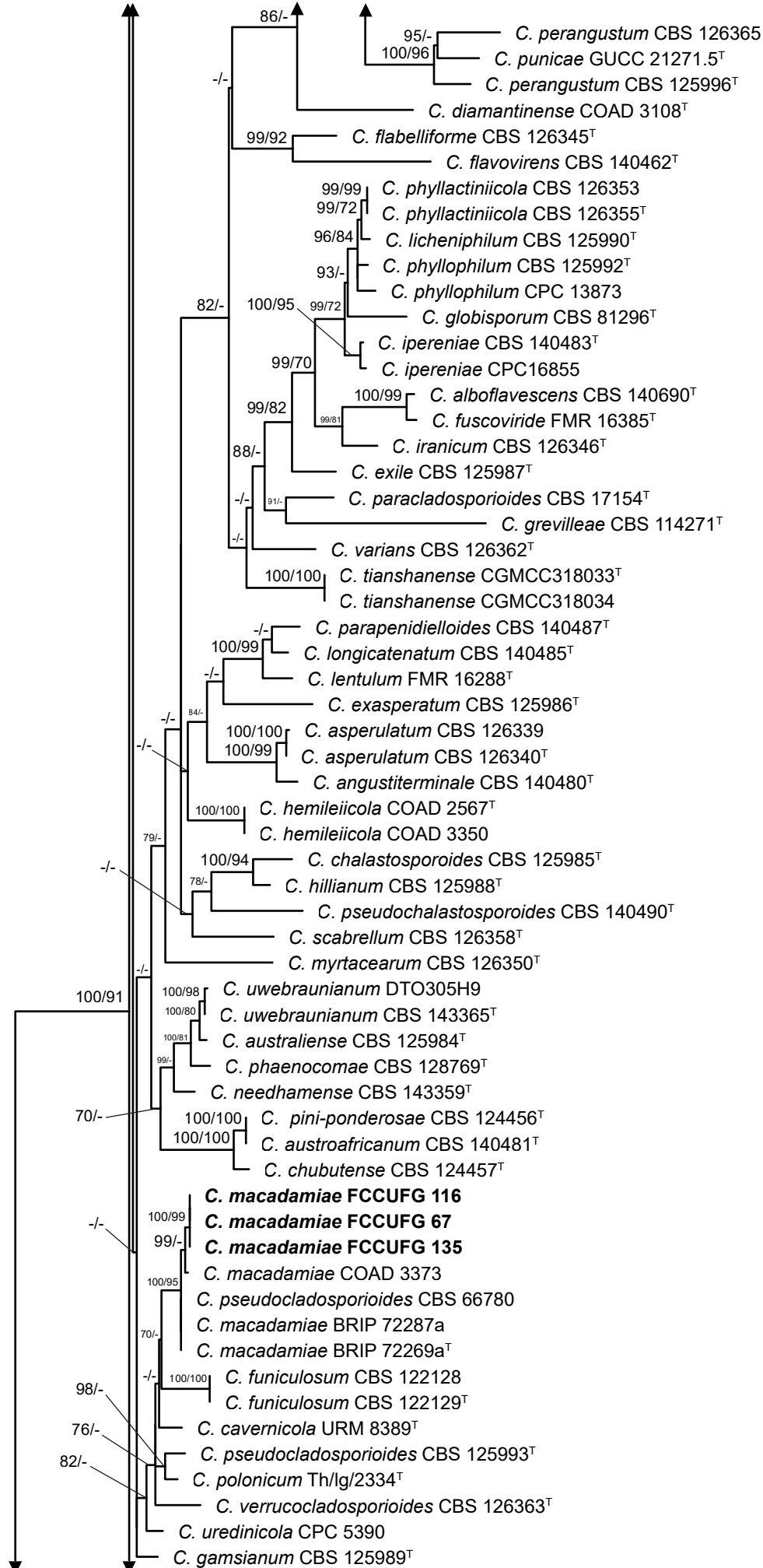

Supplement: Supplementary material 3 — Supplementary image 3 [file imafungus-17-e191673-s003.pdf]
