# Supplementary material for: Cladosporium from caves of the Brazilian savannah (Cerrado) and the description of six new species
Source: IMA Fungus. 2026 Jun 3;17:e191673. doi: 10.3897/imafungus.17.191673 (PMC13254554; doi:10.3897/imafungus.17.191673)
Supplement: Supplementary material 4 — Supplementary image 4 [file imafungus-17-e191673-s004.pdf]

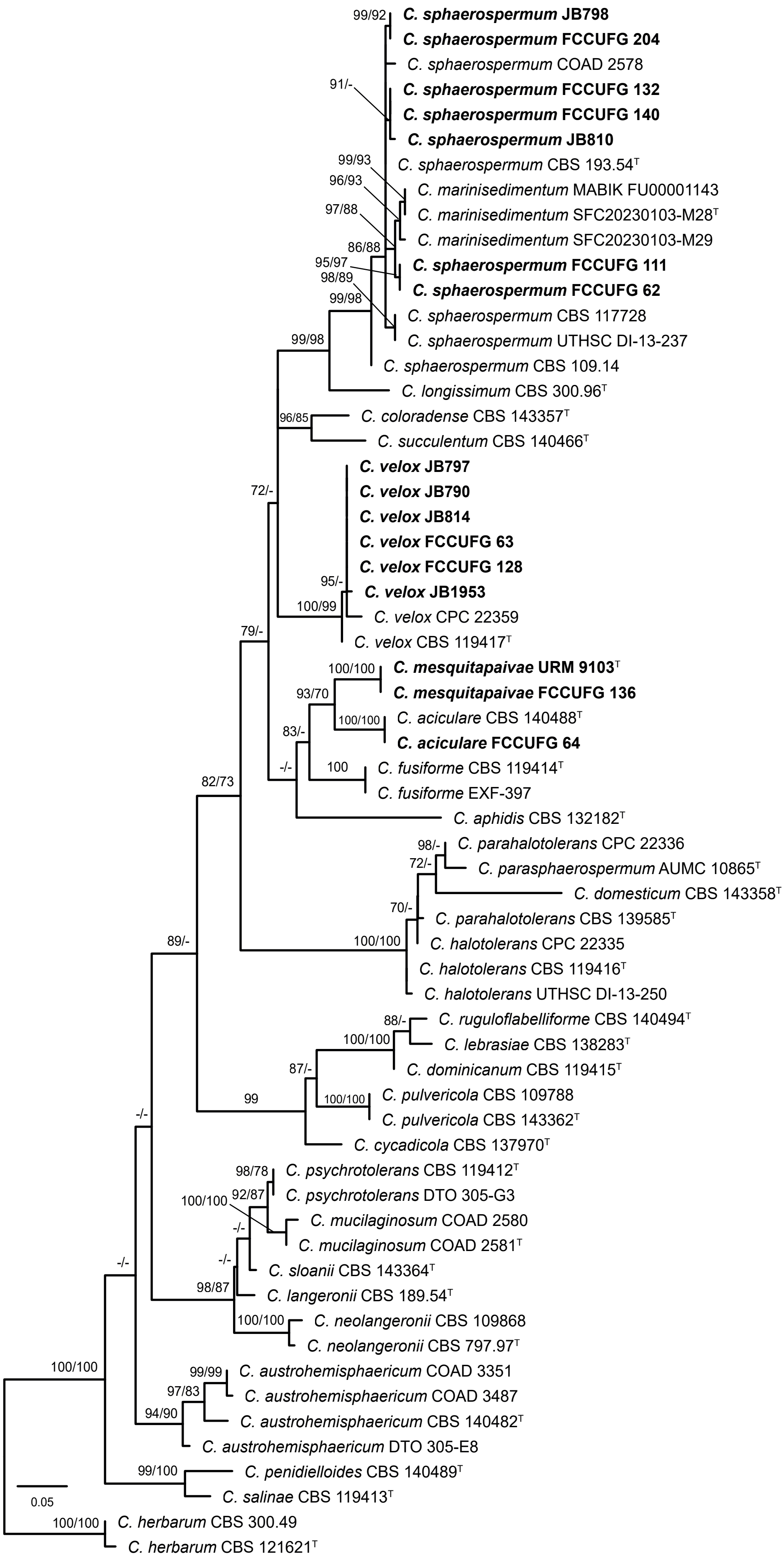

Figure S4. Maximum-likelihood IQTree tree of *C. sphaerospermum* SC based on an individual dataset of *ACT* sequences. The species obtained in this study are highlighted in **bold**. Ex-type strains = T. IQ-TREE-BS values ≥ 70% and RAxML-BS ≥ 70% are included next to the nodes. The tree was rooted with *Cladosporium herbarum* (CBS 121621 and CBS 300.49).
