# Supplementary material for: Cladosporium from caves of the Brazilian savannah (Cerrado) and the description of six new species
Source: IMA Fungus. 2026 Jun 3;17:e191673. doi: 10.3897/imafungus.17.191673 (PMC13254554; doi:10.3897/imafungus.17.191673)
Supplement: Supplementary material 9 — Supplementary tables [file imafungus-17-e191673-s009.pdf]

Table S1. Summary of the characteristics of the studied caves, including geographic coordinates, number of collection points, count of fungal isolates by substrate (air and soil), and the respective temperature and humidity data.

Caves	Lithology	Latitude	Longitude	Points	Isolates (air)	Isolates (soil)	Temperature (ºC)	Humidity (%)
Lapa do Penhasco	Limestone from the Bambuí Group	14°26'10" S	46°13'35" W	6	11	5	22	75-80
Lapa do Córrego das Dores	Limestone from the Bambuí Group	14°25'45" S	46°13'10" W	5	24	0	25	43-56
Lapa da Cachoeira do Funil	Limestone from the Bambuí Group	14°28'32" S	46°09'04" W	3	5	1	24	74-79
Gruna da Tarimba	Limestone from the Bambuí Group	14°24'43" S	46°10'30" W	6	15	1	25	80
Lapa do Boqueirão	Limestone from the Araxá Group	15°24'34" S	48°43'57" W	8	21	4	19-21	77-86
Garganta (Samambaia)	Limestone from the Araxá Group	15º22'54" S	48º41'55" W	10	7	0	19-21	77-86

Table S2. Checklist of *Cladosporium* species documented in caves from different Brazilian biomes, with details on the location, substrate, and reference for each record.

Year	Species	Biome	Cave	Substrate	Reference
2010	*Cladosporium* sp.	Cerrado	GPM1 and GPM2	Soil/Sediment	Silva et al.
2013	*C. cladosporioides*	Cerrado	Lapa Nova	Air and Guano	Taylor et al.
2014	*C. cladosporioides*	Cerrado	RM3	Soil/Sediment	Taylor et al.
2015	*C. cladosporioides* and *Cladosporium* sp.	Cerrado	Gruta da Lapinha, Gruta da Macumba, Gruta dos Túneis and Gruta DOC	Soil/Sediment	Costa
2018	*Cladosporium* sp.	Cerrado	Angélica, Terra Ronca II and São Bernardo	Soil/Sediment	Paula
2019	*C. cladosporioides*	Cerrado	Gruta Lagoa Azul	Soil/Sediment	GEEP Açungui
2019	*C. cladosporioides*	Cerrado	Gruta Nossa Senhora Aparecida	Soil/Sediment	GEEP Açungui
2019	*Cladosporium* sp. and *C. tenuissimum*	Caatinga	Meu Rei	Air, Bat and Guano	Cunha
2020	*Cladosporium* sp. and *C.anthropophilium*	Cerrado	Monte Cristo	Air, Soil/Sediment, Guano and Rocks	Dutra
2020	*Cladosporium* sp. 1 and *Cladosporium* sp. 2 *C. sphaerospermum* complex, and *Cladosporium* sp. 3 *C. cladosporioides* complex	Caatinga	Meu Rei	Air, Bat and Guano	Cunha et al.
2021	*Cladosporium* sp.	Cerrado	Gruta Velha Nova	Air, Soil/Sediment, Leaf litter and Rocks	Leão
2021	*C. sphaerospermum*, *C. halotolerans*, and *Cladosporium* sp.	Cerrado	Lapa de Terra Ronca I	Soil/Sediment	De Paula et al.
2022	*C. halotolerans* and *C. subuliforme*	Caatinga	Furna do Morcego	Ectoparasitic bat flies	Carvalho et al.

2022	*C. cavernicola*, *C. pernambucoense*, *C. puris*, *C. subuliforme*, *C. tenuissimum*, *C. austrohemisphaericum*, *C. parahalotolerans*, and *C. sphaerospermum*	Caatinga	Furna do Morcego	Air and ectoparasitic bat flies	Pereira et al.
2022	*C. oxysporum*, *C. subuliforme*, *C. tenuissimum*, and *C. xanthocromaticum*	Caatinga	Abrigo do letreiro	Air and sediment	Alves et al.
2023	*C. halotolerans*	Caatinga	Catedral	Soil/Sediment	Lira
2023	*C. diamantinense* and *C. speluncae*	Cerrado	Monte Cristo	Air and Soil/Sediment	Dutra et al.
2024	*Cladosporium* sp.	Caatinga and Atlantic Forest	Furna Feia and Urubu	Air, Bat and Soil/Sediment	Lima et al.
2026	*C. angulosum*, *C. aulonemiae*, *C. bambusicola*, *C. carsi* sp. nov., *C. chlamydosporiformans*, *C. flabelliforme*, *C. lacerdae* sp. nov., *C. macadamiae*, *C. mambaiense* sp. nov., *C. nogueirae* sp. nov., *C. perangustum*, *C. pernambucoense*, *C. pruni-salicinae*, *C. propiciense* sp. nov., *C. pseudocladosporioides*, *C. puris*, *C. subuliforme*, *C. wenganense*, *C. xanthochromaticum* *C. aciculare*, *C. mesquitapaivae* sp. nov., *C. sphaerospermum*, and *C. velox*	Cerrado	Lapa do Penhasco, Lapa do Córrego das Dores, Lapa da Cachoeira do Funil, Gruna da Tarimba, Lapa do Boqueirão and Garganta (Samambaia)	Air and Soil	This study

References

Alves VCS, Lira RA, Lima JMS et al. (2022) Unravelling the fungal darkness in a tropical cave: richness and the description of one new genus and six new species. Fungal Systematics and Evolution 10(1): 139–167. https://doi.org/10.3114/fuse.2022.10.06

Carvalho JLVR, Lima JMS, Barbier E et al. (2022) Ticket to ride: fungi from bat ectoparasites in a tropical cave and the description of two new species. Brazilian Journal of Microbiology 53: 2077–2091. https://doi.org/10.1007/s42770-022-00841-y

Costa FLB (2015) Identificação de fungos isolados de cavidades naturais subterrâneas do Parque Estadual do Sumidouro. PhD Thesis, Universidade Federal de Minas Gerais, Minas Gerais, Brazil.

Cunha AOB, Bezerra JDP, Oliveira TGL et al. (2020) Living in the dark: Bat caves as hotspots of fungal diversity. Plos One 15(12): e0243494. https://doi.org/10.1371/journal.pone.0243494

Cunha AOB (2019) Caracterização taxonômica da micobiota de uma Bat cave no PARNA do Catimbau, Pernambuco, Brasil. Master's Dissertations, Universidade Federal de Pernambuco, Pernambuco, Brazil.

Dutra YLG (2020) Taxonomia e filogenia molecular de fungos da caverna Monte Cristo na Serra do Espinhaço Meridional (Diamantina - Minas Gerais) Master's Dissertations, Universidade Federal Viçosa, Minas Gerais, Brazil.

Dutra YLG, Rosado AWC, Condé TO et al. (2023) Two new *Cladosporium* species from a quartzite cave in Brazil. Brazilian Journal of Microbiology 54: 3021–3031. https://doi.org/10.1007/s42770-023-01156-2

Leão AF (2021)Fungos cavernícolas da Gruta Velha Nova na Serra do Espinhaço em Monjolos, Minas Gerais-Brasil. Master's Dissertations, Universidade Federal Viçosa, Minas Gerais, Brazil.

Lima JMS, Barbosa RN, Bento DM et al. (2024) *Aspergillus*, *Penicillium*, and *Talaromyces* (*Eurotiales*) in Brazilian caves, with the description of four new species. Fungal Systematics and Evolution 14(1): 89–107. https://doi.org/10.3114/fuse.2024.14.06

Lira RA (2023) Micobiota do sedimento da caverna Catedral, Felipe Guerra-RN, Brasil. Undergraduate course completion papers, Universidade Federal de Pernambuco, Pernambuco, Brazil.

Paula CCP, Montoya QV, Rodrigues A et al. (2016) Terrestrial filamentous fungi from Gruta do Catão (São Desidério, Bahia, Northeastern Brazil) show high levels of cellulose degradation. Journal of Cave and Karst Studies 78(3): 208–217. doi:10.4311/2016MB0100

Paula CCP (2018) Dinâmica e diversidade das comunidades microbianas em cavernas tropicais do Brasil Central. PhD Thesis, Universidade Federal de São Carlos, São Paulo, Brazil.

Pereira MLS, Carvalho JLVR, Lima JMS et al. (2022) Richness of *Cladosporium* in a tropical bat cave with the description of two new species. Mycological Progress 21: 345–357. https://doi.org/10.1007/s11557-021-01760-2

Silva L, Casali AK, Faria LE (2019) Levantamento e caracterização de fungos filamentosos de duas cavernas em canga laterítica no Parque Municipal das Mangabeiras, Belo Horizonte, Minas Gerais. Undergraduate course completion papers, Centro Universitário UNA, Minas Gerais, Brazil

Taylor ELS, Stoianoff MAR, Ferreira LR (2013) Mycological study for a management plan of a neotropical show cave (Brazil). International Journal of Speleology 2(3): 267–277. http://dx.doi.org/10.5038/1827-806X.42.3.10

Taylor ELS, Ferreira LR, Cardoso PG et al. (2014) Cave entrance dependent spore dispersion of filamentous fungi isolated from various sediments of iron ore cave in Brazil: a colloquy on human threats while caving. Ambient Science 1(1): 16–28. http://dx.doi.org/10.21276/ambi.2014.01.1.ra02
